# Recent Advances in Heterogeneous Frustrated Lewis Pair: Synthesis, Characterization, and Catalysis

**DOI:** 10.1002/adma.202502101

**Published:** 2025-06-20

**Authors:** Jiasi Li, Shik Chi Edman Tsang, Guangchao Li

**Affiliations:** ^1^ Wolfson Catalysis Centre Department of Chemistry University of Oxford Oxford OX1 3QR UK; ^2^ Department of Applied Biology and Chemical Technology The Hong Kong Polytechnic University Hong Kong 999077 China; ^3^ Research Institute of Advanced Manufacturing (RIAM) Research Centre for Resources Engineering toward Carbon Neutrality (RCRE) The Hong Kong Polytechnic University Hong Kong 999077 China; ^4^ The Hong Kong Polytechnic University Shenzhen Research Institute The Hong Kong Polytechnic University Shenzhen 518057 China; ^5^ PolyU‐Daya Bay Technology and Innovation Research Institute The Hong Kong Polytechnic University Huizhou 516000 China; ^6^ Crystallography Group Diamond Light Source Diamond House Harwell Science and Innovation Campus, Fermi Avenue Didcot OX11 0DE U.K.

**Keywords:** advanced characterization, frustrated Lewis pair, heterogenous catalysis, small molecules activation

## Abstract

Frustrated Lewis pair (FLP) in heterogeneous chemistry have garnered tremendous attention in recent years owing to their diverse structural designs and outstanding activation ability for small molecules. The ability to tailor the structure of FLP enables precise control over their reactivity and selectivity, paving the way for the creation of catalysts for specific reactions. This review offers an in‐depth examination of the design, characterization, and application of FLP within heterogeneous systems over the past few years. The current challenges in developing solid FLP catalysts are discussed. Furthermore, future potential advancements are explored, considering how emerging technologies and innovative approaches could enhance the design, advance characterization, and application of FLP in heterogeneous chemistry. Through this detailed overview, it is aimed to provide valuable insights into the evolving landscape of FLP research and its implications for the future applications in catalysis.

## Graphical abstract

This review provides a concise analysis of heterogeneous frustrated Lewis pair (FLP) chemistry, focusing on their synthesis, characterization, and application in small‐molecule activation. It highlights current challenges in developing solid FLP systems and explores promising advancements through emerging technologies, offering critical insights into the rapidly evolving field of FLP research and its future potential in catalysis.

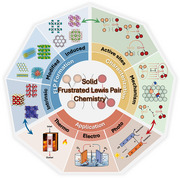

## 1 Introduction

**Figure 1:**
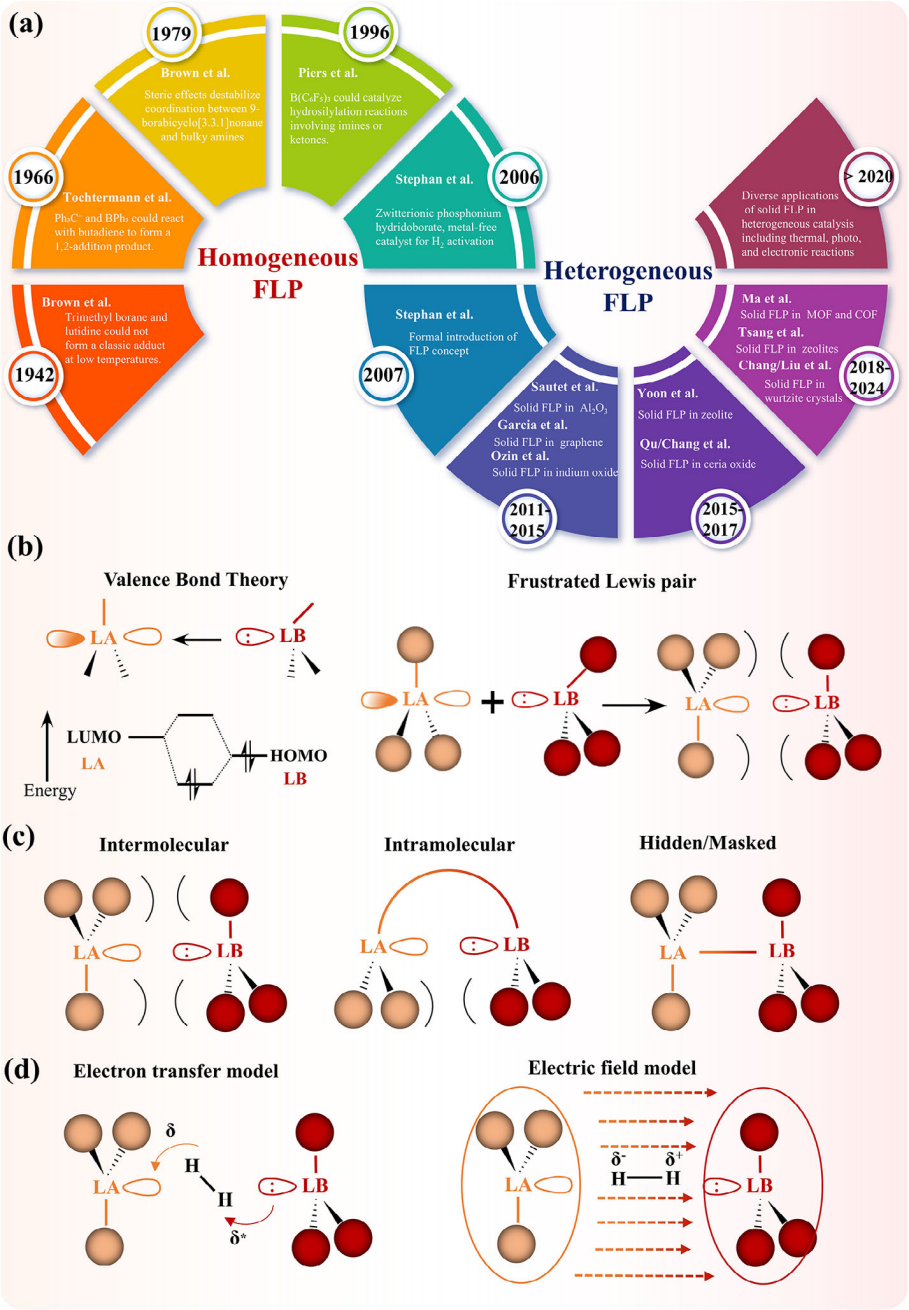
a) Development of FLP from homogeneous to heterogeneous system. b) The definition, c) types, and (d) mechanisms of FLP chemistry.

Catalytic activity is significantly governed by the structural and electronic properties of active sites, which span diverse elemental compositions, geometries, and bonding configurations. Engineering these sites to steer specific reactions has thus become a cornerstone of sustainable catalyst development. Among the innovative approaches in this field, Frustrated Lewis Pair (FLP) has emerged as a prominent area of research and received a lot of attention over the past two decades, offering unique advantages such as the ability to activate small molecules under mild conditions.^[^ ^1^ ^]^ The FLP concept originated from studies of sterically hindered phosphine donors and electrophilic boranes that activate H₂ without forming classical Lewis adducts (CLA).^[^ ^2^ ^]^ This landmark discovery bridged homogeneous and heterogeneous catalysis by enabling metal‐free activation of inert chemical bonds, offering a unified framework for designing efficient catalytic systems. **Figure** **1**a traces the evolution of FLP chemistry, highlighting key milestones and their catalytic impact.

Acids and bases are fundamental to chemical reactions, which catalyze processes to form diverse products with regioselectivity, chemoselectivity, and stereoselectivity. The Lewis definition, introduced in 1923, describes acids as electron pair acceptors and bases as electron pair donors.^[^ ^3^ ^]^ The interaction between a Lewis acid (LA) and a Lewis base (LB) involving electron transfer depends on the energy difference between the LA's lowest unoccupied molecular orbital (LUMO) and the LB's highest occupied molecular orbital (HOMO) (Figure 1b). In general, this interaction with sufficiently overlapping orbitals forms a stable Lewis adduct. However, under certain conditions, this adduct formation can be impeded. This phenomenon was identified several decades ago. In 1942, Brown and colleagues first observed this unusual phenomenon, they found that trimethyl borane and lutidine could not form a classic adduct, even at low temperatures.^[^ ^4^ ^]^ In 1950, Wittig and coworkers discovered that instead of forming a Ph₃C‐BPh₃ adduct, a ring‐opening reaction of tetrahydrofuran (THF) occurred when BPh₃ and a THF adduct reacted with the Ph₃C⁻ anion.^[^ ^5^ ^]^ which highlighted alternative reaction pathways due to steric hindrance. Further exploration by Brown and Kanner in 1965 showed that steric hindrance affects the base strength of pyridine and its derivatives, influencing their ability to donate electrons or add protons.^[^ ^6^ ^]^ In 1966, Tochtermann found that Ph₃C⁻ and BPh₃ could react with butadiene to form a 1,2‐addition product, and described this non‐quenched pair as “antagonistisches Paar”.^[^ ^7^ ^]^ After that, Brown and Wang in 1979 summarized that steric effects destabilize coordination between 9‐borabicyclo[3.3.1]nonane and bulky amines.^[^ ^8^ ^]^ These findings suggest that bulky groups at LA and LB centers can open alternative reaction channels beyond classical adducts. In 1996, Piers and Parks demonstrated that B(C₆F₅)₃ could catalyze hydrosilylation reactions involving imines or ketones and Si─H bonds, showcasing FLP' catalytic potential.^[^ ^9^ ^]^ Two years later, Erker et al. observed unconventional reactivity in LA/LB complexes, where the phosphorus ylide adduct of B(C₆F₅)₃ dissociates under thermal stress, leading to the LB attack on the pentafluorophenyl ring, forming a linked phosphonium fluoroborate salt,^[^ ^10^ ^]^ underscoring the unique reactivity of LA/LB complexes.

In addition to earlier discoveries, a significant milestone in the study of LA and LB active centers in homogeneous catalysts was achieved in 2006. Stephan et al. synthesized a zwitterionic phosphonium hydridoborate, (C₆H₂Me₃)₂HP(C₆F₄)BH(C₆F₅)₂, which released H₂ gas upon heating above 100 °C, forming the ambiphilic phosphinoborane (C₆H₂Me₃)₂P(C₆F₄)B(C₆F₅)₂. Remarkably, this phosphinoborane was also reverted to the phosphonium hydridoborate by reacting with H_2_ at room temperature.^[^ ^2^ ^]^ This groundbreaking work demonstrated, for the first time, that a metal‐free catalyst could achieve reversible H₂ activation. Building on this foundation, Stephan et al., in 2007, further advanced the field by demonstrating the heterolytic cleavage of H₂ using a combination of tris‐(pentafluorophenyl) borane and tris‐(tert‐butyl) phosphine by unquenched acid and base system.^[^ ^11^ ^]^ This led to the introduction of the “Frustrated Lewis Pair” concept,^[^ ^12^ ^]^ which initially describes systems of which sterically hindered LA and LB are unable to form traditional adducts, thus opening alternative reaction pathways. Following these pioneering efforts, numerous FLP have been developed by varying the combinations of LA and LB. The scope of FLP has gradually expanded, with LA now including non‐metallic elements such as Si and C, as well as metallic elements like Al, In, Ga, and Sn. Similarly, LB has broadened to encompass elements such as C, N, O, S, and Te.^[^ ^13^ ^]^

During explorations, the concept of FLP‐type activation has been refined. In addition to separated (unquenched) sites, some classical Lewis pair systems in equilibrium were discovered to be FLP active.^[^ ^14^, ^15^ ^]^ In general, there are now three dominant types of FLP: intermolecular, intramolecular, and hidden or masked FLP (Figure 1c). For intermolecular FLP, which represents the traditional FLP, with the unquenched LA and LB centers from two different compounds.^[^ ^16^ ^]^ When in solution, the two compounds associate and form a loosely bound complex through secondary interactions, primarily London dispersion forces.^[^ ^17^, ^18^ ^]^ Balancing the steric bulkiness of substituents for appropriate separation distance is crucial in these FLP: if too bulky, repulsive interactions between the side groups render the two sites inactive.^[^ ^11^ ^]^ Conversely, if substituents are too small, LAs and LBs may form classic Lewis adducts, losing the “frustrated” nature. Similarly, in unquenched intramolecular FLP, although the LA and LB sites are now from a single molecule, there is no direct covalent bonding between the two sites.^[^ ^19^, ^20^ ^]^ There might be weak Van Der Waals forces between sites or not, depending on the linker in between. The third FLP type is the hidden or masked intramolecular FLP. The classical LA/LB pair adduct, which is in equilibrium with its separated form, can also perform traditional FLP‐like chemistry for small‐molecule activation.^[^ ^14^, ^15^, ^21^, ^22^ ^]^ In all categories of FLP systems, the geometric parameters and conformational flexibility of LA/LB play an essential role in the reactivity of FLP. Rigid LA/LB frameworks exhibit reduced activity due to the lower possibility of suitable conformers with functional FLP.^[^ ^23^ ^]^ The reaction pathway at FLP sites (in both homogeneous and heterogeneous systems) is illustrated by a two‐electron transfer mechanism, where the LA and LB act cooperatively to activate small molecules.^[^ ^24^, ^25^, ^26^ ^]^ Initially, the LA and LB form an “encounter complex” with weak forces in between. The adsorbates introduced are then heterolytically activated as the LB donates electron density from its lone pair into an antibonding orbital of the substrate, while the LA accepts electron density from a bonding orbital of the substrate. Furthermore, the electric field (EF) mechanism may significantly influence the reaction by initially polarizing the gas molecules before heterolytic cleavage (Figure 1d).^[^ ^27^ ^]^

**Figure 2:**
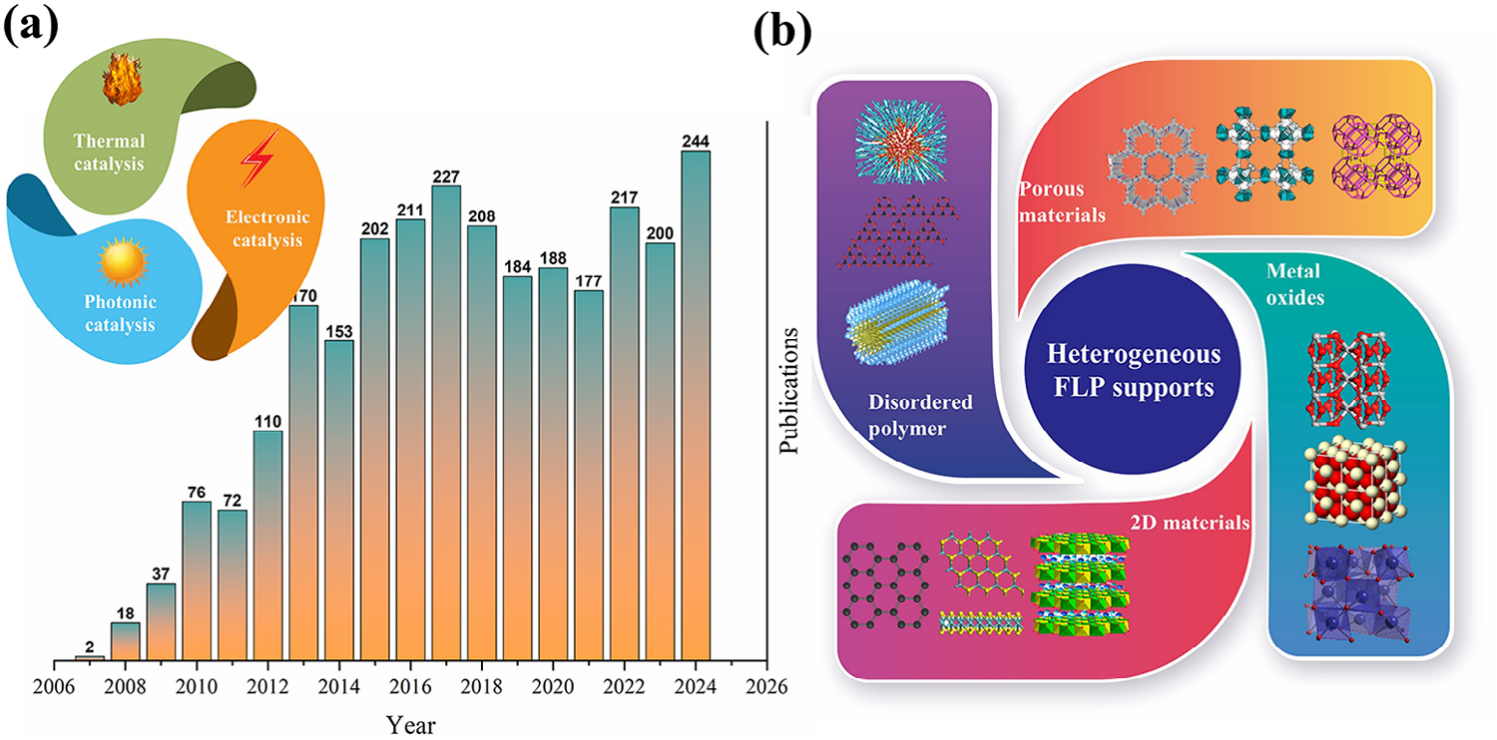
a) The annual publications of heterogeneous FLP chemistry over the past several years with different kinds of applications. b) The solid support materials for heterogeneous FLP construction.

The rapid advancement of FLP chemistry in the activation of small molecules (e.g., H_2_, CO_2_, monomers), for the synthesis of valuable chemicals, and polymers^[^ ^28^ ^]^ and other organic products, has established FLP as a significant area in homogeneous catalysis, and there are several excellent review papers discussing the progress and applications of homogeneous FLP.^[^ ^1^, ^29^, ^30^, ^31^, ^32^ ^]^ Furthermore, the extension of FLP chemistry to heterogeneous catalysis makes a significant advancement in the field. These heterogeneous FLP systems are diverse, ranging from co‐catalysts of some transition metal complexes to some modified solid supports. In this review, we will focus on the latter to explore the diverse solid surfaces for FLP‐type activation. The recent development of these supports is underscored by the exponential growth in publications (**Figure** **2**a). Both metal‐based and metal‐free heterogeneous FLP catalysts have been developed, with solid catalysts offering enhanced structural stability and simpler separation processes that render them attractive for industrial applications. Meanwhile, the diverse structures, distinct electronic states, and facile functionalization of solid materials facilitate more flexible construction of customized FLP active sites and a broad spectrum of applications.^[^ ^33^ ^]^ Typically, metal‐based FLP provide superior activity and selectivity, thanks to the tunability of metal centers which can be precisely optimized for specific reactions. However, these catalysts are often costly, dependent on scarce or precious metals, and prone to poisoning or deactivation under certain conditions. In contrast, metal‐free FLP offer a more sustainable and economical alternative by eliminating the need for scarce metals and generally demonstrating enhanced resistance to poisoning, thereby improving their operational longevity. A potential limitation of metal‐free FLP, however, is that they may exhibit lower activity or selectivity in some cases. The aforementioned points will be discussed in comprehensive detail in the main body of the text. So far, FLP have been successfully explored in different heterogeneous solids, such as metal oxides,^[^ ^33^, ^34^, ^35^, ^36^, ^37^ ^]^ doped graphene,^[^ ^38^, ^39^, ^40^ ^]^ metal‐organic frameworks,^[^ ^41^, ^42^, ^43^, ^44^, ^45^ ^]^ zeolites,^[^ ^46^, ^47^, ^48^, ^49^, ^50^ ^]^ and other supporting materials.^[^ ^51^, ^52^, ^53^ ^]^ They have been applied in various thermal, electronic, and photonic reactions.

There are some recent reviews exploring the FLP chemistry in heterogeneous catalysis, including the design of CeO_2_‐based FLP,^[^ ^54^ ^]^ activation of CO_2_ by solid FLP,^[^ ^55^ ^]^ and the design of porous FLP.^[^ ^30^ ^]^ Our group has also been actively contributing to this field for several years, exploring its advanced structural characterization and H_2_ activation application.^[^ ^56^ ^]^ Despite these valuable contributions, a comprehensive elaboration of the synthesis, characterization, and application of FLP active sites in heterogeneous catalysis remains underexplored. This review addresses this gap by focusing on the three key areas. First, we outline strategies for constructing FLP sites on solid materials, emphasizing innovative approaches and design principles. Next, we critically evaluate advanced characterization techniques for identifying FLP active sites and probing reaction mechanisms on solid supports. Finally, we explore the applications of heterogeneous FLP in thermal, electrochemical, and photochemical small‐molecule activation, highlighting representative examples that disentangle the synergistic effects of FLP catalysts. We conclude by discussing emerging challenges and opportunities in heterogeneous FLP catalysis research.

## 2 Formation of FLP in the Solid Materials

Constructing FLP sites on solid materials is a critical research focus, requiring careful consideration of surface geometry, chemical composition, and electronic structure. This section explores strategies for generating FLP sites on solid surfaces, highlighting innovative approaches to synthesizing FLP catalysts with tailored reactivity and enhanced performance.

### 2.1 Strategies for FLP Construction

**Figure 3:**
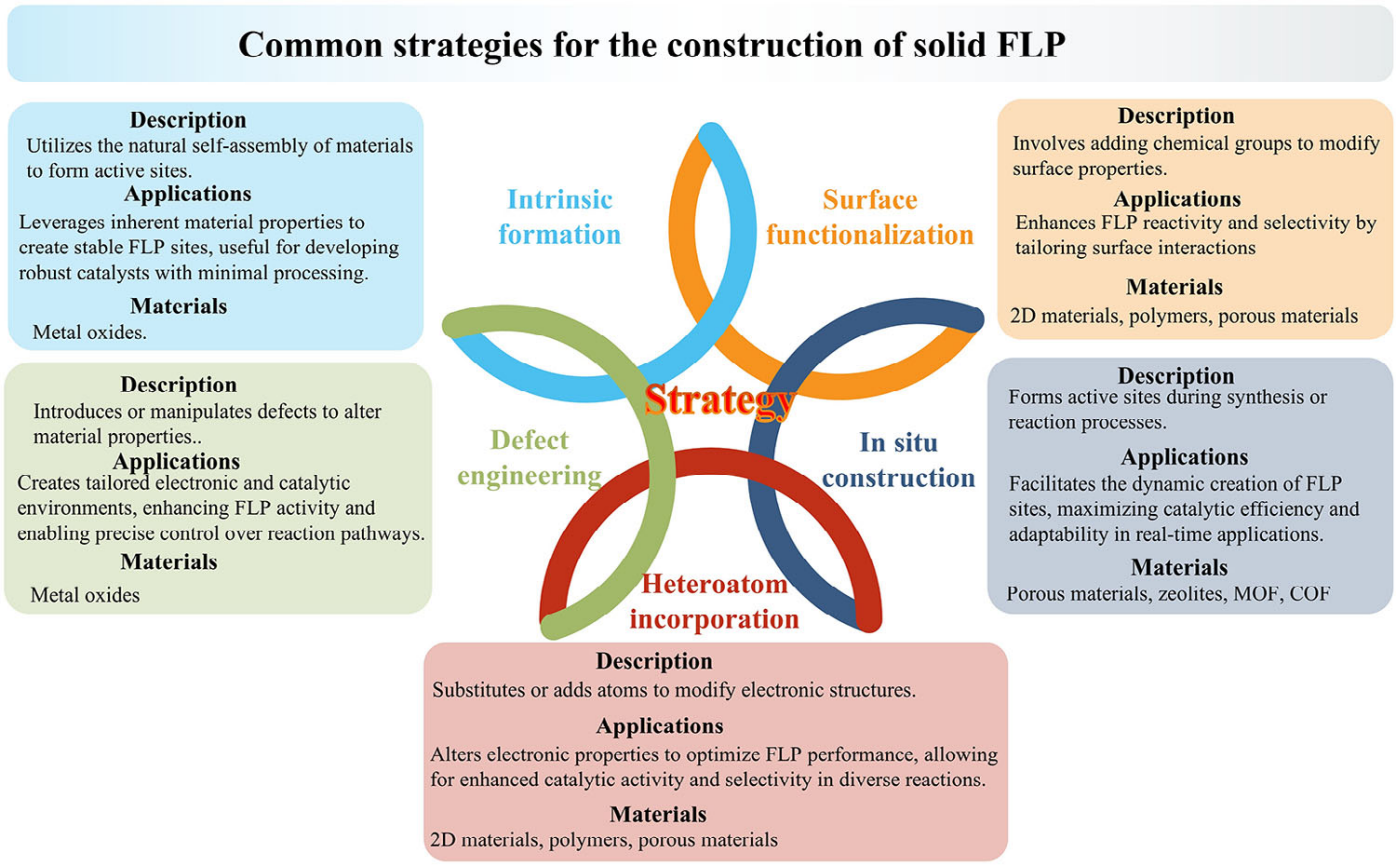
Summary of different strategies for the construction of FLP over solid materials.

The diverse strategies discussed here not only expand the functional capabilities of solid materials but also provide a versatile platform for the development of next‐generation catalytic systems with enhanced catalytic performances. Each subsection is expanded on the construction of different FLP systems by intrinsic formation, heteroatoms, and functional group incorporation, in situ induction, and other novel approaches summarized in **Figure** **3**.

#### 2.1.1 Intrinsic FLP Active Sites on the Surface of Metal Oxide

Metal oxides are widely adopted catalysts and catalyst supports due to their adjustable structures and surface properties. In these systems, the reactivity of surface atoms is usually correlated with the coordination number of the nearest neighbor, and lower coordination numbers often lead to higher acidity (M^n+^ sites) or basicity (O^2−^ sites) resulting from electron distributions.^[^ ^57^ ^]^ Meanwhile, the modifiable bandgap nature and carrier mobility featured by metal oxides broaden their applications in conductors and semiconductors. Intrinsic FLP can naturally form on metal oxide surfaces through interactions between unquenched acidic M^n+^ and basic OH^−^ or O^2−^ sites. These FLP are often stabilized by structural defects, particularly oxygen vacancies introduced during synthesis (since perfect crystals are rare) or calcination (via gas pretreatment). Two primary systems are commonly observed: (1) the surface hydroxyl‐oxygen vacancy ([OH‐Ov]) system, with unsaturated metal sites and surface hydroxide sites acting as the LA and LB, respectively. And (2) the surface oxygen‐oxygen vacancy ([O‐Ov]) system, where coordinatively unsaturated metal sites and lattice oxygen function as the LA and LB, respectively. We will demonstrate these points with some typical examples in the following content. It also should be noted that multiple FLP types may exist in one system due to the complication of atomic arrangements in solid materials and the potential structure reconstruction.

**Figure 4:**
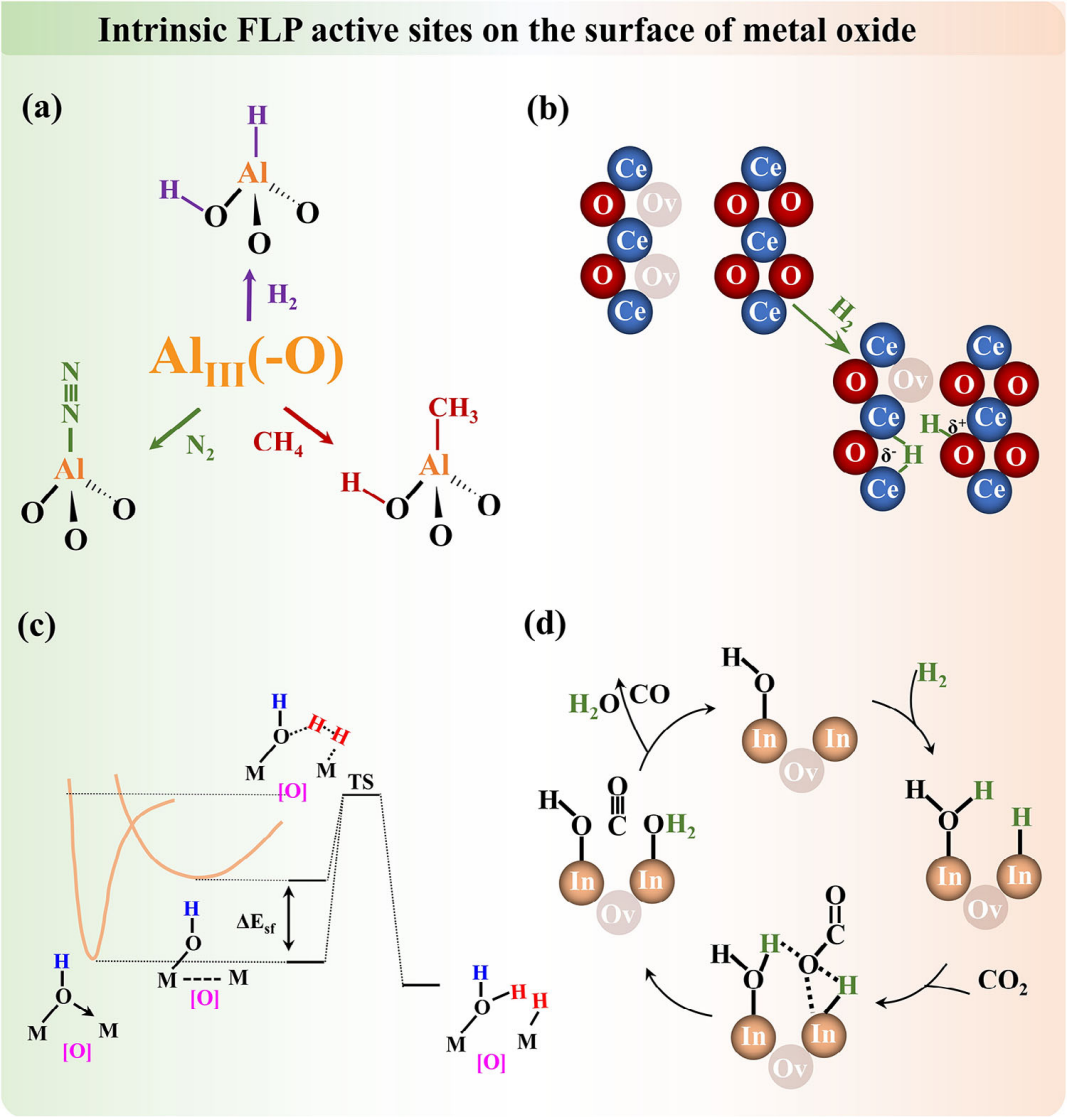
a) The surface natural FLP active sites on Al_2_O_3_ for small molecules activation. Adapted with permission.^[^ ^58^ ^]^ Copyright 2012, American Chemical Society. b) Oxygen vacancy on the surface of CeO_2_, working as the FLP active site for the activation of H_2_. Adapted with permission.^[^ ^59^ ^]^ Copyright 2018, American Chemical Society. c) Tuning the reactivity of surface FLP in M_2_O_3–x_OH_y_, where M = In and ΔE_sf_ = surface frustration energy advantage, which can be tuned through chemistry control of the Lewis acidity and Lewis basicity of surface metal M (acceptor) and surface hydroxide OH (donor) sites, respectively. Adapted with permission.^[^ ^60^ ^]^ Copyright 2016, American Chemical Society. d) Hydroxyl species on the surface of In_2_O_3_ and the FLP active sites for the activation of CO_2_. Adapted with permission.^[^ ^61^ ^]^ Copyright 2015, Royal Society of Chemistry.

γ‐Al_2_O_3_ represents one of the intrinsic FLP systems, where metastable three‐coordinate unsaturated Al^3^⁺ sites and non‐associated adjacent surface oxygen on (110) termination in a separation distance of 4.1 Å function as the most reactive LA–LB pairs from DFT calculation.^[^ ^34^, ^58^ ^]^ Pretreating alumina is compulsory to form FLP. Notably, a low water coverage on the surface facilitates both the stabilization of this (110) termination and the enhanced LA–LB activity for small molecule activation (**Figure** **4**a). Additionally, calcination of Aluminum‐containing precursors can generate FLP in defective Al₂O₃ structures. As pointed out by Yang et al.,^[^ ^62^ ^]^ the reactivity and selectivity of FLP active sites (i.e., the adsorption geometry of molecules) on Al₂O₃ are significantly influenced by the gas used during precursor calcination. Furthermore, Zheng et al.^[^ ^63^ ^]^ demonstrated that carboxylate‐containing Aluminum precursor will form a defect‐enriched Al₂O₃ catalyst, where surface pentacoordinate Al^3^⁺ and adjacent hydroxyl species function as FLP. In short, the oxygen vacancy sites on the Al₂O₃ surface are crucial for the formation and functionality of FLP.

Similarly, the intrinsic FLP are formed on the surface of cerium oxide (CeO₂), where unsaturated coordinated Ce^3^⁺ (LA) and adjacent lattice oxygen (LB) function as FLP active sites in a distance of 4 Å from computational simulation (Figure 4b).^[^ ^59^ ^]^ Due to the unique redox properties of CeO₂, which lead to its remarkable catalytic activity,^[^ ^64^ ^]^ Ce^3^⁺ and Ce⁴⁺ redox couples can undergo reversible switching, accompanied by oxygen storage and release above 150 °C. CeO₂ surfaces possess a large number of acid‐base sites, and the surface oxygen vacancies not only avoid the partial formation of classic LA–LB adjunct but also enhance both the acidity of FLP‐acid sites and the basicity of FLP‐base sites on CeO₂ surfaces.^[^ ^54^, ^59^, ^65^, ^66^ ^]^ A similar system is observed in titanium oxide.^[^ ^67^, ^68^, ^69^ ^]^ Additionally, researchers have identified many other natural FLP in different metal oxides belonging to the [O‐Ov] systems, such as MgO,^[^ ^70^ ^]^ Nb_2_O_5_,^[^ ^71^ ^]^ MnO_2_.^[^ ^72^ ^]^ Recently, Chang et al.^[^ ^37^, ^73^ ^]^ provided comprehensive simulations of natural surface FLP on stable wurtzite crystal surfaces. On the other hand, indium hydroxide (In(OH)_3_) is one of the typical [OH‐Ov] systems of FLP (Figure 4c,d). Calcination of synthesized (In(OH)_3_) in air induces the extraction of surface oxygen, creating vacancies that facilitate the formation of FLP.^[^ ^61^ ^]^ This process results in the formation of In₂O_3‐x_(OH)γ, a material characterized by spatially separated LB and LA sites, denoted as InOH···In, which constitute the FLP system.^[^ ^35^, ^36^, ^60^, ^61^ ^]^ At these oxygen vacancy sites, CO₂ molecules are activated through interaction with adjacent hydrogen adatoms following heterolytic cleavage. In contrast, the classical linked LB‐LA complex InOH→In, exhibits no activity due to the absence of oxygen vacancies and inaccessible active sites.^[^ ^35^, ^60^, ^74^ ^]^

Additionally, other materials, including 2D supports^[^ ^38^, ^75^, ^76^ ^]^ and 3D porous materials^[^ ^77^ ^]^ with natural acid and base sites, may also feature intrinsic FLP sites due to structural defects. Garcia et al.^[^ ^38^ ^]^ emphasized the importance of LA and LB sites in graphene and graphene oxides for hydrogenation reaction in the absence of metals. In their study, the gas environment during the reaction will vary the activities of these sites significantly. Inspired by B/N homogenous FLP, Dai et al. examined the synthesis and application of 2D hexagonal boron nitride (h‐BN)^[^ ^75^ ^]^ and BN‐enriched nanoporous networks.^[^ ^76^ ^]^ In both cases, defects and active sites are precisely controlled during synthesis, and the resulting material shows both acidity and basicity. The cleavage ability of synergistic sites was verified by DRIFT and DFT calculations. Defective metal‐organic framework (MOF) also have the potential to be FLP‐active. Two FLP active sites, Zr^4+^/O^2−^ and Zr^4+^/OH^−^, were suggested in UiO‐66 with insufficient organic linkers.^[^ ^77^ ^]^

#### 2.1.2 Modified FLP Active Sites by Heteroatom Doping

In addition to direct synthesis and calcination methods, which modify the surface property of materials by creating intrinsic vacancies for the formation of unquenched LA and LB sites, FLP entities can also be engineered by incorporating heteroatoms into the material. This approach modifies surface properties and enhances FLP activity through two primary methods: (1) Element substitution, in which heteroatom replaces lattice atoms of the support. (2) Element impregnation, in which heteroatom sits on the surface and/or defective sites (e.g., interstitial sites and vacancies) of the support. Both ways, with dopants across the periodic table, could successfully alter the coordination environments and charge distributions. Thereby, dopants often increase the concentration and activities of existing FLP types by promoting vacancy formation and/or introducing new combinations of FLP active sites.

Substituting elements into supports is a common structure modification method that can be found in metal oxides, carbon supports, and other 2D materials. Among all, CeO_2_ is an experimentally and computationally well‐studied example with FLP activity. The substitution of cerium atoms by Ni,^[^ ^78^, ^79^, ^80^ ^]^ Gd,^[^ ^81^ ^]^ Pt,^[^ ^82^ ^]^ La,^[^ ^83^ ^]^ and other heavy metals have been reported. Due to discrepancies between atomic radii, the ceria lattice often experiences distortion, which varies the distances between active LA Ce atoms and LB O atoms. Meanwhile, dopants often modify the Bader charge distributions while creating more charge‐balancing oxygen vacancies. Ce/O FLP with improved acid‐base activity is populated, with potential additional M/O pairs, for improved activation ability for small molecules. Doping indium oxide (In₂O₃) with non‐metal elements such as nitrogen, carbon, and sulfur can enhance light absorption, carrier separation, and CO₂ adsorption, thereby improving photocatalytic activity. Nitrogen‐doped In₂O₃ (N‐In₂O₃) exhibited high performance and stability for methanol formation from photocatalytic CO₂ reduction in aqueous solution under ambient conditions.^[^ ^84^ ^]^ Furthermore, the introduced P‐block dopants (i.e., B, C, N, P), which replace the outermost O atoms and their adjacent non‐bonding zinc site on Wurtzite structure ZnO (100), were identified as the FLP.^[^ ^85^ ^]^ The distances between these dopants and Zn were calculated to range from 3.35 to 3.78 Å, allowing concerted adsorption and activation of molecules.

**Figure 5:**
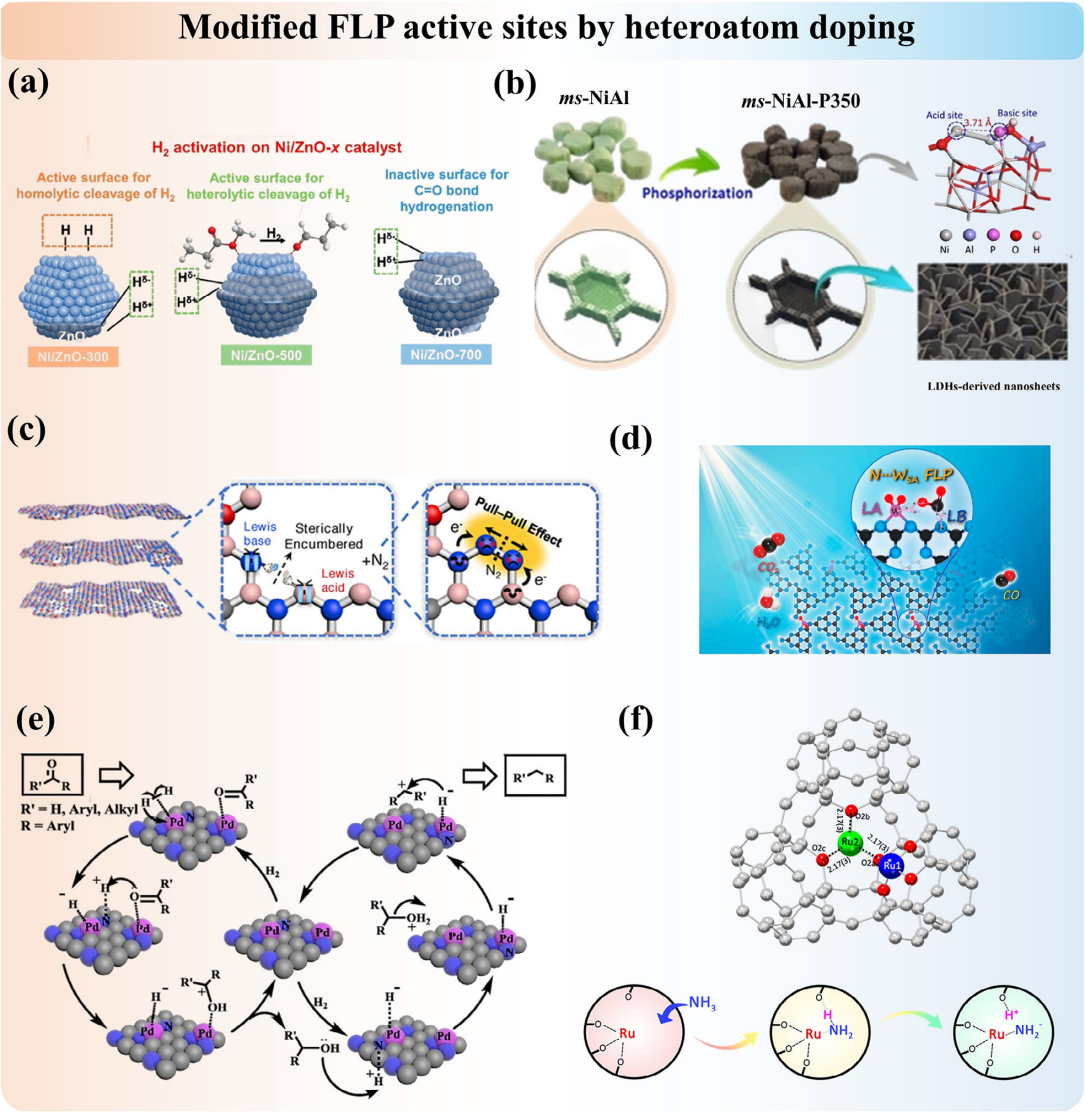
a) Ni‐ZnO interface with the formation of FLP active site. Adapted with permission.^[^ ^86^ ^]^ Copyright 2023, American Chemical Society. b) Ni(‐O‐Al)·P FLP in P‐doped NiAl oxide. Reproduced under the terms of the CC‐BY Creative Commons Attribution 4.0 International license (https://creativecommons.org/licenses/by/4.0).^[^ ^87^ ^]^ Copyright 2024, The Authors, published by Springer Nature. c) FLP active site on defective boron carbon nitride. Adapted with permission.^[^ ^88^ ^]^ Copyright 2022, Wiley‐VCH. d) Introduced tungsten single‐atoms into polymeric carbon nitride to form FLP. Adapted with permission.^[^ ^89^ ^]^ Copyright 2025, American Chemical Society. e) Nitrogen‐doped carbon supported Pd catalyst. Adapted with permission.^[^ ^90^ ^]^ Copyright 2018, Elsevier. f) Incorporation of single Ru atom inside 13X zeolite to build FLP for NH_3_ decomposition. Reproduced under the terms of the CC‐BY Creative Commons Attribution 4.0 International license (https://creativecommons.org/licenses/by/4.0).^[^ ^48^ ^]^ Copyright 2023, The Authors, published by American Chemical Society.

The heteroatoms can also be incorporated on the surface and/or at defects (e.g., interstitial sites, vacancies) of 2D supports such as metal oxides as well as 3D porous systems. This often stimulates the possibilities of new FLP systems and new additional active mechanisms (i.e., dual active sites). The partially coated Ni/ZnO calcined at 500 °C exhibits typical characteristics of FLP (**Figure** **5**a). The nickel cluster functions as the LA, and electron‐enriched oxygen sites in defective ZnO act as the LB, facilitating the heterolytic cleavage of H₂ at the Ni‐ZnO interface.^[^ ^86^ ^]^ A similar FLP formed between acidic metal dopants and basic support was also reported in Ru/MgO,^[^ ^91^, ^92^ ^]^ Au/hydrotalcite,^[^ ^93^, ^94^ ^]^ and Ni─CeO_2_/SiO_2_.^[^ ^95^ ^]^ More recently, Wang et al. reported a robust phosphorus‐doped NiAl‐oxide catalyst featuring FLP, where the phosphorus atom bonded with an oxygen atom acts as an electron donor, and the spatially separated nickel atom serves as an electron acceptor (Figure 5b). This configuration enables efficient green diesel production without the need for sulfur replenishment.^[^ ^87^ ^]^

Several carbon‐based supports and porous materials were also investigated. Fu et al.^[^ ^88^ ^]^ presented a molecular design strategy to develop a defective boron carbon nitride (BCN) catalyst (Figure 5c), utilizing abundant unsaturated boron and nitrogen atoms as LA and LB sites. This approach upgrades the catalyst from a single “Lewis acid catalyst” to “FLP catalyst”. Yang et al.^[^ ^89^ ^]^ introduced tungsten single atoms into polymeric carbon nitride to prepare an atomic tungsten‐based solid FLP catalyst (Figure 5d). In this material, the electron‐deficient tungsten single‐atom serves as LA, while the adjacent electron‐rich nitrogen atom functions as LB. Other metal‐doped carbon nitrides (C_2_N) with metal/N FLP systems were analyzed by Wang et al.^[^ ^96^ ^]^ for hydrogen activation and hydrogenation. Pd‐incorporated nitrogen‐doped carbon also features FLP activity between Pd (LA) and nitrogen (LB).^[^ ^90^ ^]^ In the context of zeolites, the incorporated metal ion and the framework oxygen can function as LA and LB, respectively.^[^ ^97^ ^]^ Recently, our group demonstrated this by introducing ruthenium into 13X zeolite, a single confined Ru atom can form a Ru^δ+^/O^δ−^ FLP. This configuration exhibits superior activity compared to most ruthenium‐containing catalysts for NH₃ cracking.^[^ ^48^ ^]^

#### 2.1.3 Modified FLP Sites by Functional Group Immobilization

**Figure 6:**
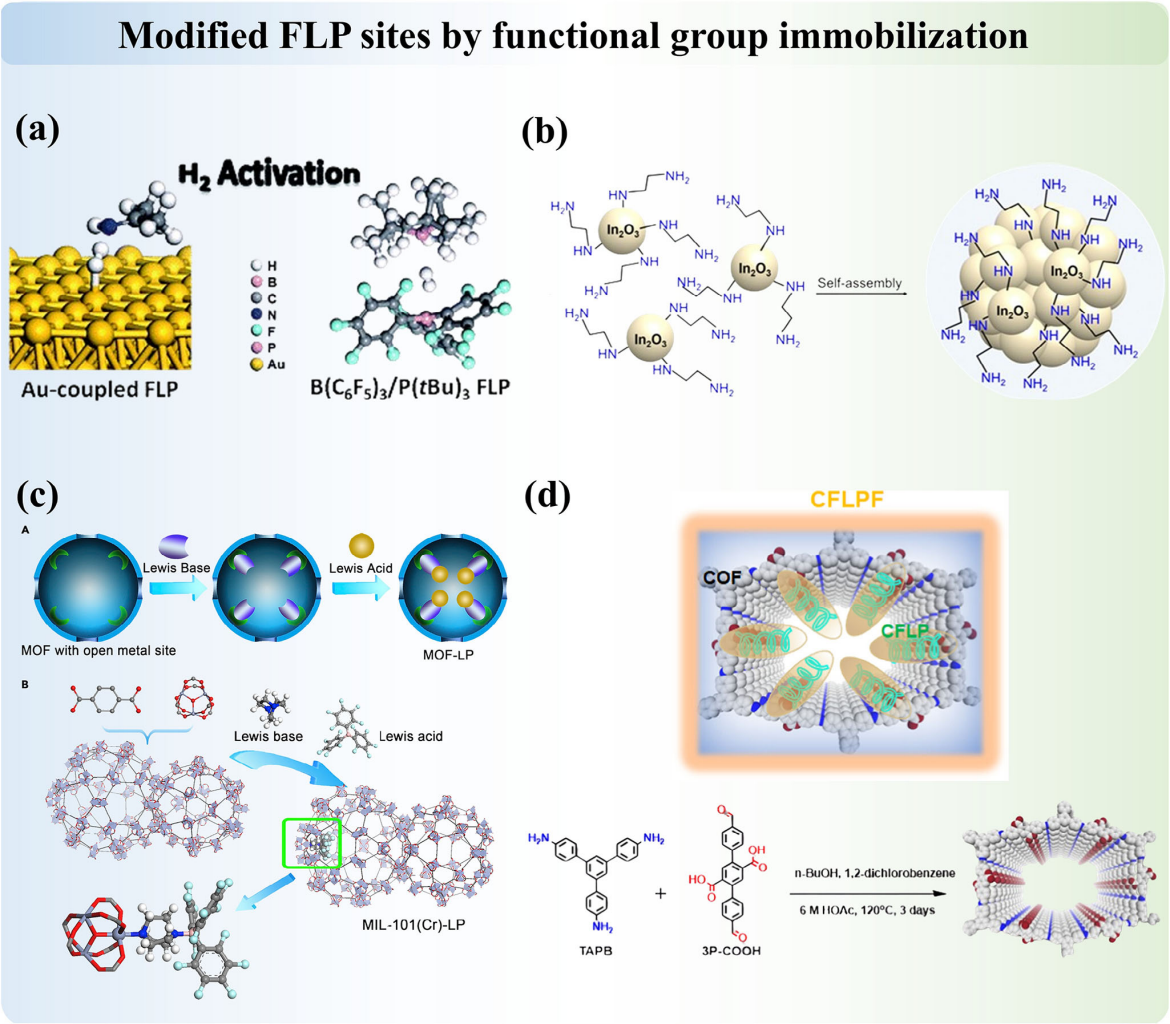
a) The Lewis base‐coupled Au FLP. Adapted with permission.^[^ ^98^ ^]^ Copyright 2014, Royal Society of Chemistry. b) Construction of FLP on In_2_O_3_ nanocrystals with amine‐functionalization. Reproduced under the terms of the CC‐BY Creative Commons Attribution 3.0 Unported license (https://creativecommons.org/licenses/by/3.0).^[^ ^102^ ^]^ Copyright 2024, The Authors, published by Royal Society of Chemistry. c) Construction of FLP into MOF. Adapted with permission.^[^ ^103^ ^]^ Copyright 2018, Elsevier. d) Construction of chair FLP into COF. Adapted with permission.^[^ ^108^ ^]^ Copyright 2024, American Chemical Society.

Motivated by both homogeneous catalysts and heterogeneous catalysts, surface modification by immobilizing tailored functional groups also allows precise tuning of chemical environments, which optimizes molecular‐level interactions for specific reactions. The flexibility in both the chemical and geometrical properties of active molecular sites allows for improved reaction efficiency and product selectivity. Introducing molecules on inert surfaces, including gold^[^ ^98^, ^99^ ^]^ and silica^[^ ^100^, ^101^ ^]^ was first studied and reported (**Figure** **6**a). Later on, amine functionalization of various photocatalysts (such as oxides, sulfides, and graphitic carbon nitride), which enables substantial CO_2_ adsorption through acid‐base interactions, has been summarized by Guan et al.^[^ ^102^ ^]^ (Figure 6b). On the other hand, MOF materials have recently emerged as a novel class of heterogeneous catalysts, offering tunable structures and customizable pore environments that present new opportunities for selective catalysis. An experimental breakthrough was achieved by Ma's group^[^ ^103^ ^]^ in 2018, who successfully introduced molecular FLP into MOF through a stepwise synthesis approach, with excellent recyclability (Figure 6c). Numerous studies on heterogeneous FLP MOF catalysts have since been reported.^[^ ^44^, ^45^, ^104^, ^105^, ^106^ ^]^ In addition to MOF, covalent organic frameworks (COF) have also been developed as supports for the construction of FLP active sites. The first COF‐FLP was reported by Hu et al.^[^ ^107^ ^]^ in 2021, demonstrating that triaryl phosphorus, serving as LB, could be anchored in two types of bromine‐functionalized COF. Subsequently, tris(pentafluorophenyl)boron (BCF) was introduced as the LA, resulting in COF‐supported heterogeneous FLP catalysts. These catalysts exhibit excellent catalytic reactivity for the hydrogenation of alkynes to olefins. More recently, Ma et al.^[^ ^108^ ^]^ reported the introduction of bulky chiral LB into COF, followed by the incorporation of an LA such as tris(pentafluorophenyl)borane (Figure 6d). This configuration, featuring achiral LA with chiral LB within the COF, demonstrates superior asymmetric hydrogenation capabilities. The introduction of foreign organic species to design new FLP increases the reactivity of metal surfaces, providing a valuable method for designing additional metal‐based hydrogenation catalysts.

#### 2.1.4 In Situ Formation of Induced FLP Active Sites

The most fascinating aspect of heterogeneous catalysis is that, during the reaction process, the catalyst's properties, including its structure and electronic characteristics, continuously change, resulting in more mechanistic possibilities. In contrast to FLP sites which are engineered into the material's surface or pores prior to reactions, in situ FLP sites could arise dynamically under operational conditions, which are often triggered by specific catalytic environments. Critical processes such as adsorption, light illumination, and thermal treatment play pivotal roles in this spontaneous formation. All three stimuli will activate sites for FLP chemistry by facilitating structural flexibility, atomic rearrangements, and/or electron redistribution. This capability to generate FLP active sites in situ not only enhances the versatility and responsiveness of catalytic systems but also provides deeper insights into the mechanistic aspects of FLP chemistry, paving the way for novel applications and improved catalytic processes.

**Figure 7:**
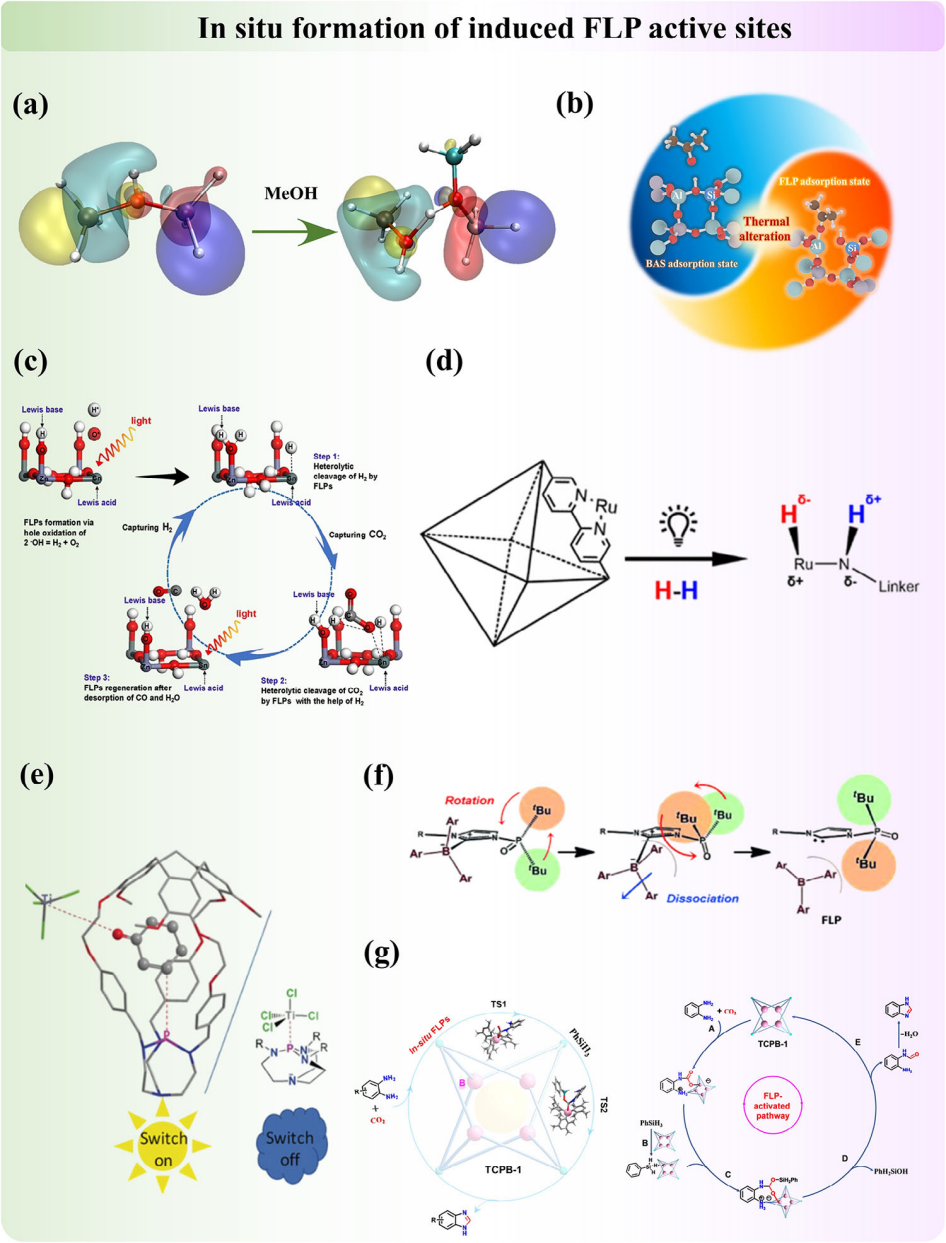
a) Methanol adsorption induced FLP from BAS in SAPO34 zeolite. Adapted with permission.^[^ ^47^ ^]^ Copyright 2021, American Chemical Society. b) Thermal induced the transformation of BAS to FLP in SAPO34 zeolite. Reproduced under the terms of the CC‐BY Creative Commons Attribution 4.0 International license (https://creativecommons.org/licenses/by/4.0).^[^ ^49^ ^]^ Copyright 2022, The Authors, published by Wiley‐VCH. c) Photo‐induced surface hole oxidation as the FLP active site on ZnSn(OH)_6_. Adapted with permission.^[^ ^109^ ^]^ Copyright 2021, Elsevier. d) Photo‐induced FLP active site in Ru‐MOF catalyst. Reproduced under the terms of the CC‐BY Creative Commons Attribution 4.0 International license (https://creativecommons.org/licenses/by/4.0).^[^ ^110^ ^]^ Copyright 2023, The Authors, published by American Chemical Society. e) Light‐induced bulky substituents molecular coordination changes for the formation of FLP. Adapted with permission.^[^ ^111^ ^]^ Copyright 2018, Wiley‐VCH. f) Rotation of the bulky group for the formation of FLP from the classic LA–LB adduct. Rotation‐activated FLP. Adapted with permission.^[^ ^112^ ^]^ Copyright 2015, Wiley‐VCH. g) In‐situ creation of FLP in MOF with the introduction of LB. Adapted with permission.^[^ ^113^ ^]^ Copyright 2023, Chinese Chemical Society.

Gas adsorption ability, which often involves electron transfer in chemical adsorption, is directly related to the catalytic performance and product selectivity. Zeolites that have outstanding gas adsorption and separation ability serve as essential heterogeneous catalysts in a wide range of industrial reactions, yet the creation and precise location of active sites for molecular adsorption and catalysis remain subjects of ongoing debate. Our recent research demonstrates that in silica‐aluminophosphate zeolites (SAPO), conventional Brønsted acid sites (BAS) can transform into FLP through the cleavage of the Al‐O bond upon adsorption of small polar molecules (**Figure** **7**a).^[^ ^47^ ^]^ This transformation results in the formation of a three‐coordinated framework aluminum as the LA and SiO(H) as the LB. A strategy has been developed to activate FLP from CLA by leveraging molecular motions that respond to external stimuli. In our related work, we also demonstrated that thermal treatment can significantly assist the formation of synergistic sites (Figure 7b).^[^ ^49^ ^]^ The increase in the adsorption temperature facilitated the transformation of BAS into induced FLP on SAPO‐34 zeolite. In similar, Breher et al.^[^ ^114^ ^]^ reported that the concealed aluminum–carbon FLP catalyst, where the LA/LB adduct manifests as a quenched FLP in the solid state, can engage in a thermoneutral reaction with non‐aqueous ammonia, reversibly cleaving the N─H bond at ambient temperature.

Light irradiation is another stimulus that promotes electrons between energy bands, resulting in changes in electronic properties. When a single‐crystal ZnSn(OH)₆ cube with a (100) surface composed of alternating Zn−OH and Sn−OH terminal hydroxyl groups is irradiated under a Xe lamp, Ov are generated as LA by oxidizing the light‐unstable terminal hydroxyls at Sn sites (Figure 7c).^[^ ^109^ ^]^ These photo‐induced O_v_s form FLP with adjacent light‐stable Zn−OH terminal hydroxyls, which act as LB. This FLP interaction significantly reduces the energy barriers for capturing, activating, and reducing CO₂ into CO with the assistance of H₂. Besides, our group recently discovered that ruthenium MOF‐based catalysts, synthesized using nitrogen‐containing linkers, are initially catalytically inactive for H₂ activation, even at elevated temperatures (Figure 7d).^[^ ^110^ ^]^ However, upon exposure to light, a metal‐to‐ligand charge transfer occurs, inducing charge polarization in the anchored Ru bipyridine complex. This process forms a transient LA–LB pair in Ru⁺−N⁻ bond, which facilitates the reaction. In a related study, Martinez et al.^[^ ^111^ ^]^ reported a light‐induced FLP system achieved by confining the LB partner within a molecular cavity. While the model superbase without the cavity exhibited no catalytic activity, its association with titanium (IV) chloride transformed the encaged superbase into an efficient catalyst under identical conditions (Figure 7e).

As mentioned briefly in the case of SAPO above, heat is an energy source that thermodynamically favors the sites toward FLP‐type. In 2015, by utilizing the rotation flexibility of the phosphine oxide moiety on substituted imidazolylidenes at above 60 °C, Ogoshi et al.^[^ ^112^ ^]^ reactivated FLP from self‐stable thermodynamic CLA carbene‐borane complexes at room temperature (Figure 7f). This reversible approach allows for the dynamic control of FLP activity, so FLP sites can be reactivated from CLA when needed. Later on, they introduced another thermally induced revival of FLP from the self‐stable adducts.^[^ ^115^ ^]^ This process involves a thermally induced borane transfer from the carbene carbon atom to the N‐phosphinoyl oxygen atom, initiating the transformation of the carbene–borane adduct. Subsequent conformational isomerization, through the rotation of the N‐phosphinoyl group in Poxim moieties, ultimately revives the frustrated carbene–borane pairs capable of cleaving H₂. FLP formed by continuously introducing molecular substances could also fall in this classification of “in situ formation of FLP”. Ma et al.^[^ ^113^ ^]^ synthesized a robust metal‐organic cage material featuring a bulky Lewis acid functionalized linker, where FLP active sites are formed in situ upon the addition of Lewis basic substrates (Figure 7g). Similarly, Shi et al.^[^ ^116^ ^]^ reported the in situ creation of FLP active sites in a MOF by using acetic acid as a modulator to induce missing ligands in UiO‐66 (Zr), resulting in the formation of Zr^3^⁺─OH FLP at the defective sites.

#### 2.1.5 Other Strategies for the Creation of the Tailored FLP Active Site

Beyond the established methods of post‐synthesis calcination, heteroatom doping, functional group modification, and in situ generation, a variety of emerging strategies and considerations are being explored to effectively tailor FLP active sites for target reactions. Techniques such as nanostructuring, the use of novel support materials, and the incorporation of external stimuli‐responsive components are also gaining traction. In the following subsection, we will further present the factors impacting FLP generation and the amazing operational flexibility of FLP chemistry. All of these innovative approaches expand the toolkit available for FLP design by flexibly utilizing both chemical and geometrical/structural properties of heterogeneous catalysts. Furthermore, they enable fine‐tuning of catalytic behavior in response to specific reaction conditions, paving the way for more efficient and versatile catalytic systems.

**Figure 8:**
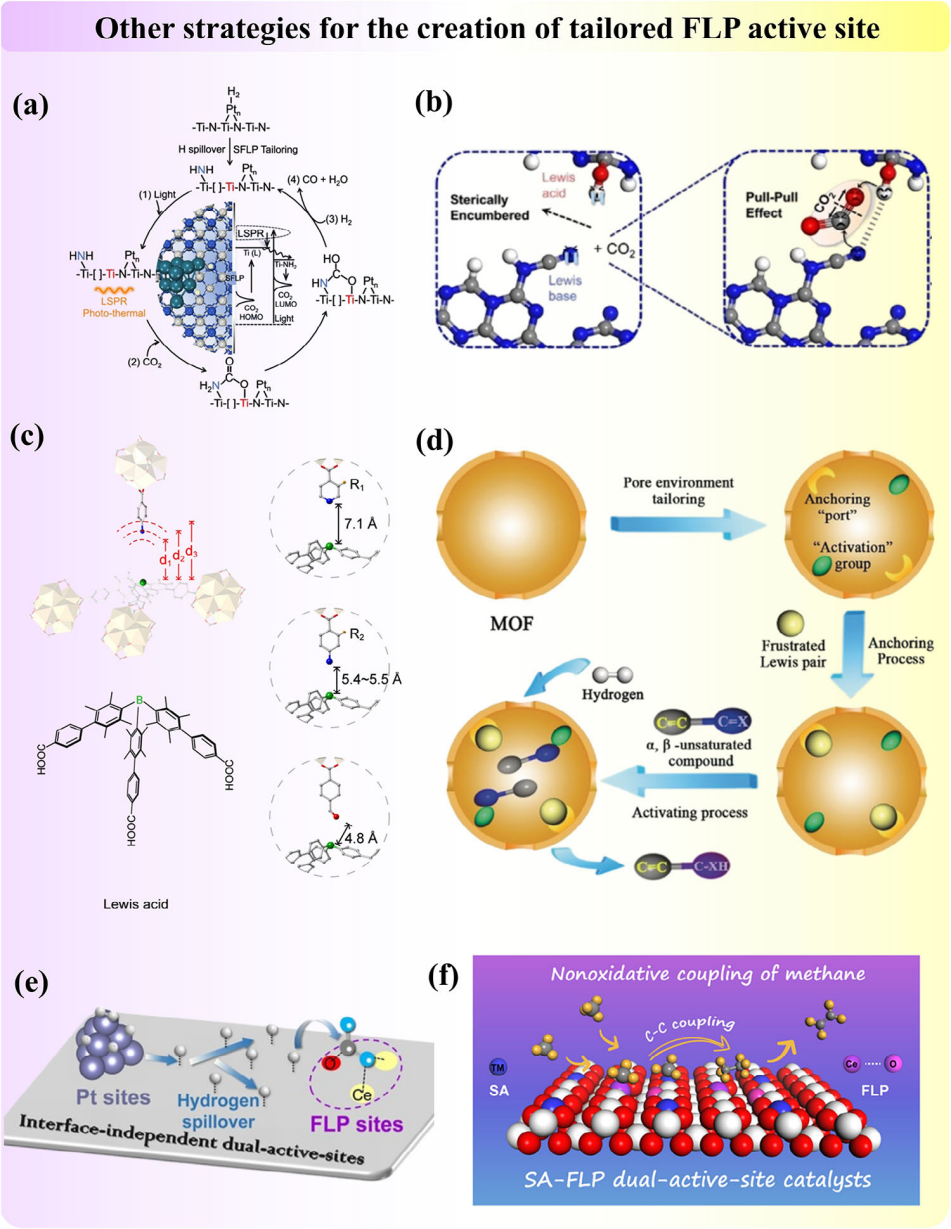
a) The hydrogen spillover‐induced Ti‐NH_2_ and adjacent Ti(L) atoms form an amide‐type surface FLP. Adapted with permission.^[^ ^117^ ^]^ Copyright 2024, Springer Nature. b) Construction of FLP in Poly(heptazine Imide) nanosheets via hydrogen bonds. Adapted with permission.^[^ ^118^ ^]^ Copyright 2024, Wiley‐VCH. c) Construction of various FLP active sites in MOF by adjusting the distance between LA and LB. Adapted with permission.^[^ ^119^ ^]^ Copyright 2024, American Chemical Society. d) Anchor the FLP active site on MOF material. Adapted with permission.^[^ ^45^ ^]^ Copyright 2019, Wiley‐VCH. e) The creation of dual active sites with FLP and metal cluster for the synergy effect. Adapted with permission.^[^ ^121^ ^]^ Copyright 2023, Wiley‐VCH. f) Creation of dual active sites with the single atom and FLP sites. Adapted with permission.^[^ ^82^ ^]^ Copyright 2023, American Chemical Society.

Modifying the structures of metal oxides via metal incorporation could also generate FLP in a different mechanism from Section 2.1.2. Ling et al.^[^ ^117^ ^]^ reported a case that combines the concept of both modified FLP and induced FLP. In platinum‐doped nitrogen‐substituted titanium oxides (Pt/TiN_x_O_y_) for light‐assisted reverse water‐gas shift (RWGS) reaction, the FLP was formed between low‐valent Ti (LA) and Ti‐NH_2_ surface species (LB), which were operando generated by the hydrogen spillover ability of nano‐scale co‐catalyst platinum (**Figure** **8**a). Xiong et al.^[^ ^118^ ^]^ proposed another intriguing strategy which leveraged hydrogen bonding as a “steric hindrance” to facilitate the spatial separation between nitrogen and hydrogen atoms to construct FLP in poly (heptazine imide). Through theoretical calculations, they confirmed that the unquenched H atoms (LA) and N atoms (LB) exhibit an enhanced effect of hydrogen bonding‐mediated FLP on CO₂ adsorption energy, thereby contributing to CO₂ trapping and polarization in the active region (Figure 8b).

Careful structure modification in MOF before introducing molecular FLP species could also smooth the way for FLP chemistry by achieving ideal FLP separation distances (spatial constraints). Deng et al.^[^ ^119^ ^]^ have illustrated that tailoring anchor sites for active organics allows precise control over the spatial arrangement between the LA and LB. In their examples of modified MOF, a series of FLP with fixed distances was constructed by strategically anchoring the tritopic organoboron linker (LA) and the monotopic LB linkers (LB) to the separate metal oxide clusters (Figure 8c). These spatial constraints on active sites imposed by immobilization of molecular catalysts to enhance the intrinsic activity of bifunctional catalysts were emphasized by Corminboeuf et al.^[^ ^120^ ^]^ Additionally, the pore environment of MOF can also be tailored to populate the active sites. Ma et al.^[^ ^45^ ^]^ reported that post‐synthesis dehydration allows for the precise exposure of creating “ports” specifically designed for anchoring FLP and activation groups, which preferentially interact with targeted double bonds in substrates, thereby enabling selective reduction during hydrogenation (Figure 8d). In this process, the FLP plays a crucial role in facilitating H₂ activation, while the activation groups stabilize the reactants.

Simultaneously, the development of FLP‐related dual‐active sites on solid materials has garnered significant attention due to its potential to unlock novel reaction pathways and mechanisms that single‐site catalysts cannot access. This dual‐site approach enhances reaction rates and selectivity by optimizing the proximity and interaction between the active sites, thereby enabling more efficient substrate activation and transformation with its flexible structure design. It also paves the way for the development of multifunctional catalysts capable of executing sequential or tandem reactions in a single step, thereby increasing process efficiency and minimizing the need for additional reagents or purification steps.

Qu et al.^[^ ^121^ ^]^ reported the development of a dual‐active site catalyst by integrating platinum clusters with FLP sites on ceria oxide (Figure 8e). This catalyst exhibited remarkable reactivity for the RWGS reaction at low temperatures. The synergy between the active sites was clearly demonstrated, with H₂ dissociation occurring on the platinum sites and CO₂ activation on the FLP sites. Chang et al.^[^ ^82^ ^]^ proposed a novel approach for constructing “Single‐Atom”‐“Frustrated Lewis Pair” (SA‐FLP) dual‐active‐site catalysts on CeO_2_. Their method involves creating a single‐atom site by doping a platinum atom at the cerium site on the CeO_2_ surface (Figure 8f). Concurrently, the FLP site is fabricated by strategically removing oxygen atoms adjacent to the platinum atoms. This design of SA‐FLP sites enables the simultaneous activation of two methane molecules, significantly enhancing the C─C coupling of hydrocarbon species to generate C2 products. Expanding on this strategy, they further explored the doping of various other transition‐metal atoms, such as Fe, Co, Ni, Cu, Ru, Rh, Pd, Ag, Os, Ir, Pt, and Au, into the CeO_2_(110) surface to generate SA‐FLP.^[^ ^122^ ^]^ Their modelling results demonstrated that introducing single atoms into the CeO_2_ surface facilitates both the formation of oxygen vacancies for the construction of FLP sites and the dual active sites.

### 2.2 Outlook of FLP Design

**Figure 9:**
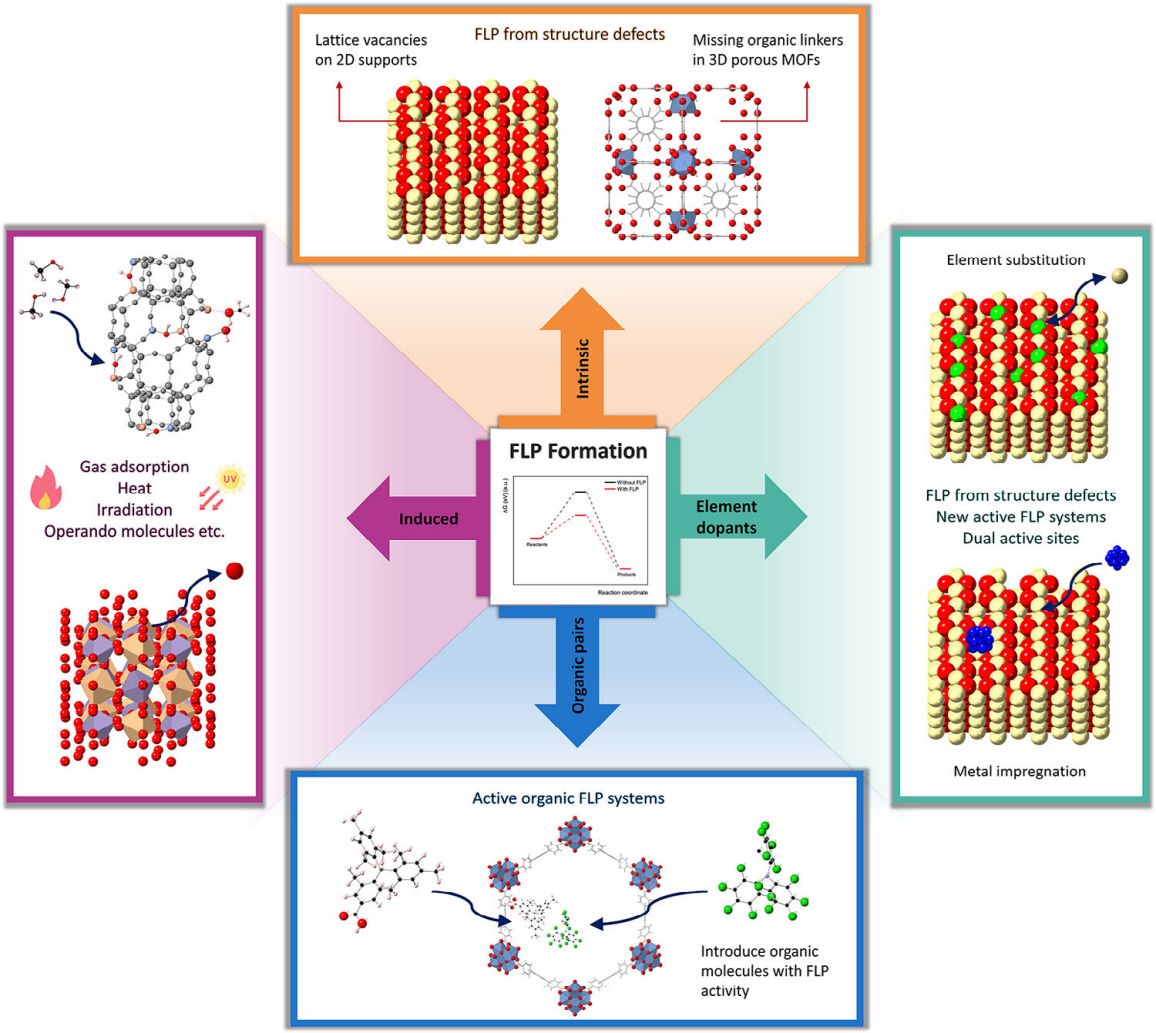
Summary of heterogeneous FLP construction strategies.

FLP active sites are often synergistic systems composed of electron‐poor LA sites and electron‐rich LB sites. The design of novel FLP catalysts, which fundamentally arise from charge differences, offers significant strategic flexibility, as outlined in Section 2.1, thereby facilitating the development of intrinsic and modified heterogeneous FLP systems (**Figure** **9**). Moreover, the dynamic in situ FLP systems formed during reactions deserve further exploration to fully exploit their catalytic potential. To further advance catalyst design for potential industrial applications, it is essential to assess and tune the properties of existing heterogeneous catalysts according to the structure‐activity relationship, which links catalytic activity with inherent catalyst‐adsorbate physicochemical properties.^[^ ^123^ ^]^ It shares commonality with homogeneous systems summarized by Paradise,^[^ ^124^ ^]^ of which steric and electronic properties matter significantly to catalytic performances and are therefore worth controlling. To understand this relationship which involves multivariable, both experimental characterization and computational modelling are compulsory.

**Figure 10:**
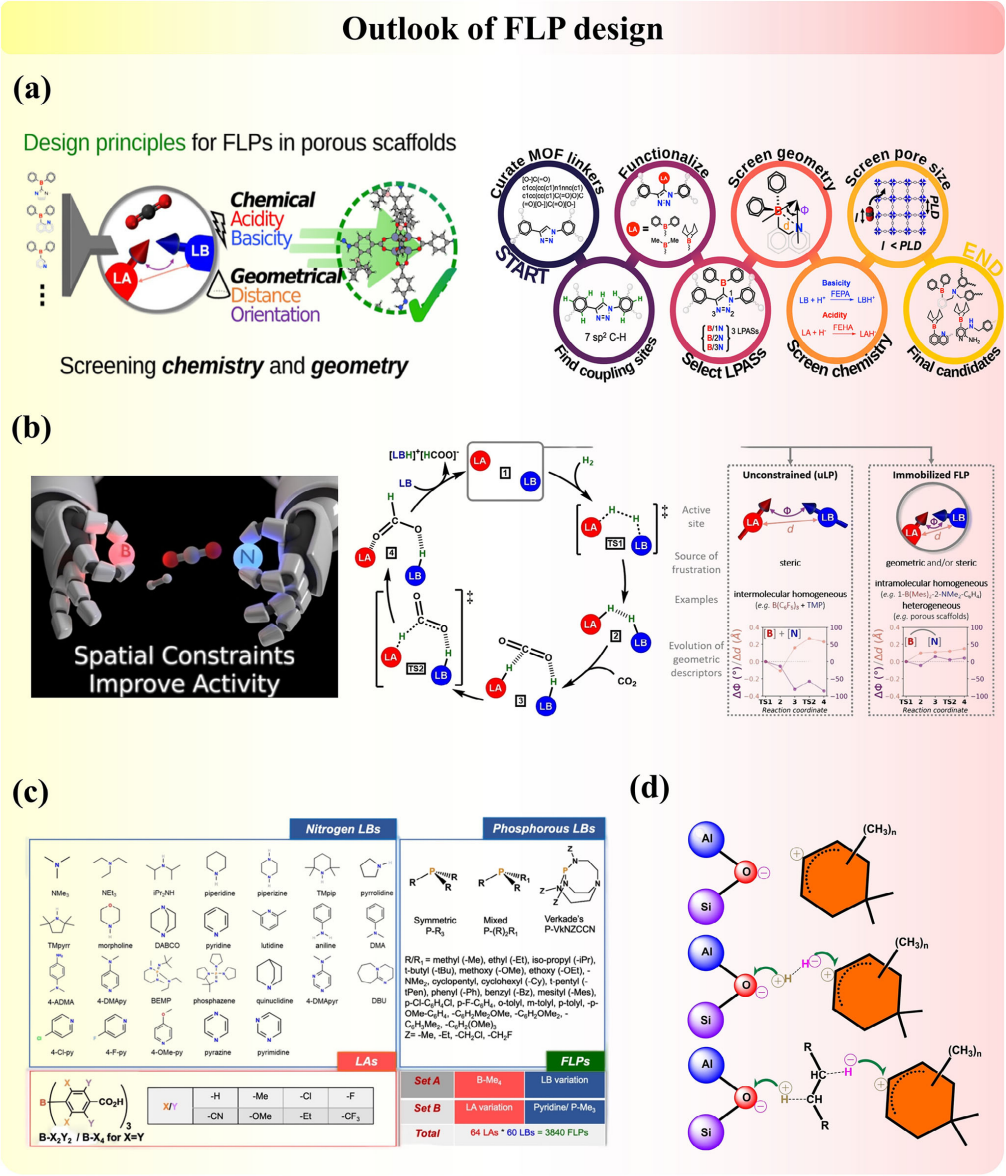
a) Design principle of FLP in the porous materials related to the screening of chemical and geometry environments. Reproduced under the terms of the CC‐BY Creative Commons Attribution 4.0 International license (https://creativecommons.org/licenses/by/4.0).^[^ ^125^ ^]^ Copyright 2024, The Authors, published by American Chemical Society. b) Spatial constraints on the design of efficient FLP catalysts. Adapted with permission.^[^ ^120^ ^]^ Copyright 2022, Wiley‐VCH. c) Screening of the various LBs for the construction of FLP active sites. Adapted with permission.^[^ ^126^ ^]^ Copyright 2022, Wiley‐VCH. d) in situ formed FLP in zeolite with carbenium ion (LA) and the deprotonated BAS. Adapted with permission.^[^ ^50^ ^]^ Copyright 2022, Wiley‐VCH.

Brønsted–Evans–Polanyi (BEP) and other linear relationships could computationally describe the reactivity trends of frustrated LA–LB combinations. They explore key structure factors such as acidity of LA, basicity of LB, chemical hardness, and softness of the FLP sites.^[^ ^55^ ^]^ With more structural factors to be considered, Corminboeuf et al.^[^ ^120^, ^125^, ^126^ ^]^ advanced the computational model with non‐linear relationships and mapped the active site geometry and chemistry to the activity of MOF in photocatalytic CO₂ hydrogenation. Building upon the insights from Section 2.1, several key design principles emerge (**Figure** **10**a). First, structural compatibility between the LA and LB components is critical, avoiding acid‐base neutralization. This means the sites should be unquenched (due to spatial separation) or have polarizable unstable bonding (electronegativity difference and insufficient orbital overlap) for charge redistributions. Achieving this requires precise control over the chemical and geometric environments of the donor and acceptor centers (Figure 10b).^[^ ^120^ ^]^ Strategies such as creating vacancies, inducing structural distortions, tailoring attachment sites, and leveraging hydrogen bonding are valuable design approaches. Second, an appropriate balance between the acidity and basicity of the components is essential for directing the desired reaction pathway (Figure 10c).^[^ ^126^ ^]^ This balance can often be assessed through Bader charge distribution analysis. Third, as with many catalytic systems, achieving the most suitable correlation between reactivity and stability is crucial. Overly reactive pairs may lead to undesirable side reactions, while overly stable pairs may lack sufficient activity. These principles highlight the potential of FLP to be optimized for specific target reactions.

Surface atomic arrangements (e.g., exposure facets) are equally significant for the construction of active FLP and therefore the catalyst activity. As mentioned in previous subsections, certain facets of Al_2_O_3_ and CeO_2_, for instance Al_2_O_3_(110) and CeO_2_(110), feature the effective exposure of LA and reconstruction of surfaces upon post‐synthesis (e.g., the creation of oxygen vacancies) for elongated distances and electron redistribution. Sometimes the polarity of the exposure plane further assists metal‐support interaction. In Ru‐doped MgO(111), Ru single atom, which is anchored by a terminating oxygen atom, forms active synergistic sites with other adjacent oxygen atoms, achieving high conversion for ammonia cracking.^[^ ^127^ ^]^ In contrast, non‐polar MgO(100) and MgO(110) surfaces generate Ru nanoparticles due to the lack of intermolecular force, leading to lower conversion, and potentially different reaction pathways. The surface electron distribution, which atomic arrangement is also associated with, is a simultaneous factor to consider when designing catalysts and investigating the structure‐activity relationship, particularly after the substitution and/or doping of elements. This is the key to evaluating the active sites and the activity strength of these synergistic sites.

Meanwhile, the construction of dual active sites, where one site is an FLP, offers another innovative structural strategy that is worth investigating. This approach leverages the co‐activation capabilities of multiple sites, further expanding the potential of FLP systems. The concept of FLP can be extended to a broader range of fields, as demonstrated by recent studies, in a non‐conventional way. For example, Zheng et al.^[^ ^50^ ^]^ revealed that FLP active sites can form dynamically during reactions, with reaction intermediates serving as active centers. In the methanol‐to‐olefin (MTO) reaction, hydrocarbon pool species, such as polymethylbenzenium (acting as the LA), and deprotonated zeolites (Si−O−Al⁻, acting as the LB), can function as FLP (Figure 10d). Similarly, Mei et al.^[^ ^128^ ^]^ demonstrated that in the cracking of aromatic rings within zeolites, dihydroanthracenes and a single extra framework Al^3^⁺ atom in FAU zeolite can operate as an FLP for H₂ splitting. These findings highlight the versatility of FLP chemistry and its potential to drive innovative catalytic processes.

Due to the complication of these heterogeneous FLP catalysts with varying surface properties as well as active sites, each property needs to be assessed and tuned carefully, by both experimental characterization and computational modelling, for novel catalyst design with better catalytic performances. In the following section, we will introduce the various characterization methods for better verification of FLP sites and interpretation of structure‐activity correlations, as well as mechanistic studies.

## 3 Characterization of FLP in the Solid Materials

Characterizing FLP sites and their reaction mechanisms bridges fundamental research and practical catalysis. Combining advanced experimental and computational tools reveals the spatial, electronic, and dynamic properties of FLP, enabling tailored catalyst design for improved reactivity and selectivity. The following sections detail these techniques and example their integrated use in analyzing FLP active sites and mechanisms.

### 3.1 Introduction of Different Advanced Techniques

**Figure 11:**
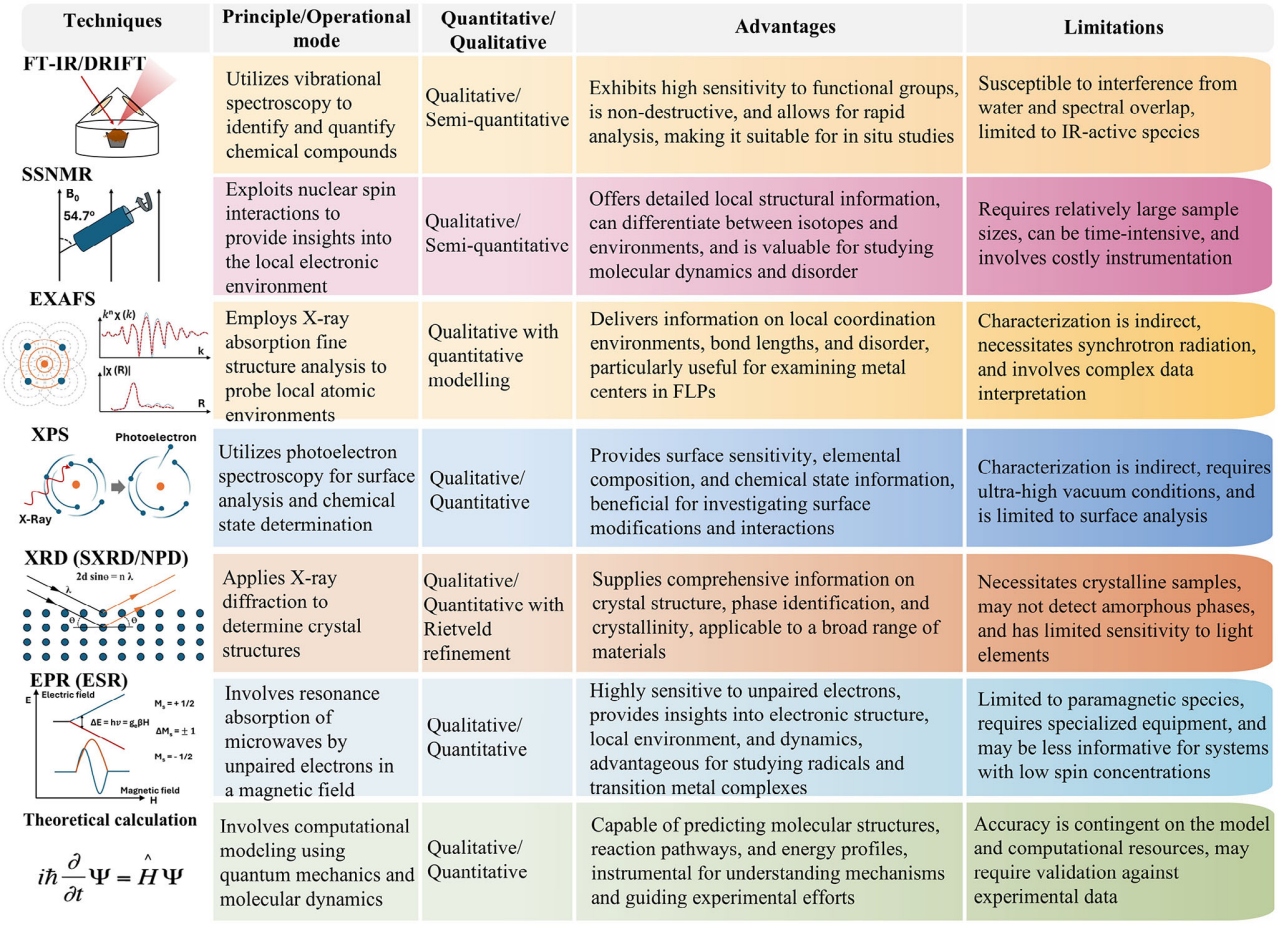
Summary of the different techniques applied for the characterization of FLP active sites on solid materials.

Identifying active sites and understanding their properties are crucial for evaluating and optimizing catalytic performance, guiding the design of more efficient catalysts. Advanced experimental techniques and computational simulations have been extensively developed for this purpose. Key methods, including spectroscopy, microscopy and theoretical modelling (summarized in **Figure** **11**), all provide insightful explorations into the structural, electronic, and chemical properties of active centers. These tools also enable mechanistic studies and reaction pathway mapping, offering a comprehensive understanding of FLP behavior.

#### 3.1.1 Solid‐State NMR

Solid‐state nuclear magnetic resonance (SSNMR) spectroscopy is a powerful technique for probing the atomic‐level structural details of solid materials. It provides critical insights into chemical composition, local environments, and spatial coordination, making it indispensable for characterizing structures and host‐guest interactions in solid‐state catalysts.^[^ ^129^, ^130^, ^131^, ^132^ ^]^ By employing molecular probes, SSNMR can assess the strength, type, concentration, nature, and accessibility of active sites within materials.

The broad line widths in static SSNMR spectra of solids arise from internal nuclear spin interactions, while parameters such as isotropic chemical shift and chemical shift anisotropy (CSA) reveal chemical composition and local environments.^[^ ^133^ ^]^ Multi‐dimensional homo‐ and hetero‐nuclear correlation SSNMR experiments enable direct observation of through‐bond connectivity and through‐space proximity. Techniques like REDOR and RESPDOR provide precise internuclear distances from dipolar interactions. Advances in ultra‐high field spectrometers and ultra‐fast magic angle spinning probes have further enhanced the sensitivity and resolution of SSNMR spectra, as highlighted in comprehensive reviews.^[^ ^134^, ^135^, ^136^, ^137^ ^]^

Over the past two decades, SSNMR has proven invaluable in studying FLP‐based catalysts. It has elucidated the coordination states of LA and LB sites, the spatial arrangement of surface species, and their structural transformations during reactions. Additionally, multi‐dimensional and multi‐nuclear SSNMR has been used to investigate the dynamic behavior of adsorbed small molecules and their interactions with FLP active sites, offering fundamental insights into catalytic mechanisms.

#### 3.1.2 Synchrotron‐Based Techniques

Synchrotrons accelerate charged particles (electrons in this case), in a circular path under vacuum. As electron bunches pass through bending magnets or insertion devices, the resulting magnetic fields deflect their trajectory, causing them to emit continuous electromagnetic radiation tangential to their path. Synchrotron X‐ray radiation offers several key advantages: (1) a monochromatic beam with a broad energy range, spanning infrared to X‐ray regions; (2) ultra‐high photon flux (brightness), enabling superior signal‐to‐noise ratios; and (3) small divergence angles and excellent collimation, ideal for coherent diffraction and imaging applications. These properties make synchrotron‐based techniques–‐such as scattering, diffraction, and absorption methods–‐indispensable for exploring fundamental and applied material sciences, including catalysis. Researchers across disciplines leverage synchrotron radiation to gain deep insights into material structures and dynamics at atomic and molecular scales.^[^ ^138^, ^139^, ^140^ ^]^

##### Extended X‐ray Absorption Fine Structure

X‐ray absorption fine structure (XAFS) is element‐specific, which probes the electronic and local geometric structures of studied elements within the bulk of crystalline and non‐crystalline materials.^[^ ^141^, ^142^, ^143^ ^]^ XAFS is divided into two main components: X‐ray absorption near‐edge structure (XANES) and extended X‐ray absorption fine structure (EXAFS). The XANES region, spanning ≈30 eV below to 40 eV above the absorption edge, provides critical insights into the oxidation state and coordination geometry of absorbing atoms through features such as the absorption edge (fingerprint), pre‐edge region, and overall spectral shape. The EXAFS region, extending from ≈40 eV to 1000–1500 eV beyond the edge, encodes local structural information through oscillations caused by the scattering of excited electrons by neighboring atoms. By curve‐fitting experimental EXAFS spectra to theoretical models (e.g., fast Fourier transforms, FFT), key parameters such as bond lengths and coordination numbers can be determined. In FLP chemistry, ex‐situ and in situ XAS measurements are widely used to track changes in valence states and local environments, providing insights into the nature of active sites and electron transfer mechanisms.

##### Synchrotron X‐Ray Diffraction

SXRD acquires diffraction patterns of materials, polycrystalline materials in our cases, providing details on potential phases, chemical composition, microstructures, and morphology (i.e., exposure facets).^[^ ^144^ ^]^ The high photon flux and exceptional collimation offered by synchrotron light sources result in a better signal‐to‐noise ratio compared to those obtained from conventional laboratory X‐ray diffraction instruments. This enhanced resolution is particularly beneficial for in situ or operando studies, gaining a deeper understanding of the crystal structure and phase transitions of crystalline materials under operational conditions.^[^ ^139^ ^]^ It is often used to identify the phases and microstructures of synthesized solid catalysts, and meanwhile, it visualizes the spatial separation between cooperative LA–LB sites and the states of adsorbed species after structure refinements.

##### Synchrotron Neutron Powder Diffraction

Neutron powder diffraction (NPD) is a complementary technique to X‐ray diffraction. Neutron sources are generated by striking accelerated proton beams at the metal target, and then the neutrons activated after spallation are directed to the beamline. Unlike X‐rays, which interact with materials through electromagnetic interactions with scattering factor scales with electron density, neutrons are scattered by atomic nuclei. This fundamental difference allows neutron diffraction to be particularly sensitive to light elements, such as deuterium, with large positive coherent scattering lengths. It plays an essential role in the research fields of hydrogen storage materials, polymers, and biological macromolecules. Additionally, neutron diffraction is highly effective in distinguishing between isotopes, providing detailed information about isotopic composition and distribution within a sample, including thick samples or complex assemblies.^[^ ^140^ ^]^ Therefore, during the investigation, the NPD spectra will provide additional information on the location of hydrogens, which assists in the depiction of structures, by deuterating hydrogen atoms in the FLP systems.

#### 3.1.3 Fourier‐Transform Infrared Spectroscopy

Fourier Transform Infrared (FT‐IR) spectroscopy is a robust technique based on the absorption of infrared radiation to evaluate the stretching frequencies of various functional groups within chemical compounds. Several FT‐IR techniques have been developed, including transmission absorption, diffuse reflectance (DRIFT), specular reflectance, reflection absorption (RAIRS), and attenuated total reflection infrared spectroscopy (ATR‐IR).^[^ ^145^ ^]^ The advent of Fourier Transform techniques has drastically reduced acquisition times to milliseconds or faster, facilitating high‐resolution real‐time investigations. With a long history of capturing the unique “fingerprints” of chemical substances, FT‐IR enables the monitoring of interactions between adsorbing gas molecules and heterogeneous catalysts.^[^ ^146^, ^147^, ^148^ ^]^ It is an indispensable tool to characterize organic functional groups, synergistic LB‐LA sites, and the reaction intermediates/products in FLP studies.

#### 3.1.4 X‐Ray Photoelectron Spectroscopy

X‐ray photoelectron spectroscopy (XPS) is a surface‐sensitive, element‐specific analytical technique based on the photoelectric effect. It uses high‐energy X‐rays (typically in the keV range) to eject core‐shell electrons, generating photoelectrons.^[^ ^149^ ^]^ XPS is widely used to probe the chemical and physical properties of metal and oxide interfaces, as well as polarization effects, making it a leading method for characterizing catalyst surfaces and their electronic structures.^[^ ^150^ ^]^ XPS provides both qualitative and quantitative information. Qualitatively, shifts in peak positions reflect changes in oxidation states and coordination environments (e.g., surface atomic arrangements). Quantitatively, the atomic percentages of species in different states can be derived from photoelectron peak intensities, and the energy band gap can be evaluated. Similar to XAS, ex situ and in situ XPS are invaluable for studying FLP active sites, revealing variations in the oxidation states and coordination environments of LA and LB components. These insights are critical for understanding electronic interactions and charge transfer processes within FLP systems.

#### 3.1.5 Other Experimental Techniques, Theoretical Calculations Tools, and Notes

In addition to the above advanced techniques, there are other methods for characterizing solid FLP active sites in heterogeneous catalysis, including electron paramagnetic resonance (EPR) spectroscopy,^[^ ^151^ ^]^ temperature programmed desorption (TPD),^[^ ^62^ ^]^ and Raman spectroscopy.^[^ ^152^ ^]^ Meanwhile, computational methods, particularly density functional theory (DFT) and ab initio molecular dynamics (AIMD), have also supported the study of catalytic systems, including FLP chemistry.^[^ ^153^, ^154^, ^155^, ^156^ ^]^ Complementary to experimental evidence, they together enable atomic‐level insights on structural and mechanistic investigations, facilitating the bottom‐up design of novel heterogeneous catalysts. Computational models could examine key factors influencing catalytic performance, such as charge distribution, activation energies, electronic structure, and defect states. They serve as guidelines for establishing structure‐activity relationship, which requires further exploration by experimental characterization.

It is important to note that a single characterization dataset is often insufficient for understanding the complexities of solid‐state systems. To gain a comprehensive understanding of the catalyst's surface, electronic, and catalytic properties, multiple analytical techniques are often combined to use. Integrating results from diverse methods also enhances the accuracy and depth of both structural and mechanistic characterization, advancing both fundamental research and practical applications.

### 3.2 FLP Active Site Characterization

The basics of each characterization technique and its roles in heterogeneous FLP chemistry interpretations are briefly summarized in Section 3.1, respectively. Importantly, to analyze FLP in various materials, combinative techniques are essential to be adopted to reveal the structural properties of solid FLP materials prior to catalysis (including acidity, basicity, coordination environments, atomic arrangements, etc.), as well as the in‐situ changes in these properties with the formation of intermediates. Therefore, in this section, we will explore the combinative use of characterization using a few examples, such as porous materials, metal oxides, and non‐metal materials, highlighting their unique characterization challenges and opportunities. These analyses aim to provide a comprehensive understanding of how FLP active sites enhance the functionality of solid catalysts.

#### 3.2.1 Porous Materials

The porosity of heterogeneous catalysts has attracted a lot of attention due to their merits in reaction efficiency and atomic economy. Possessing the advantages of both molecular FLP catalysts and porous materials, porous FLP catalysts are advancing rapidly, and this section will focus on the characterization of FLP active sites in zeolites, MOF, and COF. A summary table can also be found in Table S1 (Supporting Information).

##### Zeolites

Zeolites are widely used catalysts in the petroleum industry and pollution treatment, composed of TO_4_ tetrahedra (T = Si, Al, P). Their BAS arise from hydroxyl protons in the Al‐O(H)‐Si framework, balancing the charge from low covalent Al according to electroneutrality. The location of Al in the zeolite framework T site determines the accurate distribution of BAS.^[^ ^157^ ^]^ Zeolites can also work as support materials for the hosting of metal ions to create new active sites.^[^ ^158^, ^159^, ^160^ ^]^

**Figure 12:**
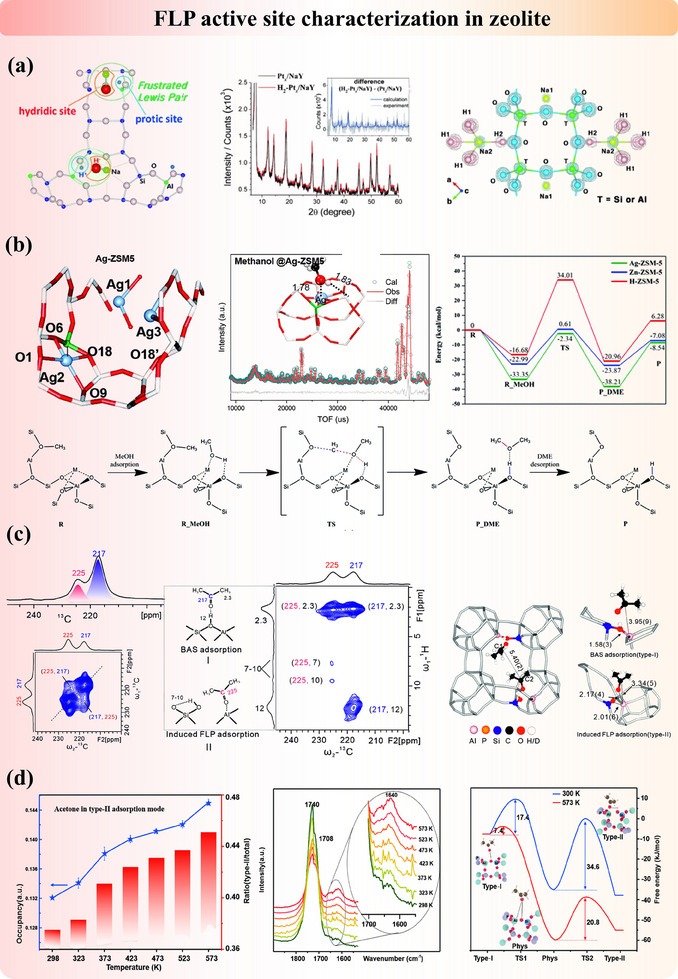
Formation and characterization of FLP in (a) Pt_x_‐loaded zeolite NaY. Adapted with permission.^[^ ^46^ ^]^ Copyright 2015, Wiley‐VCH. And (b) metal‐doped ZSM‐5 zeolite. Adapted with permission.^[^ ^97^ ^]^ Copyright 2021, Royal Society of Chemistry. c) Structural characterization of adsorption‐induced FLP in SAPO34 zeolite by methanol. Adapted with permission.^[^ ^47^ ^]^ Copyright 2021, American Chemical Society. d) Thermal alteration of the structural change between BAS and induced FLP with adsorption of methanol. Reproduced under the terms of the CC‐BY Creative Commons Attribution 4.0 International license (https://creativecommons.org/licenses/by/4.0).^[^ ^49^ ^]^ Copyright 2022, The Authors, published by Wiley‐VCH.

The zeolite‐based FLP catalysts have been reported in recent years. The first zeolite‐based FLP study (dual active site modified FLP) was reported in 2015 by Yoon et al.^[^ ^46^ ^]^ who demonstrated the formation of an FLP in zeolite NaY loaded with Pt nanoparticles (Ptx/NaY). As shown in **Figure** **12**a, the LB and LA of FLP are framework oxygen and charge‐balancing sodium cations, respectively. After heterolytic cleavage of H_2_ on the surface of Pt nanoparticles, H^−^ and H^+^ migrate to form sodium hydride (NaH) and hydroxyl (OH). The authors used in situ NPD to identify the location of the hydride and protonic species inside the framework. It was found that H^+^ species are irregularly located inside the framework due to the absence of their neutron diffraction peaks, while H^−^ species were found to directly bond with the Na^+^ site. Also, they demonstrate that the hydridic site (Na^+^H^−^) can catalyze size‐selectively the esterification of a small aldehyde. In addition to Pt metal, our group explored the incorporation of metal ions, including Fe, Cu, Zn, and Ag, into H‐ZSM5 zeolites to create FLP active sites (modified FLP). The metal site and the adjacent framework oxygen function as LA and LB sites, respectively.^[^ ^97^ ^]^ The positioning of the metal site within the channel was confirmed using SXRD (Figure 12b). We discovered that the synergistic interaction between the FLP active site and the spatially adjacent BAS is crucial for the formation of dimethyl ether (DME). NPD confirmed the adsorption structure of methanol on Ag‐ZSM5, revealing that the C─O bond in methanol coordinates with the Ag site. This interaction weakens the C─O bond, facilitating its reaction with the methyl group formed on the adjacent BAS sites. Consequently, the energy barrier for DME formation over the dual active sites is significantly lower than that in pristine H‐ZSM5, which contains only BAS.

The classical view of active sites in Al‐Si zeolites for molecular adsorption or reaction is generally believed to be on a proton‐attached oxygen bridge between substituted Al and Si (Si─O─(H^+^)─Al) due to charge balance and extra‐framework Al species (LA sites), formed at fixed crystallographic positions on the zeolite structures after their synthesis.^[^ ^157^ ^]^ Our recent study has shown that the adsorption of polar gaseous molecules, such as acetone, methanol, and water, in the pristine SAPO‐type zeolites can induce the Si‐O(H)→Al moiety (BAS) to FLP.^[^ ^47^ ^]^ The electron‐rich oxygen from the hydroxyl of BAS acts as LB, while the adjacent electron‐deficient aluminum serves as LA in this system. This concept, proposed for the first time, is verified experimentally by combined SSNMR, DRIFT, DFT, and structure refinement techniques. As shown in Figure 12c, two distinct peaks at 217 ppm (type‐I) and 225 ppm (type‐II) were observed in the ¹^3^C SSNMR spectrum, with close spatial proximity confirmed by 2D ¹^3^C proton‐driven spin diffusion (PDSD) SSNMR. The major peak at 217 ppm corresponds to ¹^3^C‐2‐acetone adsorbed on BAS, as supported by the literature. The peak at 225 ppm, however, remained ambiguous. Further analysis using 2D ¹^3^C─¹H heteronuclear correlation (HETCOR) SSNMR revealed a dominant correlation at (217, 12) ppm, confirming BAS adsorption. In contrast, the signal at (225, 7–10) ppm indicated proximity to hydrogen‐bonded hydroxyl groups, suggesting adsorption on LAS. SXRD and NPD measurements crystallographically visualized the two acetone states: (1) Acetone molecules coexist within the cage, with a C1 (type‐I) to C2 (type‐II) distance of 5.40(2) Å, consistent with 2D ¹^3^C─¹^3^C PDSD SSNMR results. (2) For type‐I acetone adsorbed on BAS, the C═O to H (BAS) distance is ≈1.56(4) Å. (3) For type‐II acetone, the C═O group leans toward the framework Al (LAS), increasing the Al─O bond distance from 1.58(3) Å to 2.01(6) Å, indicating weakened Al─O interaction. This structure represents an adsorption‐induced FLP active site. Furthermore, with the in situ SXRD study, we found that the increase in temperature can promote the transformation of BAS to FLP sites, as shown in Figure 12d, the occupancy of induced FLP increased with rising temperature.^[^ ^49^ ^]^ In situ DRIFT upon the adsorption of acetone suggests that more LAS‐bonded acetone was formed under the high temperature, matching with the in situ SXRD results. Furthermore, the lower transformation energy between BAS and induced FLP at higher temperatures than that at low temperatures was also verified by theoretical calculations.

Combined with other reported studies, the identification of FLP sites in these systems remains challenging due to the coexistence of intrinsic LA sites (both framework‐associated and extra‐framework species) and BAS, all of which contribute to gas adsorption and catalytic activity. Additionally, the inherent flexibility of the framework introduces further complexity. As a result, conventional techniques such as NH₃‐TPD and pyridine‐adsorbed FT‐IR often fall short in precisely distinguishing FLP sites, although they still yield valuable qualitative insights. To address these limitations, local and bulk structural characterization methods, including XRD, NPD and SSNMR, are widely employed to visualize catalyst crystallography and identify active sites before reaction, as well as to elucidate the adsorption states of intermediates. These experimental findings are often supported by computational studies, which provide complementary evidence for FLP site distribution and interaction mechanisms. Furthermore, in situ DRIFT spectroscopy is highly recommended for real‐time monitoring of dynamic processes, such as the emergence of new vibrational bands or shifts in intensity during catalytic reactions, aiding in the identification of intermediates and the characterization of FLP sites.

##### MOF

**Figure 13:**
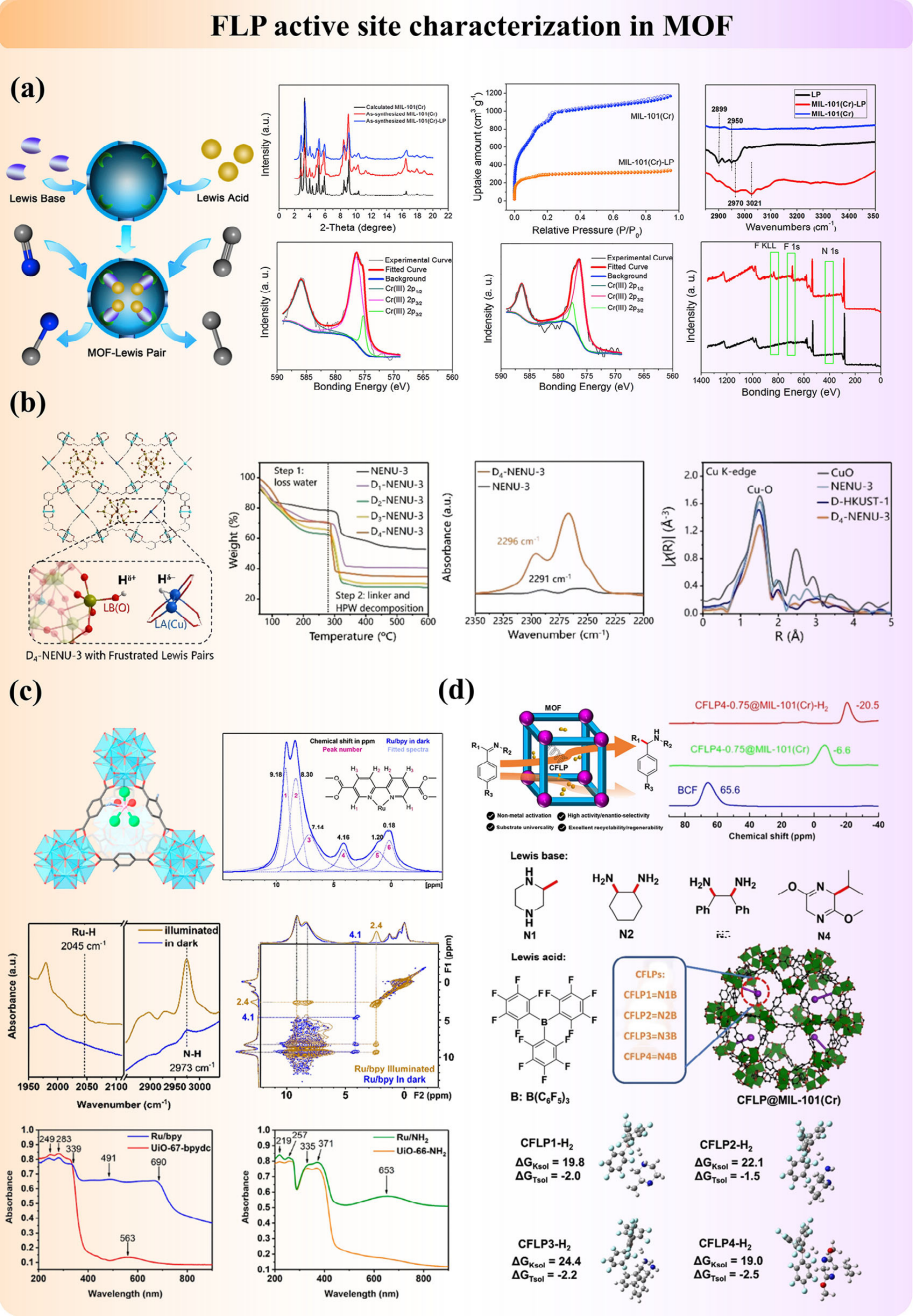
a) Formation and characterization of modified FLP with incorporation of organics in (a) MIL‐101(Cr). Adapted with permission.^[^ ^103^ ^]^ Copyright 2018, Elsevier. b) polyoxometalate (POM)‐based MOF (denoted as D_x_‐NENU‐3). Adapted with permission.^[^ ^161^ ^]^ Copyright 2023, Wiley‐VCH. c) Mechanistic characterization of photoinduced FLP in Ru/UiO‐67‐bpydc for hydrogen activation. Reproduced under the terms of the CC‐BY Creative Commons Attribution 4.0 International license (https://creativecommons.org/licenses/by/4.0).^[^ ^110^ ^]^ Copyright 2023, The Authors, published by American Chemical Society. d) Construction and characterization of modified FLP with chiral centers in MIL‐101(Cr) for product stereoselectivity. Adapted with permission.^[^ ^105^ ^]^ Copyright 2023, Wiley‐VCH.

MOF materials have recently been explored as a new class of heterogeneous catalysts. Their tunable structures and customizable pore environments offer new opportunities for selective catalysis. Heterogeneous FLP in MOF has been extensively developed in recent years, and a key criterion for designing excellent catalysts is to combine high catalytic activity with outstanding recyclability, achievable through the construction of highly efficient heterogeneous catalysts. The feasibility of designing MOF‐based FLP catalysts was first suggested through computational studies in 2015.^[^ ^41^, ^43^ ^]^ A significant experimental breakthrough was achieved by Ma's group in 2018, who successfully introduced FLP into MOF (modified FLP).^[^ ^103^ ^]^ They employed a stepwise approach to synthesize the FLP‐MOF, as illustrated in **Figure** **13**a. MIL‐101(Cr) was selected as the host material due to its large pores (or cages), numerous open metal sites upon activation, and high stability. The LA and LB are anchored within the nano space of the MOF, designated as MIL‐101(Cr)‐LP. To confirm the anchoring of both LA and LB within the cages of MIL‐101(Cr), the authors first used XRD to assess phase purity. The XRD pattern for MIL‐101(Cr)‐LP was consistent with that of pristine MIL‐101(Cr), indicating the preservation of the framework structure during the loading process. The occupation of LA and LB groups within the cages of MIL‐101(Cr)‐LP resulted in a significant decrease in the corresponding BET surface area compared to the pristine material. Additionally, Fourier‐transform infrared spectroscopy (FT‐IR) was employed to study the LA and LB groups within the cages. Furthermore, noticeable peak shifts were observed in FLP@MIL‐101(Cr) compared to the FLP peaks, indicating successful coordination of the FLP N atoms with the open Cr sites of MIL‐101(Cr). In XPS spectra, the higher binding energy in Cr(2p) compared to MIL‐101(Cr) suggests an increase in the electron density of Cr(III), attributed to the interaction between Cr and DABCO. One year later, Ma's group customized the pore environment of MOF and anchored FLP catalysts to it.^[^ ^45^ ^]^ The prepared heterogeneous FLP catalyst demonstrated efficient and selective reduction of imine bonds in α,β‐unsaturated substrates under moderate hydrogen pressure.

Similarly, Liu et al.^[^ ^161^ ^]^ constructed FLP sites in a polyoxometalate (POM)‐based MOF, using unsaturated Cu nodes as LA and POM surface oxygen as LB (Figure 13b). The catalysts, Dx‐NENU‐3 (x = 1–4, reflecting acetic acid addition), were characterized to confirm FLP formation. TGA showed mass loss at 280 °C, indicating ligand removal and Cu unsaturation. FT‐IR with acetonitrile‐d₃ adsorption revealed a peak at 2296 cm⁻¹ in D4‐NENU‐3, confirming stronger Cu Lewis acidity. EXAFS analysis further demonstrated reduced Cu−O coordination, validating the defect engineering strategy for creating 3D porous FLP catalysts. In addition to forming FLP active sites in MOF through the introduction or creation of LA and LB sites, we have recently discovered a novel method for generating FLP active sites in ruthenium (Ru)‐modified UiO‐67‐bpydc (Ru/bpy) upon light illumination (Figure 13c).^[^ ^110^ ^]^ The Ru‐N centre acts as a hidden FLP site, where light‐induced polarization of the Ru─N bond via metal‐to‐ligand charge transfer (MLCT) enables Ru⁺‐N⁻‐mediated solid FLP chemistry, enhancing H₂ activation. Advanced characterization techniques were employed to study this process. ¹H SSNMR revealed gas‐phase H₂ adsorption at 4.1 ppm in the dark, while illumination produced a peak at 2.4 ppm (NH⁺), linked to bipyridine protons (7–9 ppm) in 2D ¹H‐¹H correlation SSNMR, confirming light‐induced H₂ activation. In situ DRIFT spectroscopy further supported this, showing Ru‐H stretching at 2045 cm⁻¹ and N─H stretching at 2973 cm⁻¹ under irradiation, indicative of heterolytic H₂ cleavage by the Ru⁺─N⁻ FLP pair. The weak Ru─H signal suggested partial proton migration, undetected by SSNMR. Incorporating Ru into UiO‐67‐bpydc forms bidentate Ru complexes, enabling efficient electron delocalization. Light‐induced MLCT enhances Ru‐N polarization, creating the Ru⁺‐N⁻ FLP for H₂ activation. UV‐vis spectra revealed MLCT peaks in Ru/bpy, replacing linker‐linker charge transfer (LLCT) peaks, while Ru/NH₂ retained LLCT but showed a Ru d‐d transition peak, leading to slower charge recombination and longer exciton lifetimes, as confirmed by PL and TRPL. This design strategy offers potential for light‐driven FLP chemistry in small‐molecule activation. On the other hand, Ma et al.^[^ ^105^ ^]^ combined computational simulations with experimental data to design a molecular chiral frustrated Lewis pair (CFLP) integrated into a MOF, creating the innovative catalyst CFLP@MOF (Figure 13d). Computational screening of CFLP identified (R)‐2,5‐dihydro‐3,6‐dimethoxy‐2‐isopropylpyrazine (N4) as the most effective LB for H₂ activation, based on its low thermodynamic and kinetic Gibbs free energy barriers. The CFLP@MOF catalyst was synthesized via a step‐wise anchoring method and characterized using ¹¹B SSNMR, confirming its H₂ activation capability. CFLP@MOF combines the high activity and enantioselectivity of homogeneous catalysts with the recyclability and regenerability of heterogeneous systems. This work establishes CFLP@MOF as a pioneering platform for heterogeneous asymmetric hydrogenation, opening new avenues for asymmetric catalytic processes.

Beyond MOF, COF have emerged as another attractive porous crystalline material, particularly for their excellent chemical and thermal stability from covalent bonding constructed by light inorganic elements. COF are also structurally flexible, functionalizable, and electro‐conductive, making them ideal for applications in environmental science and renewable energy. Their porous architecture again provides space for catalyst loading and facilitates substrate access to catalytic centers,^[^ ^162^ ^]^ positioning COF as promising candidates for heterogeneous FLP systems. FLP‐anchored COF have also been reported in recent years. In 2021, Hu et al.^[^ ^107^ ^]^ reported the first COF‐based FLP catalyst, employing a post‐synthetic functionalization strategy to anchor triarylphosphorus (LB) and tris(pentafluorophenyl)borane (BCF, LA) onto a bromine‐functionalized COF. The successful incorporation of these FLP components was confirmed by FT‐IR, XPS, and EDX. ^3^¹P SSNMR provided direct evidence of FLP formation, showing a downfield shift in the phosphorus signal upon BCF coordination, which is the indicative of LA–LB interaction. This COF‐FLP catalyst demonstrated exceptional reactivity for the hydrogenation of alkynes to alkenes, highlighting the potential of COF as versatile platforms for heterogeneous FLP catalysis.

Structure determination is a critical aspect in the development of modified MOF and COF with immobilized FLP. The successful anchoring of FLP molecules within the framework and the structural stability of the resulting materials are commonly evaluated using BET, FT‐IR, TGA, XPS, XAS, and SSNMR. Additionally, ex‐situ SSNMR and in‐situ FT‐IR spectroscopy are utilized to monitor reaction intermediates by tracking shifts or emergence of characteristic vibrational bands. In some cases, theoretical calculations are conducted prior to experimental work to identify optimal combinations of porous supports and FLP molecules, thereby enhancing efficiency and conserving resources. For photocatalytic applications in MOF, optical spectroscopic techniques are indispensable for investigating properties such as band structures and electron‐hole recombination dynamics.

#### 3.2.2 Metal Oxides

Metal oxides are a prominent class of materials for heterogeneous FLP systems, owing to their versatile and tunable surface properties, as well as industrial potential. On metal oxide surfaces, spatial separation between coordinately unsaturated metal atoms (LA) and terminal hydroxide groups (LB) due to defects and/or dopants prevents adduct formation, enabling FLP structures and facilitating the heterolytic dissociation of small molecules. The reactivity of these active sites can be revealed by employing advanced characterization techniques to elucidate these spatial distributions and electronic environment operandoly. Such insights are critical for optimizing metal oxide‐based FLP catalysts, enabling their application in diverse chemical transformations (Table S2, Supporting Information).

##### Aluminum Oxides

**Figure 14:**
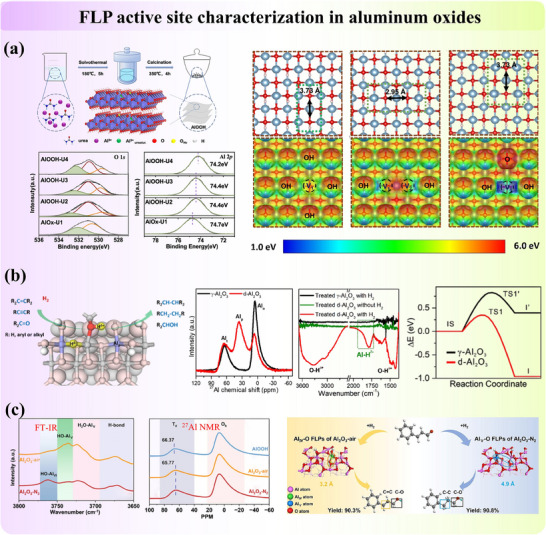
Construction and characterization of FLP on (a) AlOOH. Reproduced under the terms of the CC‐BY Creative Commons Attribution 4.0 International license (https://creativecommons.org/licenses/by/4.0).^[^ ^164^ ^]^ Copyright 2022, The Authors, published by Springer Nature. b) defective Al_2_O_3_. Adapted with permission.^[^ ^63^ ^]^ Copyright 2024, Royal Society of Chemistry. And (c) γ‐Al₂O₃ with different calcination. Adapted with permission.^[^ ^62^ ^]^ Copyright 2023 Elsevier.

Aluminum oxide (Al_2_O_3_) is a vital ceramic material renowned for its exceptional hardness, high melting point, and low electrical conductivity. These properties make it indispensable in diverse technological applications, including electronics, optics, biomedicine, and mechanical engineering, and its industrial utilization is continuously innovated. Balajka et al.^[^ ^163^ ^]^ recently demonstrated that Aluminum rehybridization facilitates bonding with subsurface oxygen atoms, significantly stabilizing surface reconstruction. This finding contrasts with the traditional view, which attributes structural reconstruction primarily to oxygen desorption from the outermost layers at high temperatures. In catalysis, gamma alumina (γ‐Al_2_O_3_) is the most widely used support due to its high mechanical strength, high surface area, and cost‐effectiveness. However, despite its extensive application, the structure of γ‐Al₂O₃ remains poorly understood. This complexity arises from the diverse surface species and defective atomic arrangements present on different exposed facets, which underscores structural characterization and mechanistic studies. In 2011, combining experimental and DFT studies, Sautet et al.^[^ ^34^ ^]^ discovered that unbonded Al···O pairs on H₂O‐covered γ‐Al₂O₃ surfaces can serve as active sites for CH₄ dissociation to form Al─CH_3_ and O─H. Later, the role of water was explored in more detail in a subsequent publication by the same group, and they demonstrated that the optimised partially dehydroxylated γ‐Al_2_O_3_ surface could activate methane and dihydrogen at low temperatures and also bind N_2_ at the active Al(III) sites.^[^ ^58^ ^]^ DFT results show that partial hydroxylation stabilizes the metastable (110) surfaces, alters the electronic structures of the Al─O FLP, and affects the reactivity of Al and O sites, which increases the Al atom's acidity and the O atom's basicity. Therefore, the design and stabilization of surface FLP on (nonreducible) metal oxide surfaces can be accomplished through proper surface water residue. Recently, Liu et al.^[^ ^164^ ^]^ recently constructed solid surface FLP on layered AlOOH (**Figure** **14**a), where a Lewis basic OH⁻ site and an adjacent Lewis acidic unsaturated Al^3^⁺ site near an OH vacancy (OHv) form the FLP active sites, enabling efficient H₂ activation for hydrogenation. Characterization techniques, including TEM, XRD, FTIR, XPS, EPR, TGA, and ^2^⁷Al SSNMR, confirmed the layered structure, OH defects, and FLP formation. Particularly, EPR identified that OHv were key defects, and XPS revealed the electron‐rich unsaturated Al arose from OH vacancies. Their DFT calculations supported the FLP model, showing a low H₂ dissociation barrier (0.16 eV) for the OHv configuration. Besides, Zheng et al.^[^ ^63^ ^]^ reported the construction of FLP on pentacoordinated Al^3^⁺‐enriched defective Al₂O₃ (d‐Al₂O₃), where Al₅ sites and surface oxygen species act as LA and LB, respectively, enabling heterolytic H₂ activation to form O‐H⁶⁺ and Al‐H⁶⁻ without transition metals (Figure 14b). ^2^⁷Al SSNMR and in situ FTIR confirmed the presence of FLP and heterolytic H₂ activation, and DFT calculations supported a stepwise hydrogenation mechanism, where hydride transfer from Al‐H⁶⁻ precedes proton transfer from O‐H⁶⁺. The d‐Al₂O₃ catalyst was proposed to exhibit broad applicability, efficiently hydrogenating olefins, alkynes, and polar unsaturated bonds. Yang et al.^[^ ^62^ ^]^ prepared two Al_2_O_3_ catalysts with different FLP sites from AlOOH by calcination in air and N_2_ atmosphere (Al_2_O_3_–air and Al_2_O_3_─N_2_), respectively (Figure 14c). Characterizations by ^2^⁷Al SSNMR, EPR, and XPS confirmed the presence of unsaturated Al^3^⁺ sites near OH vacancies, which act as LA sites, while surface O atoms serve as LB sites. ^2^⁷Al SSNMR showed a shift to higher fields due to electron shielding from OH defects, indicating the formation of FLP. In situ FT‐IR confirmed the presence of LA–LB pairs, with Al₂O₃–air exhibiting Al_IV_–O FLP sites and Al₂O₃─N₂ featuring Al_V_─O FLP sites. DFT calculations further supported the FLP structures, showing that Al_IV_─O sites favour vertical adsorption of C═O bonds, while Al_V_─O sites enable flat adsorption of both C═C and C═O bonds, leading to different hydrogenation selectivity.

##### Indium oxides

**Figure 15:**
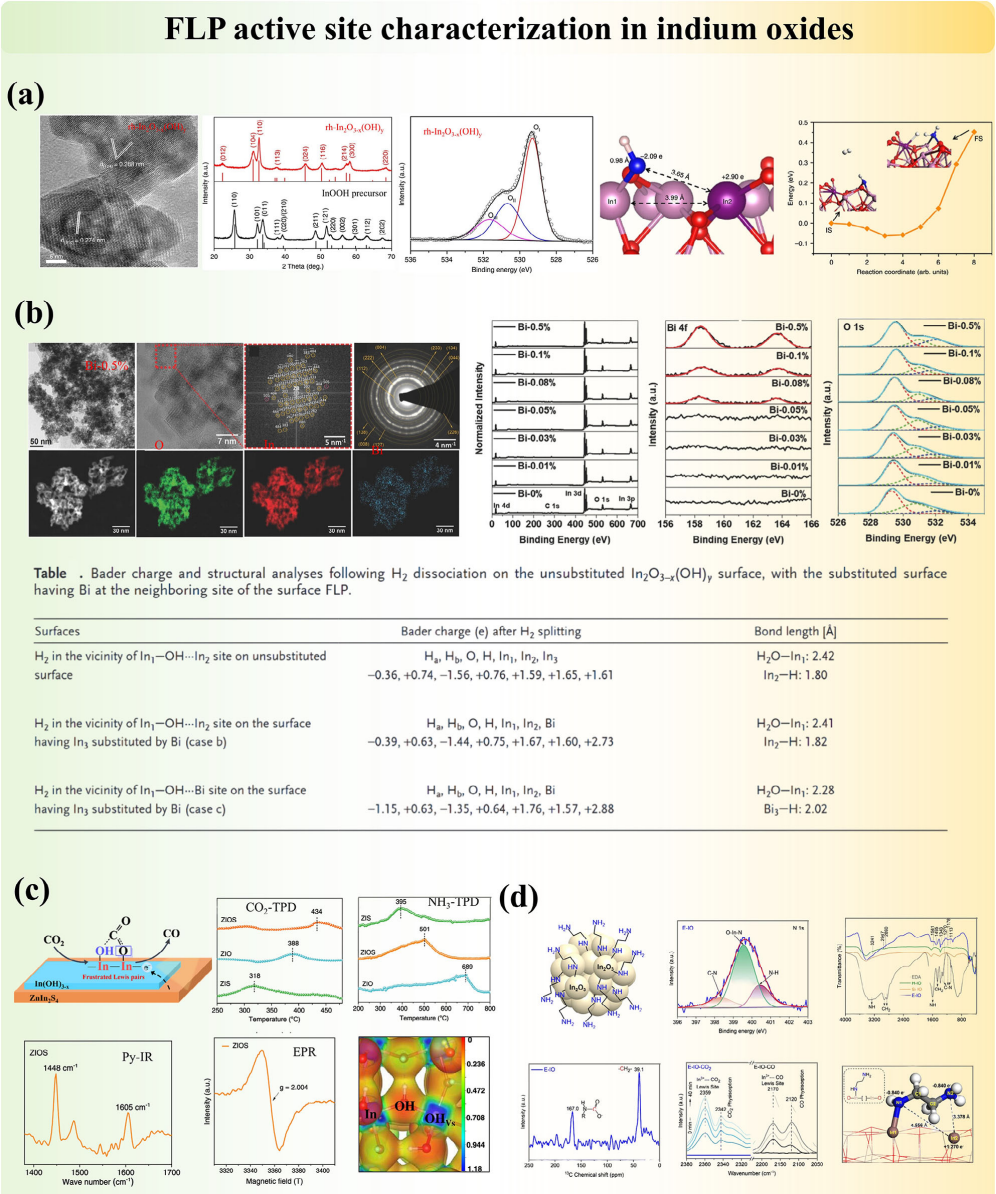
Construction and characterization of FLP on (a) rhombohedral polymorph of rh‐In_2_O_3_−x(OH)y. Reproduced under the terms of the CC‐BY Creative Commons Attribution 4.0 International license (https://creativecommons.org/licenses/by/4.0).^[^ ^165^ ^]^ Copyright 2019, The Authors, published by Springer Nature. b) Bi‐substituted In_2_O_3−x_(OH)y. Reproduced under the terms of the CC‐BY Creative Commons Attribution 4.0 International license (https://creativecommons.org/licenses/by/4.0).^[^ ^166^ ^]^ Copyright 2018, The Authors, published by Wiley‐VCH. c) ZnIn_2_S_4_/In(OH)_3–x_ heterojunction. Adapted with permission.^[^ ^168^ ^]^ Copyright 2023, American Chemical Society. And (d) NH_2_ substituted In_2_O_3–x_(OH)_y_. Reproduced under the terms of the CC‐BY Creative Commons Attribution 3.0 Unported license (https://creativecommons.org/licenses/by/3.0).^[^ ^102^ ^]^ Copyright 2024, The Authors, published by Royal Society of Chemistry.

Indium oxide (In_2_O_3_) is a typical surface FLP catalyst.^[^ ^35^, ^36^, ^60^ ^]^ The FLP on In_2_O_3_ surface comprises a coordinately unsaturated Lewis acidic In atom, proximal to an oxygen vacancy and an adjacent Lewis basic hydroxide group, which enables the heterolysis of H_2_ and therefore assists CO_2_ reduction to form either CO or CH_3_OH.^[^ ^74^, ^165^ ^]^ In 2019, Ozin et al.^[^ ^165^ ^]^ discovered that the rhombohedral polymorph of rh‐In_2_O_3−x_(OH)_y_ was around two orders of magnitude more reactive than the cubic form of c‐In_2_O_3−x_(OH)_y_ for the production of CO or CH_3_OH in CO_2_ hydrogenation (**Figure** **15**a). XRD, HRTEM, and XPS spectroscopy confirmed the presence of oxygen vacancies and hydroxyl groups, which are crucial for forming FLP. DFT calculations revealed that the rhombohedral structure facilitates stronger charge separation between In^3^⁺ (LA) and OH⁻ (LB) sites, with a larger Bader charge difference (+2.90 e and −2.09 e) compared to the cubic form. This results in a lower activation energy barrier for H₂ dissociation (0.45 eV) and more efficient CO₂ hydrogenation.

Inspired by the fact that catalytic activity can be tuned through elemental variation, the catalytic performance of In_2_O_3‐x_(OH)_y_ was optimized through isomorphic substitution of In^3+^ with Bi^3+^ (Figure 15b).^[^ ^166^ ^]^ HRTEM and SAED confirmed the cubic structure of In₂O₃ and the uniform distribution of Bi^3^⁺ within the lattice. XPS revealed the presence of surface defects by identifying the surface species, including oxygen vacancies and hydroxides, which are crucial for catalytic activity. DFT calculations suggest that the 6s^2^ electron pair of Bi^3+^ hybridizes with the oxygen in the neighboring In‐OH LB site, leading to mildly increased Lewis basicity without influencing the Lewis acidity of the nearby In LA site. Meanwhile, Bi^3+^ can act as an extra acid site, serving to maximize the heterolytic splitting of reactant H_2_ and resulting in a more hydridic hydride for efficient CO_2_ reduction. In their following work, the DFT results further supported the enhanced reactivity of Bi^3^⁺‐O^2^⁻ pairs, showing shorter Bi─O distances and higher charge differences compared to In^3^⁺‐O^2^⁻ pairs, which facilitate H₂ dissociation and lower activation energy for CO₂ hydrogenation.^[^ ^167^ ^]^ Recently, Wang et al.^[^ ^168^ ^]^ reported the ZnIn_2_S_4_/In(OH)_3–x_ (ZIOS) heterojunction for visible‐light‐driven CO_2_ reduction, where ZnIn_2_S_4_ (ZIS) harvests the light and In(OH)_3–x_ with FLP activates the CO_2_ (Figure 15c). CO₂‐TPD and NH₃‐TPD revealed strong basic and acidic sites, respectively, with desorption temperatures at 434 °C for CO₂ and above 501 °C for NH₃. Py‐IR spectroscopy confirmed the presence of LA sites, characterized by peaks at 1448 and 1605 cm⁻¹. EPR spectra provided direct evidence of hydroxyl vacancies, further supporting the formation of FLP. DFT calculations and electrostatic potential analysis demonstrated that the hydroxyl groups (In─OH) serve as electron‐rich LB, while the adjacent hydroxyl vacancies (In─O) act as electron‐deficient LA. Also, ethanediamine was used to functionalize the In_2_O_3–x_(OH)_y_ with the hydroxyl group substituted by an amino group, and this new material is proven to contain an InNH_2_⋯In surface FLP and its CO_2_ photocatalytic performance is demonstrated to outperform that of its unfunctionalized In_2_O_3−x_(OH)_y_ (Figure 15d).^[^ ^102^ ^]^ XPS confirmed the presence of Ov and amine groups, with a higher Ov concentration (34.43%), enhancing the Lewis acidity of In(III) sites. FTIR spectroscopy and SSNMR revealed successful grafting of ethylenediamine (EDA) onto In₂O₃. In situ DRIFTS demonstrated CO and CO₂ adsorption on unsaturated In sites, confirming LA–LB interactions. DFT calculations validated the InNH₂⋯In FLP configuration, with shorter distances (3.378 Å) enhancing synergistic interactions for CO₂ and H₂ activation. The combined Lewis acidity, basicity, and oxygen vacancies significantly improved CO₂ adsorption and activation.

##### Cerium Oxides

**Figure 16:**
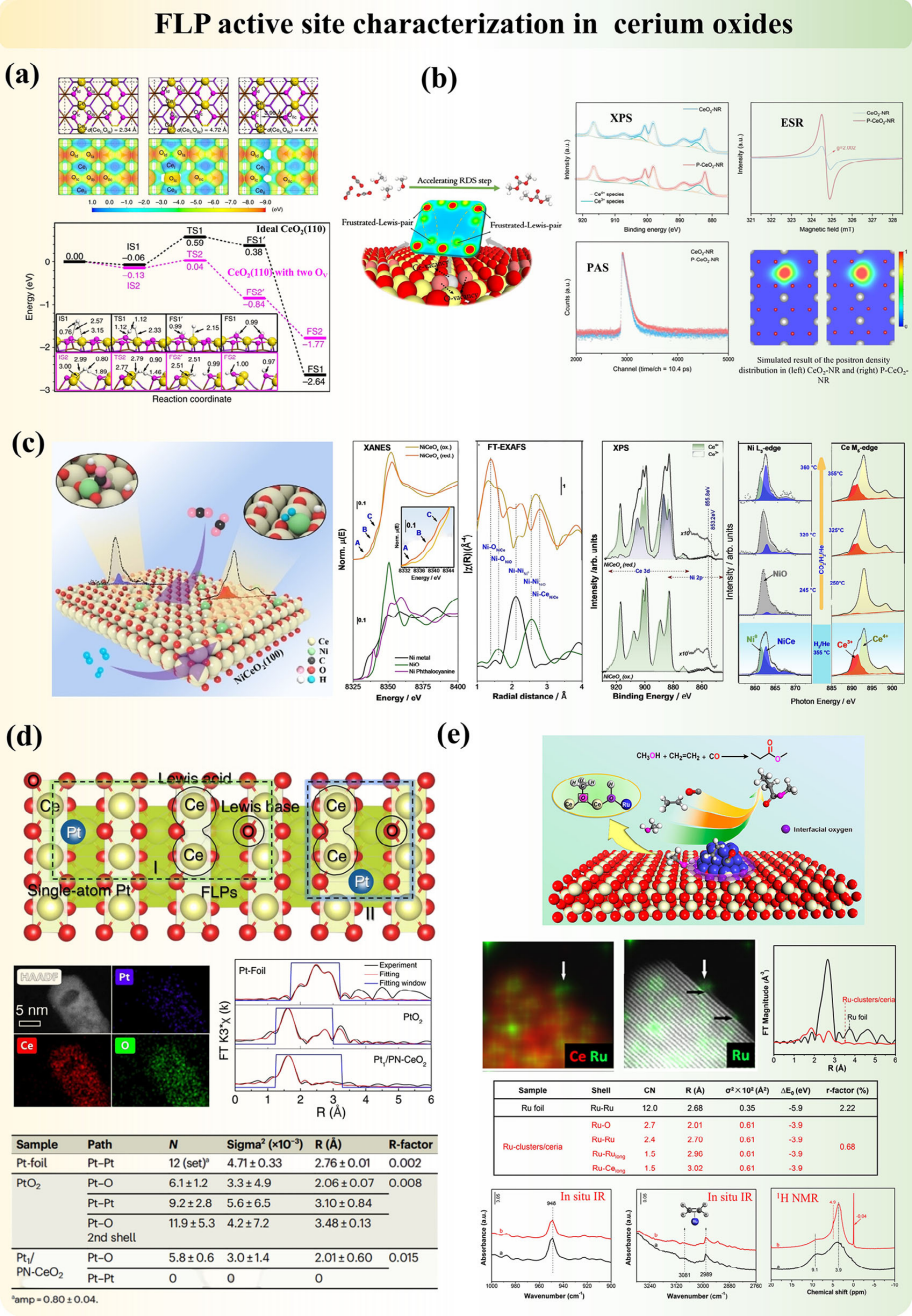
a) Computational simulation of FLP on CeO_2_. Reproduced under the terms of the CC‐BY Creative Commons Attribution 4.0 International license (https://creativecommons.org/licenses/by/4.0).^[^ ^66^ ^]^ Copyright 2017, The Authors, published by Springer Nature.^[^ ^66^ ^]^ Construction and characterization of intrinsic FLP on (b) partially reduced CeO_2_ nanorods. Adapted with permission.^[^ ^170^ ^]^ Copyright 2022, Wiley‐VCH. Characterization of modified FLP on (c) Ni‐substituted CeO_2_. Adapted with permission.^[^ ^171^ ^]^ Copyright 2023, Wiley‐VCH. d) Pt‐impregnated CeO_2_. Adapted with permission.^[^ ^173^ ^]^ Copyright 2022 under a CC‐BY 4.0 license. And (e) Ru‐impregnated CeO_2_. Adapted with permission.^[^ ^174^ ^]^ Copyright 2018, American Chemical Society.

The strong reducibility of cerium metal in CeO_2_ enables the adoption of the material in diverse catalysis. Surfaces with (111), (110), and (100) orientations are commonly studied because these are thermodynamically most stable.^[^ ^169^ ^]^ The energetic cost of forming an oxygen vacancy in ceria is relatively low, and the FLP site of (Ce, Ce)‐O comprises adjacent Lewis acidic Ce^3+^ and lattice O^2−^. Qu et al.^[^ ^59^, ^66^ ^]^ conducted detailed investigations on the influence of oxygen vacancies’ positions on the generation of surface FLP in CeO_2_ through DFT calculations. They found that all FLP are formed when surface Ov was regulated to spatially separate Lewis acidic (Ce^3^⁺) and basic (O^2^⁻) sites in elongated Ce···O distances (≈4 Å) (**Figure** **16**a). DFT calculations reveal that FLP sites on CeO₂(110) with two adjacent oxygen vacancies exhibit a low activation energy (0.17 eV) for heterolytic H₂ dissociation, with the hydride being stabilized at the Ce site. In addition to the single surface Ov on the CeO_2_ to create FLP active sites, recently, Xe et al.^[^ ^170^ ^]^ proposed the introduction of vacancy clusters on CeO_2_ to construct real FLP active sites, which consist of sterically hindered Ce^3^⁺ (LA) and lattice oxygen (LB) sites near surface defects (Figure 16b). The FLP environment was thoroughly investigated using various techniques. XPS detected an increase in Ce^3^⁺ species, indicating the formation of more surface defects, and a higher concentration of oxygen vacancies in the partially reduced CeO₂ nanorods (P‐CeO₂‐NR) was suggested by ESR. XAS further identified the presence of Ce‐O dimer and O‐Ce‐O trimer vacancies, which are crucial for forming FLP sites.

On the other hand, the catalytic activity of FLP on ceria has been found to be enhanced by incorporating heteroatoms into CeO_2_. This structure modification method alters the properties of FLP, as replacing Ce^3+^ ions with other metal ions would modify their Lewis acidity, as well as the local electronic/geometrical structures. Zafeiratos et al.,^[^ ^171^ ^]^ synthesized Ni‐substituted CeO₂ nanoparticles, which exhibited exceptional Ni mass‐specific activity and nearly 100% methane selectivity (Figure 16c). Operando characterization techniques, including XAS and XPS, revealed that ionic Ni and Ce^3^⁺ surface sites are crucial for the reaction, while metallic Ni formation does not significantly enhance activity. DFT calculations suggested multiple active sites that Ce‐O FLP and Ni‐O CLPs facilitate H₂ dissociation, with Ce‐O FLP enabling heterolytic H₂ dissociation to form Ce‐H and O‐H species without an energy barrier, while Ni‐O CLPs also promote H₂ dissociation with a low energy barrier of 0.11 eV. Hua et al.^[^ ^81^ ^]^ reported that gallium substitution can also produce novel single‐atom catalysts on ceria surfaces where the doped metal has a Lewis acidic nature, resulting in a lower energy barrier for dihydrogen activation. In the study of Qu et al.^[^ ^83^ ^]^ lattice substitution of Ce^3+^ by La^3+^ could lead to surface FLP structure reconstruction and their DFT results suggested that the smaller Bader charge on La^3+^ decreased the Lewis basicity of (La, Ce)‐O FLP site, resulting in a weak adsorption of CO_2_. Similarly, Xu et al.^[^ ^172^ ^]^ synthesized a CeO_2_ photocatalyst with cooperatively tailored N‐substitution that promote light absorption, CO_2_ bonding and activation, multiple proton‐electron transfer processes, and C─C coupling reactions.

Apart from this, Qu et al. introduced a single Pt atom onto the surface of porous nanorods of CeO_2_ (heteroatom impregnation). This dual‐active site catalyst, consisting of Pt single‐atoms and FLP, realized a stable H_2_ generation at a low temperature.^[^ ^173^ ^]^ The key innovation lies in the synergistic interaction between Pt single‐atoms that activate methanol and FLP on ceria, facilitating water dissociation. Structurally, EDS spectra presented the uniform distribution of Pt on CeO_2_, and XAS provided insights into the coordination of Pt species. Mechanistically, DFT calculations revealed that FLP on CeO₂(110) surfaces significantly lowered the energy barrier for H₂O dissociation, enabling spontaneous water activation. Additionally, (Figure 16d). Later, the reaction mechanism of these dual‐active sites between Pt clusters and FLP on porous CeO_2_ nanorods was further updated by Qu et al.^[^ ^121^ ^]^ XPS and Raman spectroscopy confirmed the presence of Ce^3^⁺ and oxygen vacancies, essential for FLP formation. DFT revealed that FLP on CeO₂(110) surfaces exhibit the strongest CO₂ adsorption energy (−2.45 eV) and optimal activation, with elongated C═O bonds and reduced O─C─O angles. This means that the FLP sites, rather than the metal and support interfaces, enhanced the adsorption and activation of CO_2_, and meanwhile significantly weakened CO adsorption on FLP to facilitate CO release and suppress the CH_4_ formation. Similarly, Wang et al.^[^ ^174^ ^]^ reported the high activity of nanostructured Ru/ceria (Ru‐clusters/ceria) in the ethylene methoxycarbonylation reaction (Figure 15e). The key innovation lies in the identification and utilization of interfacial LA–LB pairs [Ru‐O‐Ce‐Ov], where Ce‐Ov (Ce adjacent to Ov) acts as the acidic site and the interfacial oxygen of Ru‐O‐Ce as the basic site. The EXAFS‐fitting results reveal that the coordination number (CN) of Ru from Ru─Ru bond in Ru‐clusters/ceria is 2.4, which is much smaller than the CN of Ru foil (CN = 12), indicating that Ru is present as ultrasmall clusters in Ru‐clusters/ceria. And the interfacial LA–LB pair [Ru‐O‐Ce‐Ov], acts as the active site for the dissociation of methanol and the subsequent transfer of hydrogen to the activated ethylene. The FLP characterization is highlighted through detailed in situ IR and ¹H SSNMR studies, which reveal the generation of Brønsted acidity from methanol dissociation at the Ce‐Ov site and the transfer of hydrogen species to ethylene via the interfacial oxygen.

##### Other metal Oxides

**Figure 17:**
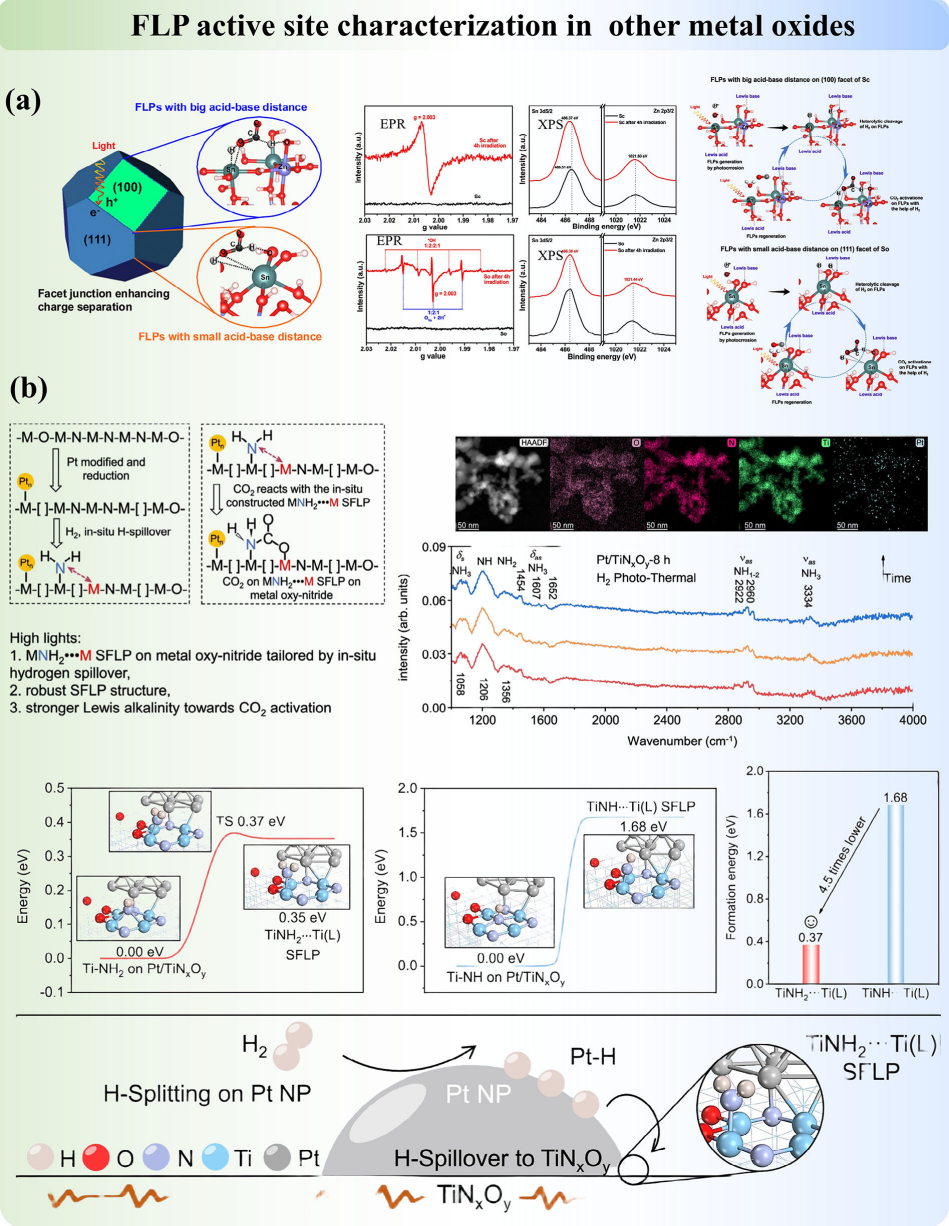
a) Synthesis and characterization of FLP on ZnSn(OH)₆ cubes. Adapted with permission.^[^ ^175^ ^]^ Copyright 2022, Elsevier. b) Characterization of FLP on Pt/TiN_x_O_y_ induced by hydrogen activation on Pt NPs. Adapted with permission.^[^ ^117^ ^]^ Copyright 2024, Springer Nature.

In addition to the study of FLP on the above conventional metal oxides, recent efforts have expanded to explore FLP on a broader range of metal oxides, driven by the desire to harness the diverse electronic and structural properties of different metal oxides for catalytic functionalities and improved efficiencies. For example, Zou et al.^[^ ^109^ ^]^ reported the FLP active site by irradiating ZnSn(OH)_6_ cube with (100) surface. The FLP can be constructed between the photo‐induced Ov and its proximal light‐stable Zn−OH terminal hydroxyls. Besides, they found that the FLP with different acid‐base distances can be created by irradiation on ZnSn(OH)_6_ samples with different crystal facets, specifically the (100) and (111) facets.^[^ ^175^ ^]^ They synthesized single‐crystal ZnSn(OH)₆ cubes (Sc) and octahedra (So) and created FLP through vacuum irradiation, which selectively corrodes light‐unstable terminal hydroxyls, generating Ov as LA and adjacent hydroxyls as LB. The FLP on the (111) facet of So exhibited a shorter acid‐base distance (0.207 nm) compared to those on the (100) facet of Sc (0.488 nm), leading to stronger orbital interactions and more efficient CO₂ activation. The FLP were characterized using EPR and XPS, with EPR confirming the presence of Ov (g = 2.003) and hyperfine splitting signals indicating the trapping of protons at Ov, particularly on the (111) facet. XPS revealed a decrease in Sn 3d binding energy, confirming the formation of low‐valence Sn^δ⁺^ (δ < 4) as LA sites (**Figure** **17**a).

By solid‐state reaction with Na/NaCl, which forms an amorphous shell of TiO_2–x_(OH)_y_ outside of the TiO_2_ core, Ozin et al.^[^ ^67^ ^]^ synthesized a core‐shell heterostructure of c‐TiO_2_@a‐TiO_2‐x_(OH)_y_, comprised of HO‐Ti‐[O]‐Ti surface FLP embedded in an amorphous shell surrounding a crystalline core, which enables a new genre of chemical reactivity. An avenue to improve the process performance metrics lies in replacing M−OH (LB) with a stronger alternative; an intriguing example is the amine M−NH_2_ in metal nitrides.^[^ ^117^ ^]^ This study establishes a proof‐of‐concept that an amine‐type photoactive surface FLP (MNH_2_⋯M) can be constructed in titanium nitride (TiN_x_O_y_) when integrated with a nanoscale platinum spillover co‐catalyst. This surface FLP, comprising Ti−NH_2_ as the LB and low‐valent Ti as the LA, facilitates the gas‐phase light‐assisted heterogeneous reverse water‐gas shift reaction (Figure 17b). As for the FLP characterization, HAADF‐STEM confirms the structural integrity and distribution of Pt nanoparticles on the TiN_x_Oγ surface. In situ DRIFTS under hydrogen and light irradiation reveals characteristic peaks for NH₂ and NH species, confirming the formation of robust Ti−NH₂ groups. DFT calculations further support the stability of the Ti‐NH₂↔Ti(L) SFLP, with charge population analysis showing the Lewis basic nature of the NH₂ group and the Lewis acidic nature of adjacent low‐valent Ti atoms. The formation energy of the Ti‐NH₂↔Ti(L) SFLP is calculated to be 0.37 eV, indicating its favorable formation. In another study, hierarchical hollow TiO_2‐x_ boxes with FLP structures exhibited 36.6 times higher CO_2_ reduction rate as compared to that of the commercial TiO_2_ nanoparticles.^[^ ^69^ ^]^ The FLP was characterized using XPS, EPR, FTIR, and TGA, confirming the presence of Ti^3^⁺ states and hydroxyl groups, with CO₂‐TPD and NH₃‐TPD revealing enhanced CO₂ and NH₃ adsorption capacities. Theoretical calculations further supported the strong CO₂ adsorption on FLP sites, with an adsorption energy of −1.352 eV, compared to −0.18 eV for TiO₂ without FLP.

Overall, the investigations on the nature and activity of FLP systems in metal oxides require 1) The structural information, including atomic arrangements, defects, valence states, and coordination environments from X‐ray techniques, EPR, SSNMR, TEM, and other methods. The acidity and basicity of sites can be verified via TPD, Bader charge calculation, and other computational methods. 2) The reaction pathway, including formation of intermediates and in situ changes in structures by adopting in situ techniques such as FT‐IR, XPS, and XAS or ex situ measurements.

#### 3.2.3 Metal‐Free Supports

Another promising category of catalyst supports comprise metal‐free materials (e.g., 2D crystalline materials, polymers, and others), which exhibit immense potential for the stabilization of FLP. These materials offer the added advantage that their structures can be tailored to optimize reactivity and selectivity for target reactions, which require the combinative adoption of characterization techniques to verify the successful construction of FLP and investigate the reaction process (Table S3, Supporting Information). In the case of carbon‐based materials, the deliberate incorporation of heteroatoms such as N, B, O, F into the carbon framework disrupts the inherent lattice structure. This disruption redistributes the charge density between the carbon atoms and the heteroatoms, thus establishing the FLP active sites. A number of studies have demonstrated the exceptional catalytic performance of metal‐free FLP systems. For instance, Su et al.^[^ ^176^ ^]^ reported nanodiamond‐based carbon materials doped with electron‐rich nitrogen and electron‐deficient boron for constructing FLP active sites. These materials effectively activated H_2_, achieving a conversion of 97.4% and a selectivity of 99% in the hydrogenation of cyclooctene at 220 °C. Similarly, Liu et al.^[^ ^177^ ^]^ demonstrated that B/N‐codoped carbon catalysts could efficiently hydrogenate aldehydes to alcohols via an FLP active site featuring B‐N/pyridinic‐N, which facilitates H_2_ activation with a remarkably low H−H bond dissociation energy of only 0.36 eV. Kwon et al.^[^ ^178^ ^]^ employed a B/N‐codoped graphite FLP catalyst to convert CO_2_ to C_2+_ alcohols. This system exhibited a faradaic efficiency of 87.9%, with 95% selectivity and demonstrated operational stability for 70 h. Furthermore, Chen et al.^[^ ^179^ ^]^ reported that phosphorus‐doped carbon nanotubes could function as metal‐free FLP catalysts for the hydrogenation of nitroaromatics at temperatures as low as 50 °C. These examples underscore how metal‐free FLPs offer a viable alternative to traditional metal‐based catalysts by combining sustainability with practical catalytic performance.

**Figure 18:**
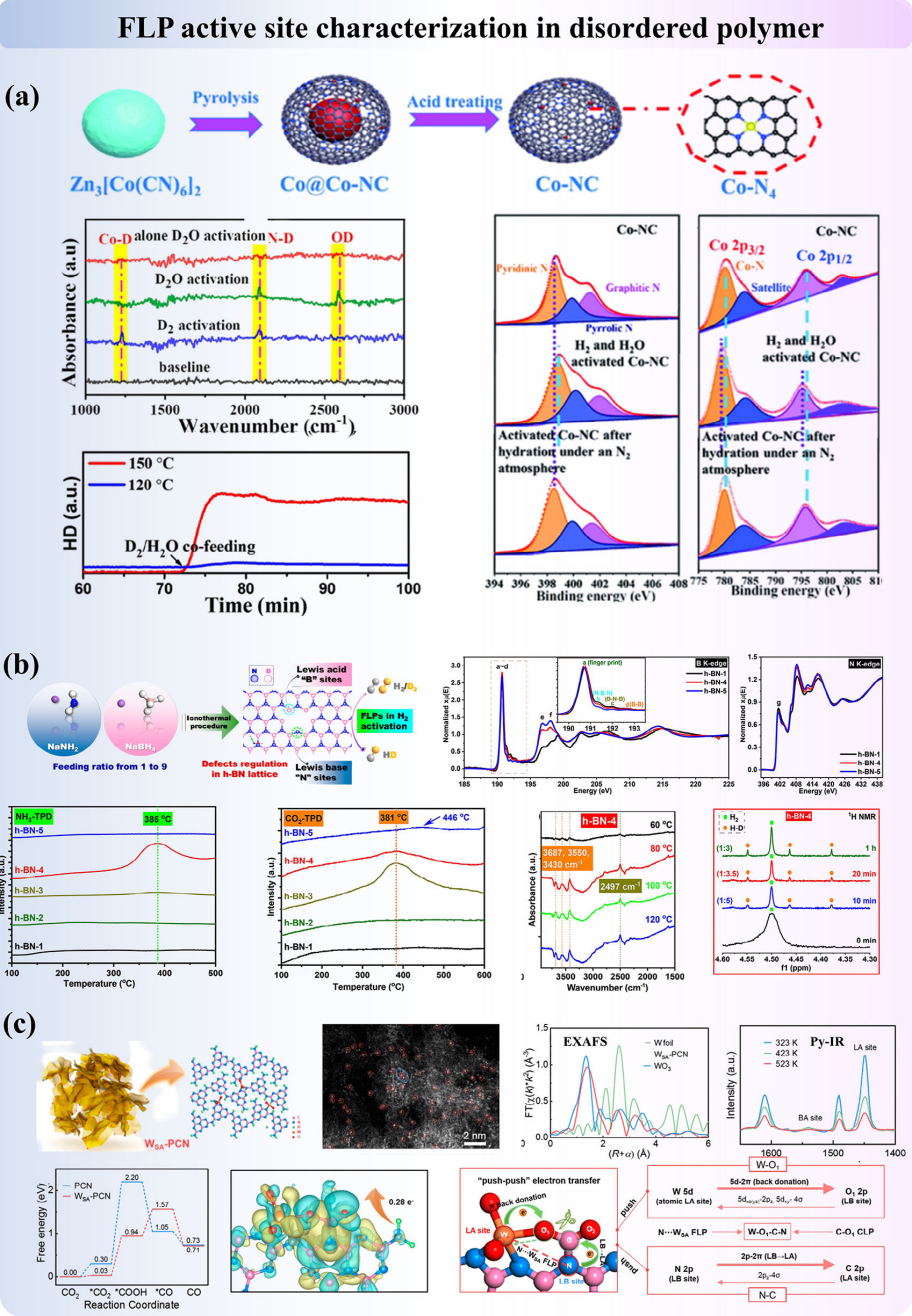
a) Characterization of FLP on defective h‐BN. Adapted with permission.^[^ ^75^ ^]^ Copyright 2022, American Chemical Society. b) Synthesis and characterization of FLP‐mediated bifunctional catalyst, single atom cobalt‐dispersed N‐doped carbon under H_2_‐H_2_O environment. Adapted with permission.^[^ ^52^ ^]^ Copyright 2021, American Chemical Society. c) Construction and characterization of FLP on single atom tungsten‐dispersed polymeric carbon nitride. Adapted with permission.^[^ ^89^ ^]^ Copyright 2025, American Chemical Society.

In early 2012, computational studies suggested that zigzag graphene and graphene nanoribbons could function as heterogeneous FLP catalysts with alternating LA and LB edge substituents.^[^ ^180^ ^]^ This was confirmed experimentally in graphene and graphene oxide nanomaterials by Parvulescu and co‐workers.^[^ ^38^ ^]^ The precise structural control of hexagonal boron nitride (h‐BN), composed of alternating B and N atoms, also enables the creation of active acidic unsaturated B and basic unsaturated N sites within its lattice. Dai et al.^[^ ^75^ ^]^ employed defect engineering to construct FLP active sites on h‐BN, anchoring sterically hindered B centers (LA) and N centers (LB) within its rigid crystalline framework. EXAFS spectra confirmed the presence of these unsaturated B and N defects. TPD profiles validated the coexistence of these sites, with NH₃‐TPD showing a peak at 385 °C for unsaturated B centers and CO₂‐TPD indicating unsaturated N sites at 381 °C. In situ DRIFTS involving H₂ chemisorption on h‐BN‐4 revealed H─H bond cleavage, which was evidenced by the appearance of N─H (3430, 3550, and 3687 cm⁻¹) and B─H (2497 cm⁻¹) stretching modes, confirming FLP‐like behavior. Isotope‐labelling experiments using H₂/D₂ mixtures further demonstrated efficient H₂ activation, with HD formation and a H₂:HD molar ratio of 1:3.5 after 20 minutes. Further supported by DFT calculations, h‐BN with medium‐sized BN₂ vacancies activates H₂ via FLP‐mediated pathway with moderate activation energies, highlighting its potential for hydrogenation reactions (**Figure** **18**a). Recently, our group reported the Co‐N FLP active site on the nitride‐carbon material.^[^ ^52^ ^]^ We found that H₂, traditionally used as a reductant in hydrogenation reactions, can catalyze the transformation of LA–LB pairs (Co─N) into Brønsted acid–base pairs (OH^δ⁻^─Co─N─H^δ⁺^) via a H₂O‐mediated pathway, significantly lowering the activation energy for hydration reactions. In situ ATR‐IR measurements with D₂ and D₂O as probe molecules reveal the heterolytic dissociation of D₂ over the Co─N bond, forming Co─D^δ⁻^ and N─D^δ⁺^ species, which are subsequently converted to Co─OD^δ⁻^ upon interaction with D₂O, confirming the FLP mechanism. XPS analysis further supports this transformation, showing shifts in the Co 2p and N 1s peaks to lower and higher energy regions, respectively, indicating the formation of Co─OH^δ⁻^ and N─H^δ⁺^ bonds after H₂─H₂O activation. Additionally, isotopic labelling experiments using D₂ and H₂O demonstrate the generation of HD and the incorporation of deuterium into the hydration products, providing direct evidence of the H₂‐mediated pathway. DFT calculations elucidate the energy profile of the reaction, showing that H₂ dissociation on the Co─N pair has a low energy barrier (0.51 eV) and that the subsequent H₂O activation to form Co─OH^δ⁻^ is thermodynamically favorable (Figure 18b).

Polymers have also been investigated as good matrices because of their ease in design and pore introduction.^[^ ^181^ ^]^ The polymer matrix with inherent structural irregularities can provide a flexible and tunable environment, allowing for the rational adjustment of the distance and orientation between the LA and LB. Building on the structural‐flexible and disorder‐tolerant feature, Chen et al.^[^ ^182^ ^]^ synthesized two copolymers incorporating bulky borane‐ and phosphine‐containing blocks, effectively constructing macromolecular FLP capable of binding CO_2_ and functioning as recyclable catalysts. Additionally, Yang et al.^[^ ^89^ ^]^ introduced tungsten single‐atoms into polymeric carbon nitride (i.e., inorganic polymer), synthesizing atomic tungsten‐based FLP (N···WSA FLP) sites. The precise design of N···WSA FLP sites, where the electron‐deficient W single‐atom acts as the LA and the adjacent electron‐rich N atom serves as the LB, enables a unique “push–push” electron transfer mechanism for CO₂ activation. The FLP characterization is thoroughly conducted using advanced techniques, including HAADF‐STEM and XAS, which confirm the atomic dispersion of W and the formation of N···W_SA_ FLP sites. Pyridine‐infrared (Py‐IR) spectroscopy reveals the predominance of LA sites (1449 cm⁻¹) over BAS (1540 cm⁻¹), with the LA content closely matching the amount of W single‐atoms, further validating the FLP structure. Theoretical calculations further support the FLP mechanism, demonstrating that the N···W_SA_ FLP facilitates electron transfer from W 5d orbitals to the antibonding 2π orbital of CO₂, weakening the C≡O bonds and lowering the energy barrier for CO₂ reduction (Figure 18c).

As in other materials, the detailed information on structures and mechanisms is essential to characterize FLP in these metal‐free supports. The formation of spatially separated LA and LB sites and the cooperative activity in small molecule activation are required to be justified. So far, we have demonstrated a few techniques with examples emphasizing their roles in FLP characterization, and we encourage their combinative use to reveal well‐rounded chemistry behind FLP.

## 4 Application of FLP in the Solid Materials for Small Molecule Activation

Materials incorporating FLP chemistry, which feature coexisting electron‐deficient and electron‐rich active centers, have been extensively investigated for small molecule activation.^[^ ^55^, ^183^ ^]^ The activity arises from the electron density difference between LB and LA sites, which is thermodynamically favorable for the cleavage of bonds such as H─H, H─X, and C═O. In most cases, these processes are explained through synergistic electron transfer mechanisms involving molecular orbital interactions, leading to heterolytic bond activation.^[^ ^26^ ^]^ Beyond this conventional mechanism, alternative pathways have been proposed, particularly in homogeneous FLP systems. These include electric field effects between LB and LA sites that induce molecular polarization,^[^ ^184^ ^]^ as well as single‐electron transfer that generates frustrated radical pairs capable of homolytically activating substrates.^[^ ^151^, ^185^, ^186^ ^]^ Although these mechanisms are less frequently observed, their contributions to bond activation are non‐negligible.

In this section, we will shed light on the applications of solid‐state FLP systems across three major catalytic fields: thermocatalysis, electrocatalysis, and photocatalysis. A summary of representative applications is provided in Table S4 (Supporting Information). Alongside summarizing the broad applicability of FLP‐based materials, we will also discuss proposed mechanistic pathways for specific reactions, with the aim of providing valuable insights and inspiration for future research.

### 4.1 Thermocatalysis

#### 4.1.1 Hydrogen‐Related Activation

Motivated by the pioneering research in homogeneous thermocatalysis, which ingeniously activated hydrogen gas molecules by the cooperative FLP active centers, more efforts have been dedicated to investigating the activation and hydrogenation reactions for producing valuable chemicals. The Stephan group published some reviews on the diverse use of homogenous FLP with hydrogen for the reduction of organic substrates (including alkenes, alkynes, amines, and so forth), and revealed high yield as well as product selectivity.^[^ ^31^, ^187^ ^]^ They also summarized the feasibility of FLP‐derived transfer hydrogenation reaction in the absence of hydrogen gas, of which FLP abstracts hydrides/protons from additional organic molecules (H‐source). Conversely, FLP catalyst systems can also assist in dehydrogenation reactions. Benefiting from synergistic active sites and a wide variety of homogeneous FLP systems, more organic molecules can be synthesized in different reaction pathways. However, the rapid deactivation upon air exposure, low turnover frequency and challenging product separation of most homogeneous FLP catalysts constrained the industrial application. Instead, a dynamic production is desired, which is achievable by heterogeneous FLP systems. Similar to homogeneous FLP systems, the ability of heterogeneous FLP to break the H‐H bond is a determinant for different kinds of hydrogenation reactions. Dating to the 20^th^ century, there had been experimental evidence from FT‐IR spectra suggesting that H*X* (*X* = OH, H, SH, NH_2_) molecules are adsorbed on MgO surfaces in the form of Mg(*X*)─O(H).^[^ ^188^ ^]^ The detection of OH bands and the corresponding evaluation using isotopes showed agreement with ab initio calculations in terms of stretching frequencies. In the article, the authors emphasized the functionality of ν(OH) to probe the states of coordination of surface Mg^2+^ and O^2−^, highlighting the high resolution of this technique and the presence of intrinsic surface defects for H_2_ activation. Later, more solid materials were evidenced to heterolytically cleave hydrogen with their intrinsically separated LA and LB sites, such as metal sulfides,^[^ ^189^, ^190^ ^]^ CeO_2_,^[^ ^59^, ^191^ ^]^ crystals in the wurtzite structure,^[^ ^37^ ^]^ and others. Noticeably, in the example of both metal sulfides and CeO_2_, the contribution of homolytic dissociation of hydrogen on the bridging S^2‐^ or O^2−^, which further creates anion vacancies, was pointed out. This reaction process further provokes FLP sites on the defective surface and therefore promotes simultaneous heterolytic cleavage.^[^ ^189^ ^]^ This conclusion is consistent with the findings that calcination (H_2_ or CO) could generate vacancies for the construction of FLP sites.^[^ ^59^, ^192^ ^]^ The detection of the simultaneous formation of M‐H and O‐H bonds, the conversion dependence on both sites, and the atomic arrangements on the surface, which visualize spatial distances between sites, become essential to justify activation via FLP, as discussed in Section 3.

Modified FLP systems, which are obtained through the attachment of organic compounds, the substitution or doping of elements, as well as induced FLP, exhibit significant hydrogen activation ability. LA/LB molecules can be immobilized in semisolid FLP,^[^ ^101^, ^193^ ^]^ and porous FLP (particularly MOF) systems. FLP centers are either formed between guest molecules and supports or are both anchored on the support as adsorbates. A detection of HD signal via ^1^H SSNMR H/D exchange experiment was presented to support the room temperature activation reaction in semi‐immobilized triphenylphosphine motifs.^[^ ^193^ ^]^ However, the possibility of proton transfer that does not involve any bond cleavage was not ruled out by the characterization results. Additional ^11^B and ^31^P SSNMR spectra or IR spectrum showing direct evidence of B─H and P─H bonding could further validate the findings. For more active and less porous supports, element substitutions and doping could facilitate existing FLP systems and/or introduce new FLP systems. In B/N co‐doped bilayer graphene,^[^ ^39^ ^]^ the active sites were calculated by DFT to be the carbon atoms around dopants, and they heterolytically cleave hydrogen in sequential order. The authors explained the distinct outcomes of active sites, that the B/N sites are in an extended π network which, from our perspective, may alter the electron density distribution. Therefore, carbon atoms around dopants become the most acidic or basic sites, separately. This example reminds us that there are no definite classifications that could summarize the FLP active sites and reaction mechanisms due to the complications of FLP systems. Therefore, characterizations and computational simulations are compulsory. In the example of Ru/MgO(111), a new FLP system was constructed between the isolated Ru atom and its next‐nearest O neighbor in MgO(111).^[^ ^92^ ^]^ IR, SSNMR, and QENS^[^ ^194^ ^]^ results evidenced the cooperative FLP sites and the proton mobility over the support. Due to the presence of transition metals, contributions from other active sites are crucial to be considered. However, in this system, the homolytic cleavage of hydrogen by ruthenium cations can be excluded according to the high ruthenium oxidation state and the changes observed in both in situ XPS and XAS. The possibilities of dual active sites will be revisited in the following section.

**Figure 19:**
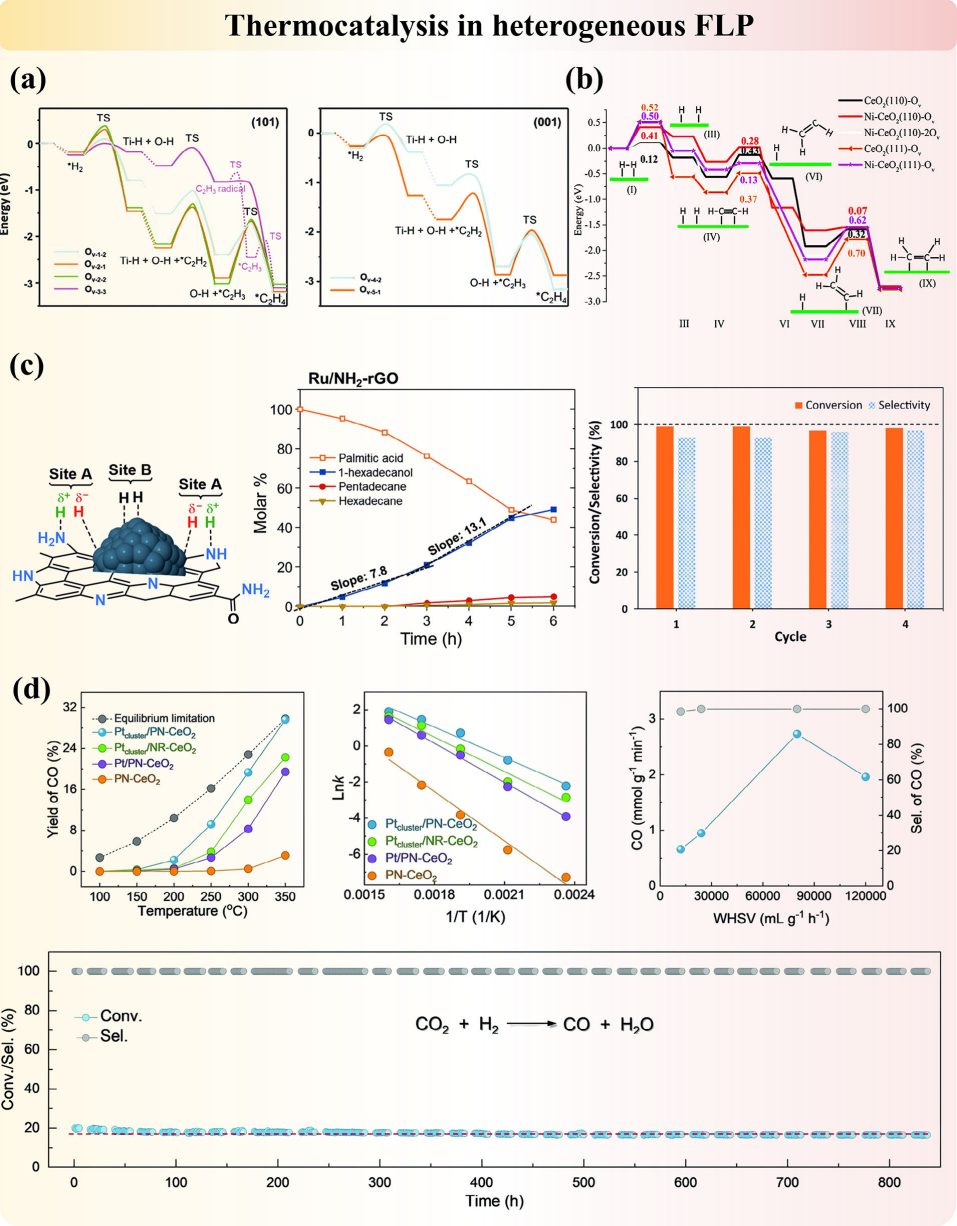
Computation simulation of thermocatalytic selective hydrogenation of acetylene on (a) anatase TiO_2_. Adapted with permission.^[^ ^192^ ^]^ Copyright 2021, Royal Society of Chemistry. And (b) Ni‐substituted CeO_2_. Adapted with permission.^[^ ^79^ ^]^ Copyright 2022, Royal Society of Chemistry. c) Schematic diagram of the mechanisms of reduction of acids on Ru nanoparticles dispersed in N‐doped graphene and reaction performance. Adapted with permission.^[^ ^40^ ^]^ Copyright 2019, Elsevier. d) Mechanistic elucidation of Pt‐supported CeO_2_ in CO_2_ reduction. Adapted with permission.^[^ ^80^ ^]^ Copyright 2023, Wiley‐VCH.

Hydrogen activation is the fundamental and key step for the catalyzed hydrogenation of organic compounds. After identifying both LA and LB sites in the heterogeneous FLP and verifying the H_2_ dissociation abilities, the catalytic performances of FLP systems are often evaluated. Summary table Table S4 (Supporting Information) illustrates the progress on solid FLP in organic synthesis. Diverse solid FLP are found to successfully reduce alkenes, ketones, aldehydes, imines, and other organic molecules, and some FLP systems were found to achieve stereoselectivity, regioselectivity or chemoselectivity. Among all, selective hydrogenation of acetylene (to form ethylene) is the commonly adopted reaction to assess the hydrogen activation capacity of materials. The importance of synergistic active centers with unique spatial separation could be reflected in the varying conversion efficiency while modifying the concentration of LA and LB sites, as in POM‐based NENU‐3.^[^ ^161^ ^]^ DFT calculations suggested the reaction pathway for ethylene formation in TiO_2_^[^ ^192^ ^]^ (**Figure** **19**a) and Ni‐substituted CeO_2_^[^ ^79^ ^]^ (Figure 19b). Initially, hydrogen is dissociated to form metal hydrides (M─H) and hydroxyl groups (M─OH) with low energy barriers in both cases. Then, the weakly adsorbed acetylene will first be attacked by the hydride of M─H species to form C_2_H_3_ species, which accept H of M─OH species at the second hydrogenation step. Before the second step, the C_2_H_3_ radical will either react with H in a radical‐assisted mechanism^[^ ^192^ ^]^ or relax to a more stable adsorption state C_2_H_3_* on the support,^[^ ^79^, ^192^ ^]^ which is site and exposure‐facet dependent. Overall, the rate‐determining step is dependent on the M‐H binding strength and the adsorption strength of the C_2_H_3_ species.

In a similar selective hydrogenation reaction, 93% of chemoselectivity for alcohol was achieved at 99% conversion in Ru nanoparticles/N‐doped graphene (Ru NPs/NH_2_‐rGO), and the catalyst showed rentention of stability and reusability in four cycles. A dual mechanism was proposed (Figure 19c).^[^ ^40^ ^]^ From the classic Dewar–Chatt–Duncanson model and dihydrogen activation theory,^[^ ^195^, ^196^ ^]^ transition metal, Ru^0^ in this case, forms three‐center‐two‐electron complexes with dihydrogen by electron sharing from the H‐H σ orbital to metal d orbitals. With ruthenium being electron‐rich (i.e., nucleophilic), the back donation of electrons from metal d orbitals to H─H σ* orbital is significant, as a consequence, homolytic cleavage of dihydrogen occurs. Meanwhile, due to the presence of basic nitrogen sites in the support, the support better stabilized Ru NPs and the N heteroatom formed FLP with adjacent Ru atoms for an additional heterolytic cleavage pathway. This dual mechamism enables a higher HD formation rate by Ru NPs/NH_2_‐rGO than that of Ru NPs/rGO. The isotope labelling experiment of ^14^NH_2_/^15^NH_2_ indicated the involvement of nitrogen in hydrogenation reactions. In the following acetophenone experiment, it turned out that the Ru^δ+^─N^δ−^ FLP is the key to selectively form fatty alcohols from fatty acids. This example emphasized the importance of mechanistic studies from diverse aspects and the corresponding design of experiments for catalysts with FLP chemistry.

In MIL‐101(Cr), product stereoselectivity is fulfilled by anchoring a chiral FLP at Cr^III^ open site, with B(C_6_F_5_)_3_ (LA) and chiral amines (LB) connecting to one N atom, respectively.^[^ ^105^ ^]^ Under the reaction conditions of 3 mL dry solvent, 20 mg catalyst, 0.2 mmol substrate, and 20 bar H_2_, 48 h at room temperature, an average of 80% of enantiomeric excess value for S isomer was achieved with an average yield of 90% for hydrogenation of different imines. Both XPS and SSNMR measurements supported the heterolytic cleavage of hydrogen. The S isomers are proposed to have a lower energy barrier, and according to the authors, chirality is transferred from the R configuration chiral amine (LB site) to the product under the assistance of hydrogen bonding. However, more detailed mechanistic results are needed to elaborate on the reaction process.

The last example in this section explores the FLP‐mediated bifunctional sites. After activating hydrogen, the FLP cooperative sites can be further modified to provide dual activities for synthesis. Hydration of alkenes and epoxides often requires acidic and water conditions. In single‐atom Co‐dispersed nitrogen‐doped carbon (Co‐NC), after dissociating hydrogen via the Co‐N FLP and forming H^δ−^─Co─N─H^δ+^ pair, the unstable polar Co‐H bond further reacted with H_2_O and resulted in the Brønsted acid‐base OH^δ−^─Co─N─H^δ+^ pair.^[^ ^52^ ^]^ These sites have been evidenced by in situ ATR‐IR, SSNMR, and other characterization techniques, providing a different reaction pathway with lower energy barriers as calculated by DFT. During the hydration of alkene, regioselectivity was maintained following the Markovnikov rule of acid‐catalyzed reaction, and the reaction rate constant significantly exceeded those of traditional acid catalysts such as zeolites. Diols are formed from epoxides of high selectivity in the presence of water with acid from Co─H protonating the epoxides.

#### 4.1.2 Carbon Dioxide‐Related Activation

Carbon dioxide (CO_2_) is one of the most severe greenhouse gases with a long lifetime, that gradually impacts the climate. To slow down global warming and achieve sustainability, CO_2_ capturing and conversion for “net zero emission” have been two tremendous topics over the past decades. Among published catalytic performances of solid FLP, various chemicals are reported to be thermally produced from the hydrogenation of CO_2_, including CO, methane, methanol, and formic acid, based on the FLP‐derived hydrogen molecule or carbon dioxide activation. In the absence of hydrogen sources, C‐C couplings and the formation of other organic molecules are also attainable by FLP chemistry.

The reverse water‐gas splitting reaction (RWGS) has two potential mechanisms – direct cleavage of C─O bond and the format mechanism.^[^ ^80^ ^]^ In both reported CeO_2_ cases – Ni‐CeO_2_ and Pt doped PN‐CeO_2_,^[^ ^80^, ^121^ ^]^ the format mechanism was proved to dominate, giving a high CO formation rate (both < 400 °C). The overall mechanism can be generalized as the hydrogenation of adsorbed CO_2_ to form CO via format intermediate COOH*. To optimize the catalytic performances, a few factors relating to structure design are emphasized by the authors. 1). The appropriate H_2_ activation for both high conversion and selectivity. 2). Weak CO adsorption. However, the two FLP systems proposed different interaction details between H_2_, CO_2_ and FLP sites. In Ni‐CeO_2_, both Ce‐H and Ce^3+^‐OH were detected in H_2_‐pre‐treated sample by in‐situ DRIFT, showing H_2_ is heterolytically activated by Ce‐O FLP sites.^[^ ^80^ ^]^ With the switch of flow to CO_2_ or CO_2_/H_2_ mixture, IR peaks for bicarbonate appeared and Ce‐OH peaks gradually disappeared, suggesting an adsorption geometry of CO_2_. Instead of being trapped at an oxygen vacancy (V_O_), CO_2_ is stabilized by Ce^3+^‐OH in the form of bicarbonate species HCO_3_*. Then the hydride in Ce‐H attacked to form CO via formate intermediate, which was also observed by in situ DRIFT. In the other example case, with the presence of noble metal Pt, a dual active site for activating H_2_ and CO_2,_ respectively, was reported (Figure 19d).^[^ ^121^ ^]^ 1.1 wt.% Pt was loaded onto the porous CeO_2_ nanorods, and formed small cluster anchored on the surface with a high disperson > 40%. An astonishing catalyst stability was achieved after 800‐hour conversion test in the fixed‐bed flow reactor – conversion retained at around 20% with 100% CO selectivity. Based on the H_2_/D_2_ isotope experiments and the detection of Ce^3+^‐OH in DRIFT, hydrogen is suggested to be activated on Pt clusters. It is then followed by hydrogen spillover to the main reaction sites – ceria FLP sites where CO_2_ is adsorbed and activated in the form of bicarbonate. This migrating H* then reduces the bicarbonate to CO*. Additionally, they have suggested that the remaining oxygen will meanwhile replenish V_O_, and reform FLP sites with H* later. In their study, they also pointed out the Pt aggregation after long‐term stability test, while due to the presence of the dual active sites, conversion was not significantly impacted. Further studies on the structures of spent catalyst would also help elaborate this point.

Interestingly, the formation of format is one of the steps in the three‐step mechanism to produce formic acid in Al‐embedded nitrogen‐doped carbon.^[^ ^197^ ^]^ In the material, hydrogen is calculated to dissociate heterolytically on C/N‐Al FLP sites with CO_2_ weakly bonded with the Al‐H moiety. Then the first hydride on Al‐H is transferred to HCOO* intermediate, followed by the transfer of the second hydride from the second activated H_2_ molecule to the adsorbed formate, forming the final product. There is also a simulated two‐step mechanism in Ga‐embedded nitrogen‐doped carbon^[^ ^197^ ^]^ and UiO‐66‐P‐BF2,^[^ ^43^ ^]^ including the heterolytic cleavage of H_2_ on synergistic active sites and successive hydrogen transfer of both hydride and proton. DFT simulated reasonable reaction pathways with low energy barriers in each case, offering innovative ideas for experimentalists to explore. To achieve the activity, the support moiety should feature weak CO_2_ adsorption strength, otherwise, as mentioned by Ye et al.,^[^ ^43^ ^]^ strongly binding CO_2_ will be activated prior to H_2_, leading to poor selectivity. Meanwhile, the binding sites of CO_2_ as well as the hydrogen dissociation rates also impact the selectivity to form formic acid over CO.

**Figure 20:**
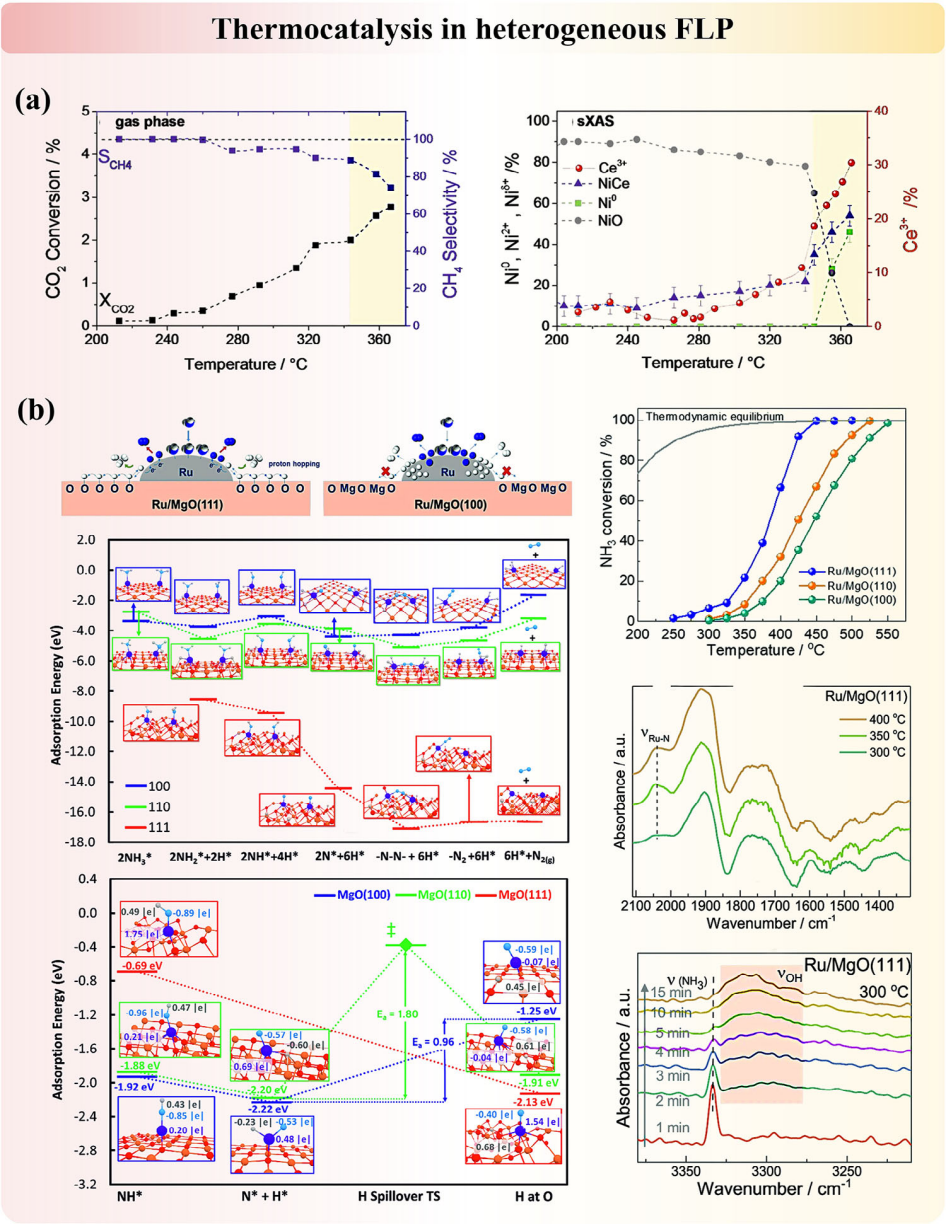
Thermocatalytic application and mechanistic elucidation of (a) Ni‐embedded CeO_2_ for methane formation. Adapted with permission.^[^ ^171^ ^]^ Copyright 2023, Wiley‐VCH. And (b) Ru‐doped MgO(111) for ammonia decomposition. Reproduced under the terms of the CC‐BY Creative Commons Attribution 4.0 International license (https://creativecommons.org/licenses/by/4.0).^[^ ^127^ ^]^ Copyright 2023, The Authors, published by Spinger Nature.

Reported mechanisms of methane^[^ ^171^ ^]^ and methanol^[^ ^42^, ^104^ ^]^ formation from CO_2_ are more complicated. Using both operando spectroscopy and DFT simulation, Barreau et al.^[^ ^171^ ^]^ highlighted the role of three sites for achieving 100% methane selectivity in Ni‐embedded ceria (Ni‐CeO_2_) (**Figure** **20**a). In the first step of H_2_ dissociation, the activation energy by both Ce─O FLP and Ni─O CLP was calculated to be low, indicating the dual active sites. CO_2_ was bound to surface OH groups and Ni‐Ce CLP sites are responsible for the subsequent H capture and methanation reaction. For the formation of methanol in UiO‐67‐NBF_2_, three possible reaction pathways, each of which has a series of concerted two‐hydrogen transfer steps, were simulated.^[^ ^42^ ^]^ Meanwhile, the site preference for H_2_ cracking over CO_2_ adsorption was emphasized again. Using theoretical intermediates as a reference, more experimental evidence is needed to study the FLP‐activated CO_2_ hydrogenation.

Heterolytic cleavage of hydrogen via FLP is essential to hydrogenate the weakly adsorbed CO_2_ species. In the absence of hydrogen, some heterogeneous FLP catalysts could activate organics coupling with CO_2_. In contrast, these systems need to have stronger CO_2_ capture ability for activation. Copolymer consisting of e^−^ accepting‐center borane and e^−^ donating‐center phosphine is able to reversibly capture and release CO_2_, with changes in chemical shifts in ^31^P SSNMR spectra and peak intensity changes in UV‐vis spectra.^[^ ^182^ ^]^ Meanwhile, the CO_2_ adsorption triggers the micellization of polymers, which functions as a catalyst. Upon the addition of PhSiH_3_ (reductant) and amines, a turnover number (TON) over 12 000 to form formamides (at room temperature within 3 hr) was achieved. Compared to the reaction condition using CO_2_ and H_2_, pressure and temperature were significantly lowered.

Monomethylcarbonate (MMC)^[^ ^198^ ^]^ and phenylethylene carbonates^[^ ^83^ ^]^ were experimentally found to be formed by reacting CO_2_ with methanol and styrene in CeO_2_, respectively. Focusing on the former case, since both CO_2_ and methanol have strong interaction with CeO_2_ support, there would be many competing side reactions (e.g., methanol dehydrogenation) and other reaction pathways that result in the formation of the product. Salusso et al. focused mainly on the FLP‐related mechanistic study. FTIR once again became the key characterization technique to identify the adsorption states of reactants. By locating the bands in the mid‐IR region, methanol was found to bridge two Ce^3+^ atoms in the form of a methoxide group with the release of H_2_O. CO_2_ adsorption had an electronic interaction with Ce^3+^, proved by UV‐vis and XPS, thus, a bidentate carbonate configuration that bridges the FLP for charge transfer was proposed. Then, the stretching frequencies of MMC were tracked in the FTIR spectra. The formation of acetic acid from CO_2_ and CH_4_ is another complicated co‐conversion reaction that is in the preliminary research stage. DFT calculations offer insights into structure design, which mostly involve the incorporation of metal single atom into the support, i.e., CeO_2_^[^ ^122^ ^]^ and In_2_O_3_.^[^ ^199^ ^]^ Two different mechanisms were presented. In SA_1_/CeO_2_ (110), a SA‐FLP dual active site was suggested, which concurrently activates CH_4_ and CO_2_.^[^ ^122^ ^]^ In contrast, in Cu_1_/In_2_O_3,_ the Cu‐O FLP site was mainly used to dehydrogenate CH_4_ with Ov adsorbing and trapping CO_2_ for further C‐C coupling.^[^ ^199^ ^]^

#### 4.1.3 C‐X bond Activation

As revealed from the last examples, the C‐*X* (*X* = H, halogen) bond can also be activated by FLP systems. This field is under exploration, with most of the outcomes derived from computational modelling. We encourage more experimental examinations on catalytic performances of systems such as Al_2_O_3_ (Al_III_/O FLP),^[^ ^34^, ^58^, ^200^ ^]^ CeO_2_ (Ce/O FLP),^[^ ^65^, ^82^ ^]^ zeolites^[^ ^50^ ^]^ and albite^[^ ^201^, ^202^ ^]^ for alkane dehydrogenation. Among all, the nonoxidative coupling of methane is the most studied case. Adsorbed CH_3_‐H species are heterolytically activated by the cooperative FLA‐FLB sites, and then CH_3_* species will migrate to achieve C─C coupling, forming C_2_ products.^[^ ^65^, ^82^, ^202^ ^]^ In 2018, Ye et al.^[^ ^201^ ^]^ conducted the experiment in the flow reactor. They reported that using raw albite, a conversion of 3.32% is achieved at 1073 K, with methane gas hourly space velocity (GHSV) of 2 L·gcat^−1^·h^−1^ and catalyst dosage of 0.25 g. Ethane, ethene, and ethylene were the products. They later reported theoretical calculations that correlate the activity of albite to the intrinsic Si/O FLP,^[^ ^202^ ^]^ consistent with the studies in CeO_2_ systems.^[^ ^65^, ^82^ ^]^ Another theoretical study on PMB^+^ confined zeolite by Wei et al.^[^ ^50^ ^]^ justified its activation ability on the H‐H bond and the C‐H bond. More importantly, the system remains stable, and it can dehydrogenate propane via PMB^+^‐O^−^ FLP, forming propene as observed in GC‐MS. The pathway mentioned in the paper involves simultaneous bond breakage of two C‐H bonds by the LA–LB pair, releasing propene and hydrogen directly. Once again, there might be a few competing activation reactions and different reaction mechanisms, depending on the diverse geometrical configurations of propane around the LA–LB entity.

#### 4.1.4 Other Molecules Activation

The application possibilities of heterogeneous FLP in thermocatalysis are endless. Other small molecules, such as methanol^[^ ^47^ ^]^ and ammonia^[^ ^48^, ^127^ ^]^ which have similar bond strength to alkanes, and oxygen^[^ ^203^ ^]^ with a higher bond order can also be catalyzed. In 2021, our group reported the operando creation of FLP in SAPO‐type zeolites for the first time.^[^ ^47^ ^]^ In their study, upon the adsorption of methanol, Si‐O(H)‐Al BAS sites are activated and form Si‐O(H) LB and Al LA sites to capture the adsorbates. The O‐H bond in methanol dissociates upon the action of two sites. The detection of surface methoxy species (SMS) in SSNMR and peak shift in in situ DRIFT measurements with isotopic labelling both supported this abnormal methanol activation process in zeolites. More catalytic results are expected to unmask the product selectivity of the system.

Ru‐O FLP constructed in Ru/MgO(111)^[^ ^127^ ^]^ and Ru/13X zeolites^[^ ^48^ ^]^ were reported to contribute to the ammonia decomposition reaction for green hydrogen production. Particularly in 3.1 wt.% Ru/MgO(111) (Figure 20b), the equilibrium conversion of ammonia is 98–99% at 425 °C under WHSV of 30000 mL g_cat_^−1^ h^−1^.^[^ ^127^ ^]^ The direct evidence of reversible appearance of both Ru‐N and O‐H stretching frequencies in DRIFT spectra highly suggested the FLP chemistry. One important thing about this system is the construction of ruthenium atomic sites by preventing ruthenium agglomeration.

Au‐doped hydroxyapatite (HAP)‐CeO_2_ studied by Guo et al.^[^ ^203^ ^]^ showed a surprisingly high catalytic conversion for the CO oxidation reaction. Since CO is one of the most stable molecules due to its triple bond, O_2_ is more easily activated. 100% conversion was achieved at ≈110 °C under 10 vol% CO gas flow in air (total flow rate: 36.3 mL min^−1^), 200 mg catalyst, and space velocity (SV)  =  200000 mL g_cat_^−1^ h^−1^. Via characterizations such as EPR, Raman, and DRIFT, superoxide O_2_^−^ species were noticed on the surface, which combined with CO to give carbonate adsorbate. The comparison experiments with Au‐doped CeO_2_ and Au‐doped HAP emphasized the linked contribution of this heterojunction system, that FLP may exist between Ce from CeO_2_ and O in HAP for the activation of O_2,_ and with the assistance of Au, CO oxidation occurs.

### 4.2 Electrocatalysis

In Section 4.1, we have introduced a series of catalytic reactions in thermocatalysis, and in fact, researchers have spent most of their studies on the application of FLP in that category. The identification of active sites and adsorbed intermediate species is the key to correlating FLP chemistry with improved conversion efficiency and selectivity. However, as shown above, the presence of multiple reaction sites and different pathways in FLP systems complicates the interpretation of mechanisms, and the studies will vary by case. With additional stimulus imposed for reactions (i.e., electricity, solvents, and light irradiation), more factors and material properties need to be considered. Sections 4.2 and 4.3 will dig into these two fields as the guidance.

First of all, in electrocatalysis factors such as purity of chemicals, temperature of surroundings, setup of electrodes, including catalyst thickness and homogeneity on electrodes, depth of electrodes, distances between electrodes, and calibrations will impact the catalytic test significantly. The review on nitrogen electroreduction reaction^[^ ^204^, ^205^ ^]^ also pointed out the case of gas impurities, which influence the reproducibility between laboratories. In the electrochemical flow cell system, the actual flow and electrochemical potential are additional uncertainties when comparing across the literature. Meanwhile, electrolyte flooding in the gas cell is a remaining issue in the field. Therefore, many precautions are required while conducting the experiments. Secondly, we recommend the adoption of conducting materials that have proven FLP properties for try‐out experiments, for instance, CeO_2_ and carbon materials. The difficulties in ruling out the instrumental errors from conversion will either overestimate or underestimate the catalytic performances, misleading the assessments and elucidations of FLP‐related mechanisms, particularly for the new system.

**Figure 21:**
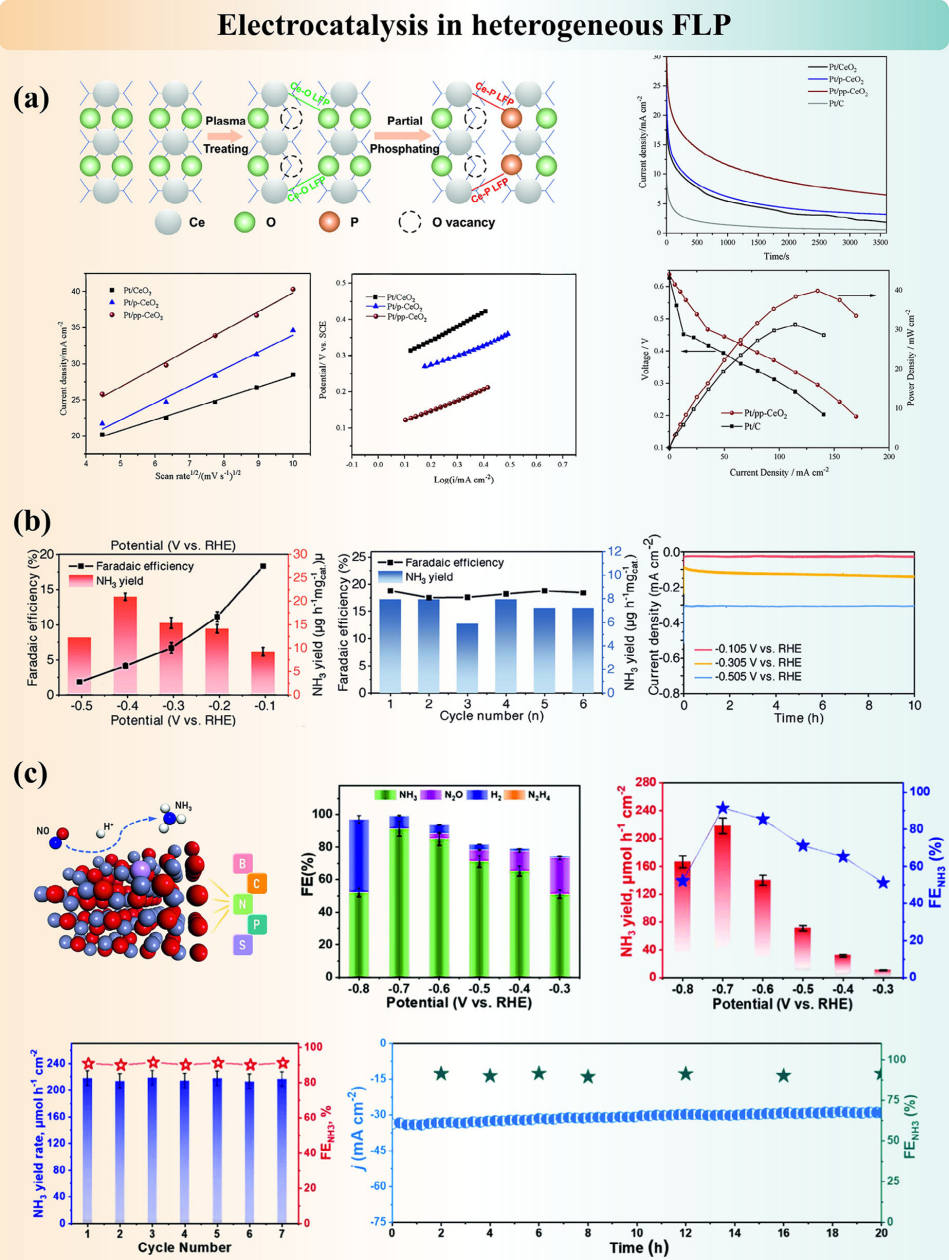
Electrocatalytic application and mechanistic elucidation of (a) Pt NPs doped phospher‐CeO_2_ for direct methanol fuel cell. Adapted with permission.^[^ ^206^ ^]^ Copyright 2023, Elsevier. b) defective boron carbon nitride for the reduction of nitrogen. Adapted with permission.^[^ ^88^ ^]^ Copyright 2022, Wiley‐VCH. And (c) p‐block element doped ZnO(100) for NO conversion. Adapted with permission.^[^ ^85^ ^]^ Copyright 2024, Elsevier.

In 2023, Xu et al.^[^ ^206^ ^]^ explored the application of Pt NPs doped phosphor‐CeO_2_ in the field of direct methanol fuel cell (**Figure** **21**a), showing the possibility of FLP engineering for improving activity, with the current density remaining high after 3600s. In the study, comparison experiments were designed between materials with and without the phosphor dopants. They hypothesized that FLP will activate water molecules and the Ce‐OH formed on the surface will attract CO‐like intermediate to reduce Pt‐poisoning. Then, Pt NPs and FLP will synergistically accelerate the oxidation reaction of the CO‐like intermediate. According to the paper, an improved CO oxidation rate was recorded in Pt/pp‐CeO_2_ due to the stronger activity of Ce‐P FLP than Ce‐O FLP. However, these points remain uncertain. Materials in comparison have different concentrations of oxygen vacancies, which may indicate the intrinsic difference in the population of active sites. Therefore, additional characterizations are essential to the study by providing direct evidence for both Ce─P FLP systems and reaction intermediates. Special flow cells need to be designed for these purposes.

The electrocatalytic reduction of N_2_ and NO was also explored by Dai et al.^[^ ^88^ ^]^ and Zhao et al.,^[^ ^85^ ^]^ separately. Using defective boron carbon nitride, a high Faraday efficiency of 18.9 %, and an ammonia yield of 20.9 µg h^−1^ mg^−1^_cat_ was achieved in the nitrogen activation reaction via the pull‐pull effect on six‐membered ring intermediates (Figure 21b).^[^ ^88^ ^]^ After 6 cycles, the ammonia yield was reported to retain at 90%, showing a good stability of the catalyst. Both the undercoordinated N sites (LB) and boron sites (LA) were experimentally justified. During the isotopic labelling experiment using ^14^N_2_ and ^15^N_2_ as the feed gas, ^1^H SSNMR spectra of electrolytes were recorded and confirmed the source of nitrogen in the ammonium water formed. A GS‐MS measurement provided additional evidence for the cleavage of nitrogen, with DFT calculations suggesting the pull‐pull effect of FLP sites on adsorbed nitrogen. Then, the reaction continued with the attack of H* from the hydrogen evolution reaction (HER) of water in the electrolyte. The competition between H* adsorbate and reactant gas at active sites will impact the reaction efficiency as pointed out by Zhao et al.^[^ ^85^ ^]^ in their case. Ammonia was formed from NO on a P‐doped ZnO(100) catalyst with ammonia production of 218.2 µmol h^−1^cm^−2^ at −0.7 V at 91.4 % FEs N−distal pathway (Figure 21c). The catalyst was catalytically stable, which remained high FE_NH3_ and NH_3_ yield rates over seven cycles. After experimentally proving the source of nitrogen in ammonium, the calculations were used extensively. The substitution of Zn with p‐block element elongated the bond distances between dopant and zinc for FLP formation, then the NO adsorbed (which was calculated to be favored over H* was hydrogenated.

### 4.3 Photocatalysis

In photocatalysis, a suitable energy band gap for electron excitation and efficient charge transfer to prevent electron‐hole recombination are the two main determinants for photocatalytic efficiency. Materials are often designed with element doping, the creation of oxygen vacancies, the formation of heterojunctions, and other modification methods to optimize the two properties. Some of the methods share similarities with FLP construction, which is discussed in Section 2. Thence, we anticipate that the existing FLP in the structure will help the conversion in photocatalysis too. Compared to electrocatalysis, more literature has investigated the application of solid FLP in photocatalytic CO_2_ reduction and nonoxidative coupling of methane.

**Figure 22:**
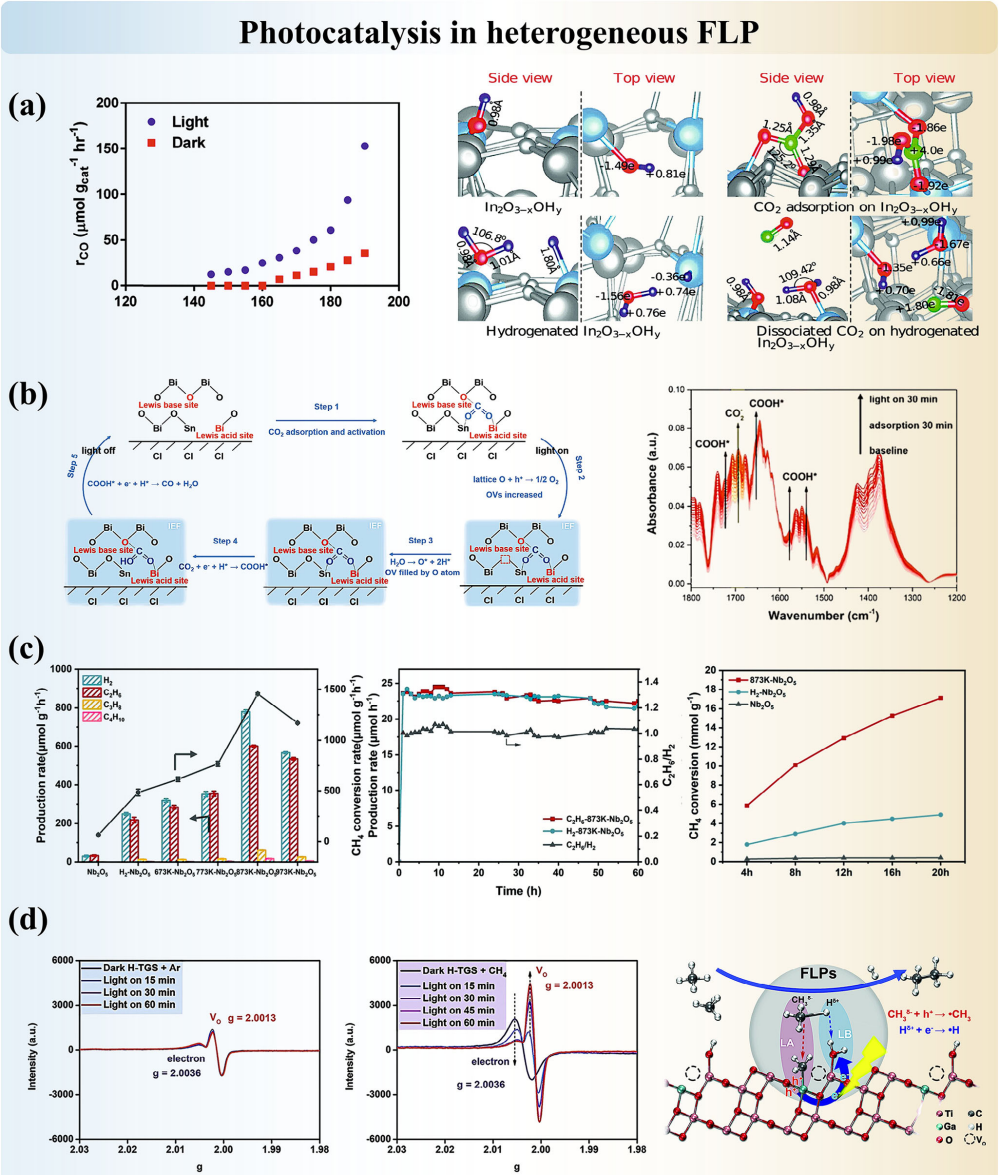
Photocatalytic application and mechanistic elucidation of (a) In_2_O_3‐x_(OH)y for RWGS. Adapted with permission.^[^ ^61^ ^]^ Copyright 2015, Royal Society of Chemistry. b) Sn‐ultrathin BiOCl for RWGS. Adapted with permission.^[^ ^209^ ^]^ Copyright 2024, Elsevier. c) Nb_2_O_5_ for NOMC. Reproduced under the terms of the CC‐BY Creative Commons Attribution 4.0 International license (https://creativecommons.org/licenses/by/4.0).^[^ ^71^ ^]^ Copyright 2023, The Authors, published by Springer Nature. d) p‐block element‐doped TiO_2_ for NOMC. Adapted with permission.^[^ ^68^ ^]^ Copyright 2022, Elsevier.

CO is the main reported product from FLP‐derived CO_2_ reduction.^[^ ^60^, ^61^, ^207^, ^208^, ^209^ ^]^ The study on In_2_O_3‐x_(OH)_y_ pioneered the investigation of LA–LB, highlighting the roles of surface hydroxyl groups and oxygen vacancies that assist the RWGS reaction (**Figure** **22**a).^[^ ^60^, ^61^, ^207^ ^]^ In the dark, stretching frequencies of bicarbonate were identified by in situ DRIFT spectra with flowing CO_2_, while it was assigned to the trapping state of CO_2_ in oxygen vacancy next to In─H and In─OH, rather than being weakly adsorbed on M─OH (Section 4.1). The reaction mechanism was mentioned to be the same in the dark and in the light mode – H_2_ gas is heterolytically cleaved (more direct proof is needed), followed by a H attack from In─H to the adsorbed CO_2_. DFT calculation suggested that the interaction between light and defects will result in slight spatial changes (e.g., changes in OH bond length), which change the energy level of materials from the ground state (GS) to the excited state (ES).^[^ ^60^ ^]^ As a consequence, the reaction became faster under the light due to the lower energy barrier. The impact of the dopant was emphasized in the study of Sn‐ultrathin BiOCl material by Shi et al.^[^ ^209^ ^]^ (Figure 22b). Doping Sn reduced the charge‐transfer resistance as shown in EIS spectra and was calculated to have the lowest work function (WF) by DFT. This is due to the fact that the Sn dopant increases the dipole moment in the system, which enhances the internal electric field (IEF) and consequently promotes the functionality of Bi‐O FLP (more evidence is needed). A better photogenerated charge carrier separation and transfer were achieved for RWGS. One more point to mention is that TiO_2_ might also be a good candidate to investigate for this reaction.^[^ ^210^ ^]^

As emphasized by Chen et al.,^[^ ^71^ ^]^ to stimulate C─H bond activation, a strong polarization environment is required, which is often generated in solid FLP. In their work on NaBH_4_‐treated Nb_2_O_5_ with sufficient oxygen vacancies, a superior methane conversion rate of 1456 µmol g^−1^ h^−1^ was reported for photocatalytic NOMC (Figure 22c).^[^ ^71^ ^]^ The reaction was conducted in a batch quartz reactor using 5 mg of catalyst, 45 mL of methane, and was irradiated under a 300 W Xe lamp for 4 h. LA and LB sites were measured using py‐IR and the distances between sites were obtained from FT‐EXAFS. In‐depth DFT calculation confirmed the synergistic effect of LA and LB for the reaction, and showed that light illumination further induced the charge transfer from LA to LB, which therefore promoted the reaction. The follow‐up long‐term conversion test in their study validated the stability and cyclability of NaBH_4_‐treated Nb_2_O_5_. The improvement in conversion results by enhanced LA and LB activities was reported earlier in p‐block element‐doped TiO_2_ (Figure 22d).^[^ ^68^ ^]^ In situ EPR was adopted to trace the changes in Ov and free electrons, suggesting that CH_4_ was initially adsorbed near Ov_,_ and with electron transfer from the LA Ga site to the LB O site, C─C coupling occurred and released the product. The catalyst retained 60% of its original activity after 6 cycles, together with other catalysts, supporting the better stability of heterogeneous FLPs.

Sometimes the structure benefits from the charge transfer upon light irradiation. H_2_ dissociation ability was discovered in Ru‐UiO‐67/bpydc with Ru atom anchored to two nitrogen ligands. Upon irradiation, metal‐to‐ligand charge transfer (MTLC) was promoted in the Ru‐N bond to form Ru^δ+^─N^δ−^.^[^ ^110^ ^]^ Although the two sites are connected, the electron‐accepting and donating abilities still enable them to perform FLP‐type activation. The charge transfer is reversible, and the material was switched from inactive to H_2_ in the dark. A more detailed explanation and mechanistic study can be found in Section 2. Inspired by this, we are hoping to receive more updates on the application of MOF‐based materials with FLP chemistry in photocatalysis.

### 4.4 Reflections on Heterogeneous FLP in Catalysis

So far, research has focused on three dominant application directions at the laboratory scale. Compared to homogeneous FLP, heterogeneous FLP systems exhibit enhanced water tolerance, greater stability, and improved sustainability. The prevention of water poisoning at active organic FLP molecules can be achieved with porous materials with flexible selection and alternation of supports. For example, the acidic boron sites could be protected by the bulky side groups of organic linkers in SION‐105,^[^ ^211^ ^]^ the PDMS hydrophobic coating layer around MIL‐101^[^ ^212^ ^]^ or the hydrophobic polymer networks POSS.^[^ ^213^ ^]^ Some are reported to be stable in air.

For catalysts which have intrinsic FLP or have been modified with additional elements for constructing active FLP systems, most of them are less water‐ and air‐sensitive. In general, exposure of any catalyst to ambient conditions will result in adsorption of foreign species, while in solid FLP these species can be removed by pre‐treatment which imposes smaller variations on surface structure. In certain cases, the presence of water moisture even assists the formation of FLP or the proceeding of reactions. For instance, an optimal amount of adsorbed water on γ‐alumina serves as the hydroxylation source that stabilizes the metastable termination and enhances the reactivity of synergistic sites.^[^ ^34^ ^]^ Besides, the ability of catalysts to incorporate water into their structure matrix, demonstrated in systems such as defective CeO_2_^[^ ^214^ ^]^ Single‐atom Co‐dispersed nitrogen‐doped carbon,^[^ ^52^ ^]^ Pd‐supported partially oxidized MAX,^[^ ^53^ ^]^ can promote the reaction pathway and/or feature the catalyst's bifunctionality for better catalytic performance.

The ease of handling these catalysts under ambient conditions, along with the intriguing role of water in reaction activation, supports their potential for commercial applications. The commercialization of the solid catalysts with FLP chemistry can be approached from two directions. (1) Development and evaluation of novel systems: Building upon preliminary insights into catalyst structure and properties, further investigation under conditions that simulate industrial processes, such as fixed‐bed flow reactors, is encouraged. These tests, conducted in some reported heterogeneous FLP systems, would provide valuable information on mass transfer behavior, scalability, and catalytic efficiency. It is essential to note that the structural determination for fresh and spent catalysts would also benefit the mechanistic elucidation and FLP catalyst assessments, and relevant research is rarely reported in this field. (2) Reassessment on the existing catalysts for mechanistic insights and structure modification. A thorough dissection of FLP chemistry reveals that its fundamental nature is the involvement of LA and LB sites. This implies, any solid catalysts may feature FLP sites, and careful characterizations on these catalysts with FLP‐mediated pathway would benefit both the rational design and commercialization possibilities. Some typical examples would be CeO_2_ and zeolites, which have been discussed in the main context.

Simultaneously, the synergistic FLP sites on catalysts often suggest more effective adsorption of small molecules and lower energy for bonding activation of molecules such as H_2_, CO_2_, N_2,_ and CH_4_ compared to traditional catalysts,^[^ ^29^ ^]^ which could be the key focus for industrialization. As certain aspects of these phenomena are discussed above, they will not be detailed further here. In summary, the ease of handling of catalysts in air, coupled with the notable role of water in reaction activation using solid FLPs, underscores their potential suitability for commercial applications. However, further catalyst optimization and advancements in engineering development are necessary to achieve industrial utilization.

## 5 Conclusion and Outlook

The field of heterogeneous FLP has witnessed significant advancements, supported by innovative approaches in catalyst design, characterization, and application. Notably, FLP has been recognized as one of IUPAC's 2024 Top Ten Emerging Technologies in Chemistry.^[^ ^215^ ^]^ Building upon the research and innovations achieved to date, we will outline several potential directions here and anticipate significant future breakthroughs in this field.

The exploration of new materials, such as MOF, COF, zeolites, metal catalysts, and non‐metal materials, as supports for FLP will continue to be a vibrant area of research. Particularly, materials offering tunable porosity and functionality could provide an ideal platform for the incorporation of FLP active sites and the regulation of spatial distances by design. The rigid lattices, on the other hand, achieve better structure stability and are often adjustable in terms of surface properties for improving the concentration and activity of active sites, as well as potentially providing multiple active sites with diverse mechanisms.

In the coming years, the focus will also shift toward the design of FLP systems that are sustainable, stable, recyclable, efficient, and dynamic during reactions, aligning with the growing emphasis on green chemistry principles. Deactivation of catalysts due to aggregation, structure distortion, and deterioration will impact FLP activity significantly due to the rigorous standard on spatial separation of synergistic sites in most of the FLP systems. Therefore, the design of target anchor sites, long‐term cycling tests, and (operando) characterization are needed. Also, achieving dynamic production is a critical issue awaiting solution and is eventually related to the optimization of catalytic systems. To achieve better mass transfer, scalability, and efficiency, we encourage more investigations on reactions in fixed‐bed flow reactors based on the preliminary research. Furthermore, the development of FLP‐based catalytic systems for practical industrial applications, such as carbon capture and utilization, could be an important direction for future research. There is also a need for further investigation into the reaction mechanisms involved in FLP‐catalysed transformations of small molecules and the development of new theoretical models to better understand and predict FLP reactivity.

The future of heterogeneous FLP research is poised for exciting developments, and we have pointed out the three fundamental focuses, which will be driven by the convergence of computational and experimental methodologies. The integration of machine learning and AI with advanced characterization techniques will create a powerful toolkit for the discovery and optimization of FLP catalysts, with a better understanding of the relationships between structures and activities, as well as reaction mechanisms.

Machine learning in FLP catalyst design and synthesis: The integration of machine learning (ML) into the design and synthesis of FLP catalysts represents a transformative approach in materials science. ML algorithms can analyze vast datasets to identify patterns and correlations that are not immediately apparent through traditional methods. By leveraging these capabilities, researchers can predict the properties and performance of novel FLP catalysts before they are synthesized. This predictive power accelerates the discovery process, allowing for the rapid identification of promising candidates with optimal LA and LB site configurations. Furthermore, ML can assist in optimizing synthesis parameters, ensuring the reproducibility and scalability of FLP catalysts. As the field progresses, the development of more sophisticated ML models tailored to the unique challenges of FLP systems will be crucial.

AI in Screening, Characterization, and Application of FLP Catalysts: Artificial intelligence (AI) offers unparalleled opportunities for the screening, designing, characterization, and application of FLP catalysts. AI‐driven platforms can automate the high‐throughput screening of FLP libraries, efficiently designing catalysts with desired properties. Recently, a breakthrough in the generative model, MatterGen, has been published by Xie et al.,^[^ ^216^ ^]^ providing a new paradigm for materials design. In characterization, AI algorithms can process complex datasets from spectroscopic and imaging techniques, extracting meaningful insights into the structure and dynamics of FLP systems. This capability is particularly valuable for understanding the subtle interactions between LA–LB sites that define FLP behavior. Moreover, AI can facilitate the application of FLP catalysts by predicting their performance in various reaction environments, thus guiding experimental efforts toward the most promising conditions. The integration of AI into FLP research not only enhances efficiency but also opens new avenues for innovation by uncovering previously unexplored catalyst functionalities.

In situ methods and multimodal techniques for investigating solid FLP systems: The characterization of solid FLP systems has been revolutionized by the development of in situ methods and multimodal techniques. These approaches provide real‐time insights into the structural and functional dynamics of FLP under operational conditions. In situ spectroscopy, such as infrared and Raman, allows for the monitoring of reaction intermediates and the identification of active sites, while in situ X‐ray diffraction and scattering techniques reveal changes in the crystalline structure of FLP‐containing materials. Additionally, multimodal techniques that combine spectroscopic, microscopic, and diffraction methods offer a comprehensive understanding of FLP systems, capturing both macroscopic and molecular‐level phenomena. The continued advancement of these techniques will be essential for unravelling the complexities of FLP catalysis, enabling the rational design of more efficient and selective catalysts.

In conclusion, the field of heterogeneous FLP is at the forefront of catalysis research, offering innovative solutions to complex chemical challenges. By embracing the potential of machine learning, AI, and advanced characterization techniques, researchers can unlock new possibilities for FLP catalysts and illustrate reaction pathways, driving progress toward more sustainable and efficient chemical processes. The continued collaboration between computational scientists, chemists, and engineers will be essential for realizing the full potential of FLP, paving the way for breakthroughs that will shape the future of catalysis.

## Conflict of Interest

The authors declare no conflict of interest.

## Supporting information



Supporting Information
