# Supplementary material for: Recent Advances in Heterogeneous Frustrated Lewis Pair: Synthesis, Characterization, and Catalysis
Source: Adv Mater. 2025 Jun 20;37(48):2502101. doi: 10.1002/adma.202502101 (PMC12676097; doi:10.1002/adma.202502101)
Supplement: Supplementary file 1 — Supporting Information [file ADMA-37-2502101-s001.docx]

**Supplementary Materials**

**Recent Advances in Heterogeneous Frustrated Lewis Pair: Synthesis, Characterization, and Catalysis**

Jiasi Li, Shik Chi Edman Tsang *, Guangchao Li*

J. Li, S.C.E. Tsang

Wolfson Catalysis Centre, Department of Chemistry, University of Oxford, Oxford, OX1 3QR, UK

Email: edman.tsang@univ.ox.ac.uk

S.C.E. Tsang, G. Li

Department of Applied Biology and Chemical Technology, The Hong Kong Polytechnic University, Hong Kong 999077, China

G. Li

Research Institute of Advanced Manufacturing (RIAM), Research Centre for Resources Engineering towards Carbon Neutrality (RCRE), The Hong Kong Polytechnic University, Hong Kong 999077, China

The Hong Kong Polytechnic University Shenzhen Research Institute, The Hong Kong Polytechnic University, Shenzhen 518057, China

PolyU-Daya Bay Technology and Innovation Research Institute, The Hong Kong Polytechnic University, Huizhou 516000, China

Email: [guangchao.li@polyu.edu.hk](mailto:guangchao.li@polyu.edu.hk)

**Table S1.** A brief summary of heterogenous FLP construction, application, and application on porous materials in recent years.

| Catalyst | Active sites | FLA | FLB | Discovery | Catalytic Application | Key evidence chemistry | Ref |
| --- | --- | --- | --- | --- | --- | --- | --- |
| Polyoxometalate-based MOF | Guest and support MOF | Coordination-defect metal nodes of MOF | surface oxygen atoms | Precise control of the structure and spatial distance between LA and LB sites in a porous system to activate H_2_, resulting in high hydrogenation activity. | H_2_ activation for acetylene semi-hydrogenation | DRIFT; DFT | ^152^ |
| MOF, NU-1000 | Introduction of FLP | B(C_6_F_5_)_3_ anchored into NU-1000-LB. | (2,4,6-Me_3_C_6_H_2_)_2_**P**(2-Me-4-COOHC_6_H_3_) anchored into NU-1000-LB | P/B type MOF-FLP catalyst for efficient and selective hydrogenation of nitrogen heterocyclic molecules. | H_2_ activation for N-heterocycles hydrogenation reactions | Mainly proved P/B system in MOF | ^42^ |
| MOF, [Eu(tctb)(H_2_O)]105 | Introduction of FLP | B atoms | Lewis basic diamine substrates | Integrate a bulky LA-functionalized ligand into the water-tolerant MOF and use diamine substrates as LB to form FLP, creating a heterogeneous FLP catalyst. | Diamines into benzimidazoles using CO_2_ as the C1 feedstock | Experiments | ^154^ |
| MIL-101(Cr) | Introduction of FLP | B(C_6_F_5_)_3_ | 1,4-diazabicyclo [2.2.2]  octane | Describe a remarkably clever and facile stepwise to anchor the LA in a MOF strategy that incorporates FLP into a MOF framework. | Reduction of imines by H_2_. | Experiments; FTIR; XPS | ^100^ |
| MIL-101(Cr) | Introduction of FLP | B(C_6_F_5_)_2_(Mes) | 1,4-diazabicyclo [2.2.2]  octane | Introduce LB and LA sites into MOF to achieve efficient catalytic activity for imine reduction, olefin hydrogenation, and chemoselective hydrogenation of α, β-unsaturated organic compounds while maintaining excellent recyclability. | Chemoselective hydrogenation of α,β-unsaturated organic compounds | DFT | ^43^ |
| MIL-101(Cr) | Introduction of LP | B(C_6_F_5_)_3_  anchored into MIL-101(Cr) | *(R)*-2,5-dihydro-3,6-dimethoxy-2-isopropylpyrazine anchored into MIL-101(Cr) | Incorporate chiral frustrated Lewis pairs (CFLPs) into achiral MOFs to bypass complex chiral ligand synthesis and enable easy tuning of catalytic performance by adjusting the type and amount of CFLPs in CFLPs@MOFs. | Asymmetric hydrogenation | DFT; NMR | ^102^ |
| B(C_6_F_5_)_3_/MIL-101@PDMS | Introduction of LP | B(C_6_F_5_)_3_ | 1,4-dioxane substrate | B(C_6_F_5_)_3_ in the MIL-101 framework, coated with a hydrophobic polydimethylsiloxane, preventing water from entering the MOF nanocage and facilitating 1,4-dioxane diffusion form FLP. | Hydrogenation of benzaldehyde | Experiments | ^155^ |
| ZIF-67-MW@SiO_2_-DMDES | Introduction of LP | Coordination-defect Co metal nodes of MOF | N in 2-methylimidazole | ZIFs prepared by the microwave-assisted method show more defects, and the independent LA and LB sites in the defective ZIFs construct a heterogeneous FLPs system. | hydrogenation of unsaturated aldehydes | Experiments; DFT | ^156^ |
| MOF | Introduction of LP | B atoms | N atoms | Develop guidelines to construct active FLP sites in porous organic scaffolds for CO_2_ hydrogenation via large-scale screening of 25,000 immobilized FLPs. | Direct catalytic CO_2_ hydrogenation to formate | DFT | ^120^ |
| FLP@PCN-521 | Introduction of LP | Tris (2´,3´,5´,6´-tetramethylbiphenyl-4-carboxylic acid) borane | Bulky organic compound with N and O centre atoms | Present a new way to construct FLP with both ends fixed through geometry restriction in the MOF backbone, which allowed for the separation of influences from the electrostatic effect and distance to the catalytic performance of FLP. | Cyclic carbonate from CO_2_ to investigate the influence of distance in FLP | SSNMR | ^116^ |
| MOF-808 | Intrinsic | Zr sites | The adjacent lattice oxygen | Defects on MOF-808 by introducing different amounts of the second ligand to construct FLPs sites. | hydrogenation of styrene | Experiments; XRD | ^157^ |
| UiO-66(Zr) | Intrinsic | Zr^3+^ sites | –OH | In situ construction of FLP by introducing acetic acid as a modulator to give rise to a missing ligand. The unsaturated metal sites are LA, and the adjacent hydroxyl groups are LB | Photocatalytic CO_2_ reduction to CO | EPR; FTIR; XPS | ^113^ |
| Zr-based MOF | Intrinsic | Zr sites | Zr-OH | Incorporating oxygen defects into a Zr-based metal–organic layer and employing Lewis basic proximal surface hydroxyls for the in situ formation of solid heterogeneous FLPs (Zr^4-δ^–VO–Zr–OH). | CO_2_ reduction with H_2_O | XAS; DFT | ^158^ |
| Zr-based MOF | Introduction of FLP | B-functional linker | Amine substrate | The in situ formation of FLP in MOF upon the addition of basic substrates during the catalysis process | CO_2_ Chemical Fixation | XPS; NMR; DFT | ^112^ |
| UiO-67 | Introduction of FLP | B atom | N atom | Establish a linear relationship between CO_2_ hydrogenation barriers and H_2_ dissociation energy influenced by FLP acidity. | CO_2_ hydrogenation to formic | DFT | ^159^ |
| UiO-67-bpydc (Ru/bpy) | Introduction of FLP | Ru^+^ (in Ru^+^–N^-^) | N^-^ (in Ru^+^–N^–^) | Upon light illumination, charge polarization of the anchored Ru bipyridine complex can form a transient LA-LB pair, Ru^+^–N^-^ via metal-to-ligand charge transfer | H_2_ activation | DFT; FTIR; NMR | ^108^ |
| MOF,[Eu(tctb)_3_(H_2_O)] | Between adsorbed molecule and MOF | tctb^3-^ | NH_3_ | Bulky Lewis acidic boron centres were incorporated into a MOF, utilizing their electrophilicity to adsorb Lewis basic NH_3_. The steric hindrance around the LA prevents strong binding with the LB, forming FLP and facilitating a recyclable adsorption-release cycle for NH_3_. | Recyclable NH_3_ adsorption | XRD; FTIR | ^160^ |
| COF | Introduction of FLP | B(C_6_F_5_)_3_ | Triaryl phosphorus | Anchoring triaryl phosphorus (LB) in two kinds of bromine-functionalized COFs by the Cu^I^ catalysed C–P cross-coupling, then introducing tris(pentafluorophenyl)boron as the LA of FLPs, results in COF-supported heterogeneous FLP catalysts. | hydrogenation of alkynes with H_2_ | Experiments; NMR | ^104^ |
| COF | Introduction of FLP | B(C_6_F_5_)_3_ | Chiral 4,5-dihydro-4-phenyl-2-oxazole methanol (R_2_*OH) | Introducing bulky chiral LB into COFs functionalized with carboxylate groups, followed by coupling bulky achiral LA with the chiral LB to create molecular CFLPs within the COFs. | Asymmetric Olefin Hydrogenation | FTIR; DFT | ^105^ |
| COF | Introduction of FLP | [B(C_6_F_5_)_2_]^+^[Al_2_Cl_7_]^-^ | tBu_3_P (Tri-tert-butylphosphine) | COFs with C=N bonds provide opportunities to anchor sterically hindered boron cations in their pores via N→B coordination, enabling the creation of heterogeneous FLP in boron-cation-loaded COFs. | Phenyl cyclic carbonate with phenyl ethane and CO_2_ | SSNMR; FTIR | ^161^ |
| COF | Between bridged B and support COF | Bulky linkers | N sites (in TPA-COF) | New materials of FLP by in-situ assembly on alkaline COF with different LA. Acid strength and molecular size relate positively and negatively to FLP performance. | Hydrogenation of 4-octyne to Z-4-octene | FTIR; NMR | ^162^ |
| Zeolite | Introduction of FLP | Polymethylbenzenium | Si-O-Al^-^ | The H-H bond in H_2_ and C-H bonds in alkanes (n≥3) can be activated by a FLP in zeolites, which consists of hydrocarbon pool species (LA) and deprotonated zeolites (Si-O-Al^-^, LB). | Alkanes dehydrogenation | Experiments; DFT | ^48^ |
| Zeolite | Adsorption-induced FLP | Al atom | O atom in silanol group | LA and LB in SAPO zeolites can be transiently created due to induced FLPs via competitive adsorption of adsorbate molecules | Adsorption polar molecules | SSNMR; SXRD; NPD; DRIFT | ^45^ |

**Table S2**. A brief summary of heterogenous FLP construction, application, and application on metal oxides in recent years.

| Category | Catalysts | Active sites | FLA | FLB | Discovery | Catalytic Application | Key evidence | Ref |
| --- | --- | --- | --- | --- | --- | --- | --- | --- |
| Metal hydroxides | Boehmite (AlOOH) surfaces | Intrinsic | Adjacent unsaturated Al site | OH_v_ site | An efficient solid surface FLP site was constructed on an inert metal oxide for hydrogenation reactions. | H_2_ activation for styrene hydrogenation to ethylbenzene | XPS; FTIR; DFT | ^164^ |
| Metal/metal oxides | Ru-doped MgO | Metal dopant and support | Ru dopant | O from MgO support | Construct metal/support FLPs to enhance the hydrogenation of N-heteroaromatics and S-heteroaromatics through heterogeneous hydrogen dissociation and ionic hydrogenation mechanisms. | H_2_ activation for hydrogenation of mono- and poly-cyclic arenes, N-heteroaromatics, and S-heteroaromatics | Experiments | ^88^ |
| Metal/metal oxides | Ru-Clusters/Ceria | metal clusters and support | O_v_ | O of Ru-O-Ce linkage | FLP active sites in Ru/ceria catalyst achieved a highly active ethylene methoxycarbonylation reaction. | Ethylene Methoxycarbonylation | NMR, DFT; pyridine-IR | ^174^ |
| Metal oxides | Porous nanorods of ceria (PN-CeO_2_) | Intrinsic | Two adjacent Ce^3+^ ions | Lattice O^2–^ adjacent to surface Ov | PN-CeO_2_ with a high concentration of surface O_v_ construct new LA sites by two adjacent surface Ce^3+^ and the neighbouring surface lattice oxygen as LB, the FLPs can easily dissociate H–H bonds with low activation energy. | Hydrogenation of alkenes and alkynes | DFT; Raman | ^63^ |
| Metal oxides | CeO_2_ | Intrinsic | Adjacent unsaturated Ce site | Hydroxyl adjacent to surface Ov | DFT calculations indicate that the H intermediate from hydrogen heterolysis is stabilized by the Ce cation in FLP, rather than transferring to the adjacent surface O anion. | H_2_ activation for hydrogenating alkenes and alkynes | DFT | ^56^ |
| Metal oxides | CeO_2_ | Intrinsic | Adjacent unsaturated Ce site | Hydroxyl adjacent to surface Ov | DFT studies indicate that the formation of solid FLPs on CeO_2_(110) is dependent on the number of O_v_. | Nonoxidative Coupling of Methane | DFT | ^62^ |
| Metal oxides | CeO_2_ | Intrinsic | Two adjacent Ce^3+^ ions | Lattice O^2–^ adjacent to surface Ov | CO_2_ activation occurs via a bidentate carbonate bridging the FLP, involving a Ce^3+^-to-CO_2_ charge transfer that enhances activation. | CO_2_ and CH_3_OH Conversion to Monomethylcarbonate | XPS, IR, UV–vis | ^176^ |
| Metal oxides | CeO_2_ | Intrinsic | Two adjacent Ce^3+^ ions | Lattice O^2–^ | Two adjacent (Ce^3+^···O^2–^) LA and LB effectively activate CO_2_. | Selective transformation of olefins and CO_2_ into cyclic carbonates | DFT; TPR | ^177^ |
| Metal decorated  Metal oxides | Porous CeO_2_ nanorods (Pt cluster/PN−CeO_2_) | Intrinsic FLP and Pt for H_2_ cleavage | Adjacent unsaturated Ce site | Hydroxyl adjacent to the surface O_v_ | The dual-active sites of Pt clusters and FLPs on CeO_2_ offer an interface-independent pathway to enhance the reverse water-gas shift reaction at low temperatures. | Reverse Water-Gas Shift Reaction | XPS; DRIFT; DFT; KIE for hydrogen spillover | ^118^ |
| Non-metal doped Metal oxides | Nitrogen-incorporated CeO_2_ | Intrinsic | Adjacent unsaturated Ce site | O site adjacent to the surface Ov | N-doped CeO_2_ can induce the reduction of Ce^4+^ to Ce^3+^, further enhancing the Lewis acidity. | Photocatalytic CO_2_ reduction | XPS; DFTIR; DFT | ^172^ |
| Metal decorated  Metal oxides | Ni/CeO_2_ | Intrinsic | O_v_ | Hydroxyl adjacent to the surface O_v_ | The (110) facet of Ni/CeO_2_, enriched with O_v_ and hydroxyl species, creates abundant FLP active sites, enhancing low-temperature activity and stability for CO_2_ methanation. | CO_2_ Methanation | DRFTI; DFT | ^150^ |
| metal doped  Metal oxides | Ce doped BiOBr | Intrinsic | Ce^4+^ | Lattice O^2–^ | Ce^4+^ substitutes Bi^3+^ in the lattice of BiOBr. Ce^4+^ and O^2–^ form a strong LA/LB pair, Ce^4+^-O^2–^, which enhances the capture and activation of CO_2_ | Photocatalytic Reduction of CO_2_ with H_2_O | XPS, XAFS, FTIR; DFT | ^178^ |
| Metal/metal oxides | Carbon-Encapsulated Ni/NiOx | Dopant assisted intrinsic | V_Ni_-C | V_O_-C | Developed a defect-rich (O_v_ and Ni (V_Ni_) vacancy sites) catalyst with high-density FLPs sites. | Photothermal-assisted photocatalytic hydrogen production | DFT | ^179^ |
| Metal oxides | CrMnFeCoNi high-entropy oxide | Intrinsic | Ov | Surface hydroxyls or surface lattice oxygen | Synthesized holey layered high-entropy oxide nanocrystals with abundant FLPs. | Catalytic transfer hydrogenation reaction of biomass-derived carbonyl compounds | XPD, TPD; ATR-IR; DFT | ^180^ |
| Metal oxides | In_2_O_3-x_(OH)_y_ | Intrinsic | In | In-OH | Direct observation of the heterolysis of H_2_ on surface FLPs of In_2_O_3-x_(OH)_y_. | Photocatalytic hydrogenation of CO_2_ | DRIFTS, NMR, EPR; | ^71^ |
| Metal oxides | In_2_O_3-x_(OH)_y_ | Intrinsic | In | In-OH | The rhombohedral polymorph surpasses the cubic polymorph in catalytic activity, stability, and selectivity for CH_3_OH due to enhanced acidity and basicity of the surface FLP. | Photocatalytic CO_2_ hydrogenation | DFT | ^165^ |
| Metal doped  Metal hydroxides | Bi doped In_2_O_3_-x(OH)y | Intrinsic | In | In-OH | At the optimal Bi^3+^ substitution level, the 6s² electron pair of Bi^3+^ hybridizes with oxygen at the adjacent In-OH Lewis base site, slightly enhancing Lewis basicity without altering the Lewis acidity of the nearby In LA site. | Photocatalytic Reduction of CO_2_ | DFT | ^166^ |
| Metal doped  Metal oxides | Bi doped In_2_O_3_ (Bi_x_In_2–x_O_3_) | Dopant assisted intrinsic | Bi^3+^ | O^2-^ | Achieved atom-precise substitution of Bi^3+^ for In^3+^ sites in In_2_O_3_ and realized the tailor of the reactivity of surface FLP. | Photocatalytic CO_2_ hydrogenation | XAS; DRIFTS; DFT | ^167^ |
| Metal hydroxides | InOOH | Intrinsic | Unsaturated In sites | Surface hydroxyl | The annealing process breaks the In–O bonds in InOOH, generating sterically hindered unsaturated indium sites and adjacent hydroxyl groups, which together form active FLP sites. | Electrochemical N_2_ and CO_2_ conversion to urea | TPD; DFT | ^181^ |
| Perovskite | Cs_2_CuBr_4_ Perovskite Quantum Dots | Intrinsic | Cu central atom surrounded by Br atoms | Sterically isolated Cs atoms | The surface spontaneous polarization effect of Cs_2_CuBr_4_ arises from its unique microelectronic structure, creating surface LA and LB sites that form surface FLP-like active sites. | CO_2_ Photoreduction | DRIFTS, DFT | ^182^ |
| Metal oxides | Lamellar Nb_2_O_5_ | Intrinsic | Unsaturated Nb site | Nb-OH | High-density FLP active sites were fabricated on layered Nb₂O₅ by thermal reduction, thereby achieving efficient photocatalytic anaerobic coupling of methane. | Photocatalytic non-oxidative methane coupling | DFT | ^68^ |
| Metal oxides | SrBi_2_Ta_2_O_9_ (SBT) | Intrinsic V_O_-Bi-OH | O_v_ | Proximal surface hydroxyl | O_v_ reacts with adsorbed H_2_O to form renewable surface hydroxyl groups. The O_v_ and adjacent -OH act as FLP active sites for CO_2_ adsorption, activation, and conversion. | Photocatalytic CO_2_ reduction to CO | EPR; XPS; TPD; FTIR | ^168^ |
| Dual-metal hydroxide | CoGeO_2_(OH)_2_ | Photon assisted intrinsic | Photoinduced O_v_ | Proximal surface hydroxyls | Under irradiation, photogenerated holes oxidize surface hydroxyls on CoGeO_2_(OH)_2_, creating O_v_ and protons. These O_v_ (LA) and adjacent hydroxyls (LB) form FLPs, which capture, activate, and reduce CO_2_ to CH_4_ with the assistance of protons. | Photocatalytic CO_2_ Reduction to CH_4_ | EPR; FTIR | ^183^ |
| Dual-metal hydroxide | ZnSn(OH)_6_ | Photon assisted intrinsic | O_v_ | Light-stable hydroxyls Zn-OH | The light-induced O_v_ and their proximal terminal hydroxyl groups co-constitute the FLPs, which significantly improved photocatalytic CO_2_ reduction performance | Photocatalytic CO_2_ Reduction to CO | EPR; XPS | ^107^ |
| Metal hydroxides | ZnIn_2_S_4_/In(OH)_3_–x heterojunction | Intrinsic | Adjacent unsaturated In site | Hydroxyl adjacent to surface hydroxyl-deficient vacancies | Light-induced hydroxyl-deficient vacancies in In(OH)_3_–x function as LA, while adjacent hydroxyl groups act as LB, forming FLPs that significantly enhance photocatalytic CO_2_ reduction performance. | Photocatalytic CO_2_ Reduction | FTIR, DFT | ^168^ |
| Metal oxides | In_2_O_3_ | Introduction of LP | In atom (in O-[O_v_]-In-H^-^) | O atom (in In-OH^+^) | Upon irradiation, active FLPs of In-OH^+^ and In-[O_v_]H- are generated on the In_2_O_3_ surface via H_2_ splitting. | Dehalogenation of aryl halides | NMR; H/D KIEs | ^184^ |
| Metal oxides | Zr single atoms supported N-doped TiO_2_-x (Zr-TiON) | Intrinsic | Unsaturated Zr site | O_v_ | Unsaturated Zr single atoms (LA) and surrounding O atoms at oxygen vacancies (LB) enable selective adsorption and activation of electron-rich NO₃⁻ and electron-deficient *H species. | Electrochemical reduction of nitrates (NO_3_RR) | DFT | ^185^ |
| Metal oxides | Hollow TiO_2_-x boxes | Intrinsic | O_v_ | proximal surface hydroxyls | Fabricate FLPs on hierarchical hollow TiO_2_-x boxes via in situ topological transformation of perovskite microcubes. | Photocatalytic CO_2_ reduction | FTIR; DFT | ^66^ |
| Amorphous TiO_2_–x(OH)y | c-TiO_2_@a-TiO_2_-x(OH)y | Intrinsic | HO-Ti-[O_v_]-Ti | HO-Ti-[O_v_]-Ti | HO-Ti-[O]-Ti surface FLPs embedded in core-shell c-TiO_2_@a-TiO_2_-x(OH)y heterostructure for a new genre of chemical reactivity. | Photocatalytic CO_2_ hydrogenation to HCOOH | DRIFT, NMR, DFT | ^64^ |
| Amorphous TiO_2_–x(OH)y | Amorphous TiO_2_–x(OH)y coated CuPt Alloy | Intrinsic FLP for CO_2_ and CuPt for H_2_ cleavage | Ti | Ti-OH | Amorphous TiO_2_–x(OH)y with a CuPt alloy on its surface was constructed to investigate the synergy between surface FLPs and the metal cocatalyst technique. | CO_2_ hydrogenation | EPR, XPS | ^186^ |

**Table S3**. A brief summary of heterogenous FLP construction, application, and application on non-metal supports in recent years.

| Category | Catalysts | Active sites | FLA | FLB | Discovery | Catalytic Application | Key evidence | Ref |
| --- | --- | --- | --- | --- | --- | --- | --- | --- |
| Carbon nitride | Boron and sulphur co-doped graphitic carbon nitride | Dopant and support/ Intrinsic | Electron-deficient S atom | Electron-rich N atom adjacent to B | FLPs shift the rate-determining step from CO_2_ protonation to CO_2_ adsorption. The "push-pull" effect on FLPs enables the spontaneous formation of *COOH and *CO intermediates by balancing the *COOH adsorption energy. | Photocatalytic carbon dioxide reduction | DFT | ^190^ |
| Carbon nitride | Boron and oxygen dual-doped carbon nitride | Dopant assisted intrinsic | B atoms | N atoms | Boron/oxygen-induced coordination enhances electron transfer rates, while the FLPs formed by boron and nitrogen atoms activate N_2_ or nitrate, accelerating NH_3_ generation kinetics. | Electrocatalytic ammonia synthesis from nitrogen gas | FTIR, XPS | ^191^ |
| Carbon nitride | Mg-doped g-C_3_N_4_ | Dopant assisted intrinsic | Mg atom | N atom | FLPs were constructed by inserting electron-deficient magnesium into g-C_3_N_4_. | Photocatalytic N_2_ reduction to NH_3_ | FTIR | ^192^ |
| Carbon nitride | W-doped g-C_3_N_4_ | Dopant assisted intrinsic | W single-atom (W_SA_) | N atom (in W–N–C–N) | The N·WSA FLP adsorbs CO_2_ to form an unusual W–O–C–N structure characterized by significant d-p orbital interactions, resulting in a distinctive "push–push" electron transfer effect. | Photocatalytic CO_2_ reduction to CO | XAS, DFT | ^86^ |
| Carbon nitride | B-doped amorphous C_3_N_4_ | Dopant assisted intrinsic | B atoms  (in surface -B(OH)_2_) | N atom  (in (OH)_2_B–N–C–NH_x_) | Constriction of FLP on 2D amorphous C_3_N_4_ via the introduction of boric acid with supercritical CO_2_. The FLP active sites can help efficiently adsorb, activate, and reduce CO_2_ to CH_4_. | Photocatalytic CO_2_ Reduction to CH_4_ | XPS, FTIR, DFT | ^193^ |
| Carbon nitride | B doped CN | Dopant assisted intrinsic | B atoms | N atoms | FLPs on carbon nitride can capture, activate, and reduce N_2_ to NH_3_ through a "pull–push" effect. | N_2_ reduction to NH_3_ | TPD, FTIR | ^194^ |
| h-BN | Hexagonal boron nitride | Defect assisted Intrinsic | B atoms | N atoms | FLP on h-BN contributes to highly efficient H_2_/D_2_ activation and dissociation under ambient pressure via FLP-like behaviours. | Styrene hydrogenation | XAFS, NMR | ^72^ |
| Ceramic | Na-B-PSZ (Sodium-Doped Amorphous Si-B-N Ceramic) | Dopant assisted intrinsic | B atoms | N atoms | Na^+^ and B^III^ were incorporated into an amorphous silicon nitride network, where Na^+^ and B^III^ moieties, surrounded by SiN_4_ units, facilitated the transformation of the B^III^ into 4-fold coordinated geometry upon encountering H_2_, potentially serving as LA sites. | Hydrogen activation | FTIR, NMR | ^195^ |
| Polymer | B-N containing phenolic organic microsphere | Intrinsic | B atoms | N atoms | Incorporate LA sites into the framework of organic polymer microspheres through benzoxazine ring-opening polymerization. | Selective alkyne hydrogenation to cis-alkenes | DFT, NMR, FTIR | ^196^ |
| Carbon nitride | Defective boron carbon nitride (BCN) | Introduction of LP/ Intrinsic | Unsaturated B atoms | Unsaturated N atoms | Developed a defective boron carbon nitride catalyst featuring abundant electron-deficient boron atoms and electron-rich nitrogen atoms as LA/LB sites, enhancing the electrocatalytic reduction of N_2_ to ammonia. | Electrocatalytic nitrogen reduction to ammonia | ^14^N_2_/^15^N_2_ exchange experiment, DFT | ^85^ |
| Carbon nitride | B/C_2_N | Dopant assisted intrinsic | B atoms | N atoms | An FLP photocatalyst was prepared by decorating a single boron atom on monolayer C_2_N. The B-N FLP, with its pull-push function, effectively converts CO into C_2_H_4_ and C_3_H_6_. | Photocatalytic reduction of CO to multi-carbon chemicals | DFT | ^197^ |
| Carbon nitride | Co-doped carbon nitride (Co@CN) | Dopant assisted intrinsic | Co atoms | N atoms | H₂ can be heterolyzed at the Co–N bond, forming OH^δ⁻^–Co–N–H^δ⁺^, which reduces the activation energy for hydration reactions. | Hydration of alkenes and epoxy alkanes | ATR-IR, DFT | ^50^ |
| Nanodiamond-based carbon | Nitrogen and boron incorporated graphite carbon materials | Dopant assisted intrinsic | Surface-doped electron-deficient B atoms | Surface-doped electron-rich N atoms | Solid-phase metal-free Lewis pairs, consisting of electron-rich nitrogen and electron-deficient boron, were dispersed on sp²-hybridized diamond surfaces. These unquenched Lewis pairs split H₂ into H⁺ and H⁻, enabling the catalytic hydrogenation of various substrates. | H_2_ activation for cyclooctene hydrogenation | DFT | ^198^ |
| P monolayer | Phosphorene monolayer doped with B impurity | Dopant assisted intrinsic | B atoms | P atoms | Use DFT calculations to design a heterogeneous catalyst by embedding boron dopants into a 2D phosphorene monolayer, forming B/P Lewis pairs for hydrogenation reactions. | Hydrogenation of small unsaturated molecules | DFT | ^199^ |
| Carbon nitride | Boron-doped 3D carbon nitride | Intrinsic | unsaturated B atoms | unsaturated N atoms | The honeycomb 3D structure increases nitrogen defect exposure, while abundant B/N FLP enhance N_2_ chemisorption and activation, reducing the nitrogen reduction reaction (NRR) barrier and suppressing hydrogen evolution. | Electrocatalytic nitrogen reduction | TPD, XPS | ^200^ |
| Polymer | Poly (heptazine imide) polymer （PHI） | Introduction of LP | H atoms | N atoms | Incorporate surface hydroxyl groups into the chemical structure of PHI, where the hydrogen atoms in the hydroxyl groups form FLP with nitrogen atoms in terminal cyano groups (-C≡N) through hydrogen bonding, acting as "steric hindrance." | CO_2_ Photoreduction | DFT | ^115^ |
| Polymer | [Ar_3_PH][HB(C_6_F_5_)_3_] | polymeric LP/Intrinsic | B(C_6_F_5_)_3_ | PAr_3_ (Ar=*o*-C_4_H_3_ME, C_6_H_2_Me_3_) | Report the first synthesis of two semi-immobilized FLPs with basic components embedded in the backbones of microporous polymer networks via Yamamoto polymerization, and investigate their interactions with the strong LA of B(C_6_F_5_)_3_. | Room-temperature activation of hydrogen | H/D KIEs | ^201^ |

**Table S4**. A brief summary of applications of diverse FLP systems on thermos, electro and photo-catalysis in recent years.

| **H_2_-related activation** | | | | |
| --- | --- | --- | --- | --- |
| Applications | Catalysis type | FLP type | Catalysts and FLP systems [LA/LB] | Additional points |
| H_2_ activation | Thermo | Intrinsic FLP | In_2_O_3-x(_OH)_y_ – [In/OH] ^71^ |  |
|  |  |  | Metal sulfides ^208, 209^ |  |
|  |  |  | CeO_2_ ^56, 210^ |  |
|  |  |  | MgO ^207^ | HX activation (*X* = OH, H, SH, NH_2_) |
|  |  |  | Al_2_O_3_ ^32, 55^ | H_2_ and CH_4_ activation |
|  |  |  | Wurtzite structure ^35^ | Small molecule activation (e.g., H_2_, CH_4_, NH_3_, H_2_S, and PH_3_) |
|  |  | Modified FLP (impregnation) | Pt/NaY – [Na/O] ^44^ |  |
|  |  |  | BN-graphene – [C/C] ^37^ |  |
|  |  |  | Ru/MgO(111) – [Ru/O_support_] ^89^ | Small molecule activation (e.g., H_2_, NH_3_) |
|  |  | Modified FLP (Organics) | Silica – [B/P] ^98^ |  |
|  |  |  | Triphenylphosphine motifs – [B/P_support_] ^201^ |  |
|  |  | Induced FLP | FAU zeolite with PAH – [EFAl-Al^3+^/DHA] ^122^ |  |
|  | Photo | Induced FLP | Ru-UiO-67-bpydc ^108^ |  |
| Selective Acetylene Hydrogenation | Thermo | Intrinsic FLP | Wurtzite structure ^228^ |  |
|  |  |  | TiO_2_ ^211^ |  |
|  |  |  | Gr and GrO ^36^ | Selective acetylene hydrogenation and alkene hydrogenation |
|  |  | Modified FLP (substitution) | Ni-CeO_2_ – [Ce/O] ^75, 76^ |  |
|  |  |  | Gd-CeO_2_ – [Ce/O] and [Gd/O_support_] ^78^ |  |
|  |  | Modified FLP (impregnation) | Ni/C_2_N – [Ni/N] ^93^ |  |
|  |  |  | B or Al-Doped carbon nitrides – [B or Al/N_support_] ^229^ |  |
|  |  | Modified FLP (Organics) | POM-based NENU-3 – [Cu_support_/O] ^152^ |  |
| Selective Hydrogenation of Alkynes | Thermo | Modified FLP (Organics) | Gold – [Au_support_/N] ^96^ |  |
|  |  |  | Bromine functionalized covalent organic frameworks (COFs) – [B/P] ^104^ |  |
| Hydrogenation of cinnamaldehyde | Thermo | Intrinsic FLP | Defective UiO-66 ^74^ |  |
| Hydrogenation of alkene ^63^  Selective Hydrogenation of Crotonaldehyde ^230^ |  |  | CeO_2_ ^63, 230^ |  |
| Selective hydrogenation ^59^  Styrene activation ^60^ |  |  | Al_2_O_3_ ^59, 60^ |  |
| Hydrogenation of olefins and alkynes |  |  | AlOOH ^164^ |  |
| Hydrogenation of styrene |  |  | h-BN ^72^ |  |
| Nitro compound hydrogenation reaction | Thermo | Modified FLP (substitution) | 2D-COFs-drived B/N co-doped carbon nanosheets – [B/N] ^231^ |  |
| Hydrogenation of styrene and olefins |  |  | CoBOx ^232^ |  |
| Hydrogenation of Phenylacetylene |  |  | BN-NCN ^73^ |  |
| Hydrogenation of mono- and poly-cyclic arenes, N-heteroaromatics, and S-heteroaromatics | Thermo | Modified FLP (impregnation) | Ru/MgO – [Ru/O_support_] ^88^ |  |
| Selective hydrogenation of α, β-unsaturated aldehydes |  |  | B/N co-doped carbon – [B/N] ^233^ |  |
| Hydrogenation of alkenes and epoxy |  |  | Co-NC – [Co/N] ^50^ | H_2_-H_2_O system |
| Conversion of Furfurals into Linear Ketones |  |  | Pd/Ti_3_AlC_2_ – [Pd/O_support_] ^51^ | H_2_-H_2_O system |
| Selective Deoxygenation of carbonyl groups |  |  | Pd/NC – [Pd/N] ^87^ |  |
| Hydrogenation of Halogenated Nitroarenes |  |  | Ni-CeO_2_/SiO_2_ – [Ni/O_CeO2_] ^92^ |  |
| Hydrogenations of ketones, nitrile, and ethylene |  |  | B or Al-Doped phosphorenes – [B or Al/P_support_] ^199^ |  |
| Selective Deoxygenation of Epoxides |  |  | Au/HT – [Au/HT] ^90^ |  |
| Chemoselective reduction of aldehydes, epoxides and alkynes |  |  | Au@CeO_2_/HT – [Au/HT] ^91^ |  |
| Selective hydrogenation of heteroarenes |  |  | Ru/HT-C12A7 – [Ru/HT] ^234^ |  |
| Selective reduction of fatty acids to alcohols |  |  | Ru/NH_2_-rGO – [Ru/rGO] ^38^ |  |
| Hydrotreating reactions |  |  | P-doped NiAl-oxide – [P/Ni] ^84^ |  |
| Z-selective hydrogenation of 3-hexyne | Thermo | Modified FLP (Organics) | Silica – [B/P] ^97^ |  |
| Hydrogenation of Alkenes |  |  | Polyamine – [B/N_support_] ^235^ |  |
| Hydrogenation Imine/nitrile |  |  | Gold – [Au_support_/N] ^95^ |  |
| Reductive Deoxygenation of Ketones and Aldehydes |  |  | α-CD or MS – [B/O_support_] ^236^ |  |
| Asymmetric olefin hydrogenation |  |  | Chiral COF – [B/N] ^105^ |  |
| Hydrogenation of Alkylidene Malonate Compounds ^100^  Transfer hydrogenation of alkenes, aldehydes, ketones, and imines ^237^  Regioselective hydrogenation of imine ^102^  Selective hydrogenating α,β-unsaturated imine ^43^, and ketones ^238, 239^ |  |  | MIL-101(Cr) – [B/N] ^43, 100, 102, 237^, [B/O] ^238^, [B/P] ^239^ |  |
| Selective Hydrogenation of N-Heterocycles ^42^  Selective Reduction of Nitroolefins to Nitroalkanes ^103^ |  |  | NU-1000 – [B/N] ^42, 103^ |  |
| Selective hydrogenation of 4-octyne to octene |  |  | TPA-COF with acids – [B/N] ^162^ |  |
| **CO_2_ related activation** | | | | |
| Applications | Catalysis type | FLP type | Catalysts and FLP systems [LA/LB] | Additional points |
| Reduction of CO_2_ | Thermo | Modified FLP (substitution) | Ni-CeO_2_ – [Ce/O] ^77^ | RWGS |
|  |  | Modified FLP (impregnation) | Pt/PN-CeO_2_ – [Ce/O] ^118^ | RWGS |
|  |  |  | Na-doped NiLa(BDC) – [Ni/O] ^240^ | RWGS and methanation |
|  |  |  | Ni-CeO_2_ – [Ce/O] ^171^ | Methanation |
|  |  |  | Al@N-Gr-2 – [Al/N] ^215^ | Reduction to formic acid |
|  |  | Modified FLP (Organics) | UiO-66-P-BF_2_ – [B/N] ^41^ | Reduction to formic acid |
|  |  |  | UiO-67-NBF_2_ – [B/N] ^40^ | Reduction to formic acid and methanol |
|  |  |  | BCF@MOF-545 – [B/N] ^101^ | Reduction to methanol |
|  | Photo | Intrinsic FLP | In_2_O_3-x_(OH)_y_ – [In/OH] ^33, 57, 58, 225^ | RWGS (H_2_) |
|  |  |  | ZnSn(OH)_6_ – [Sn/Zn-OH]^107^ | RWGS (H_2_); H_2_ is generated during hole oxidation of OH^-^ |
|  |  |  | c-TiO_2_@a-TiO_2-x_(OH)_y_ – [Ti/OH] ^64^ | RWGS (H_2_); heterostructure |
|  |  |  | Defective UiO-66(Zr) – [Zr/OH] ^113^ | RWGS (water) |
|  |  |  | Zr-MOL-D – [Zr/OH] ^158^ | RWGS (water) |
|  |  |  | TiO_2-x_ – [Ti^3+^ and hydroxyl groups] ^66^ | RWGS (water) |
|  |  | Modified FLP (substitution) | Bi-In_2_O_3-x_(OH)_y_ – [In-O] ^166^ | RWGS (H_2_) |
|  |  |  | In_2_O_3−x_(EDA)_y_ – [In/N] ^99^ | RWGS and methanol formation (H_2_) |
|  |  |  | Sn-ultrathin BiOCl – [Bi/O] ^226^ | RWGS (water) |
|  |  |  | N-In_2_O_3_ – [In/O] ^81^ | Methanol formation (water) |
|  |  |  | N-CeO_2_ – [Ce/OH] ^172^ | Reduction to C_2_ hydrocarbon (water) |
|  |  |  | poly(heptazine imide) ^115^ | RWGS (TEOA); Incorporation of OH |
|  |  | Modified FLP (impregnation) | W single-atoms doped polymeric carbon nitride – [W_SA_/Ni] ^86^ | RWGS (water) |
|  |  | Modified FLP (substitution) and induced FLP | Pt/TiN_x_O_y_ – [Ti/NH_2_] ^114^ | RWGS (H_2_)  Induced by H_2_ cleavage |
| CO_2_ with methanol to form monomethylcarbonate (MMC) | Thermo | Intrinsic FLP | CeO_2_ – [Ce/O] ^176^ |  |
| Propylene epoxide cycloaddition with CO_2_ |  |  | MgO – [Mg/O] ^67^ |  |
| Transfer hydrogenation to form amide |  |  | Copolymers ^189^ |  |
| Co-conversion of CH_4_ and CO_2_ to acetic acid | Thermo | Modified FLP (substitution) | Metal_1_-CeO_2_ – [Ce/O] ^119^ |  |
| Co-conversion of CH_4_ and CO_2_ to acetic acid |  |  | Cu-In_2_O_3_ – [Cu/O] ^216^ |  |
| Transformation of styrene and CO_2_ to phenyethylene |  |  | La_1_-CeO_2_ – [(La,Ce)/O] ^80^ |  |
| CO_2_ with *o*-phenylenediamines to high-valued benzimidazole derivatives | Thermo | Induced FLP | TCPB-1 with amine – [B/N_reactant_] ^112^ |  |
| **C-X activation** | | | | |
| Applications | Catalysis type | FLP type | Catalysts and FLP systems [LA/LB] | Additional points |
| Nonoxidative Coupling of Methane | Thermo | Intrinsic FLP | CeO_2_(110) – [Ce/O] ^62^ |  |
|  |  | Modified FLP (substitution) | Pt_1_/CeO_2_ – [Ce-O] ^79^ | C_2_ product |
|  |  | Modified FLP (incorporation) | Modified albite – [Si/O] ^219, 220^ | C_2_ product |
|  | Photo | Intrinsic FLP | NaBH_4_-treated Nb_2_O_5_ – [Nb/OH] ^68^ |  |
|  |  | Modified FLP (substitution) | p-type element/TiO_2_ – [p/OH] ^65^ | C_2_ product |
| C-halogen activation | Thermo | Intrinsic FLP | Alumina cluster – [Al_III_/O] ^218^ |  |
| H_2_ activation, C-H activation and propane dehydrogenation | Thermo | Modified FLP (organics) | PMB^+^ confined zeolites – [PMB^+^/O_framework_] ^48^ |  |
| Other small molecules activation | | | | |
| Applications | Catalysis type | FLP type | Catalysts and FLP systems [LA/LB] | Additional points |
| Glycerol oxidation to formic acid | Thermo | Intrinsic FLP | MnO_2-D_ – [Mn/O] ^69^ |  |
| CO oxidation |  | Modified FLP (incorporation) | Au decorated hydroxyapatite (HAP)–CeO_2_ – [Ce/O] ^221^ |  |
| NH_3_ decomposition |  |  | Ru/13X – [Ru/O] ^46^ |  |
| H_2_O and methanol activation |  |  | Metal/ZSM-5 – [M/O] ^94^ |  |
| Methanol to olefin |  | Induced FLP | SAPO-type zeolite – [Al_BAS_/O_BAS_] ^45, 47^ |  |
| Methanol oxidation reaction | Electro | Modified FLP (substitution) | Pt NPs doped Phospher-CeO_2_ – [Ce/P] ^224^ |  |
| Electrocatalytic reduction of nitric oxide (NOER) |  |  | Anion doping ZnO – [P/Zn] ^82^ |  |
| Nitrogen reduction to ammonia |  | Modified FLP (incorporation) | Defective Boron Carbon Nitride – [B/N] ^85^ |  |
| Photoelectrochemical nitrate reduction reaction (PEC NIRR) | Photo-electro | Modified FLP (heterojunction) | CeO_2_–C/BiVO_4_ – [Ce/O] ^241^ |  |
